# High-velocity fragmentation of titanium alloy rings and cylinders produced using Field-Assisted Sintering Technology

**DOI:** 10.1007/s10704-024-00829-9

**Published:** 2025-03-25

**Authors:** T. Virazels, S. Lister, O. Levano-Blanch, M. Jackson, J. A. Rodríguez-Martínez, J. C. Nieto-Fuentes

**Affiliations:** 1 Department of Continuum Mechanics and Structural Analysis, University Carlos III of Madrid, Avda. de la Universidad, 30, 28911 Leganés, Madrid Spain; 2 Department of Materials Science and Engineering, The University of Sheffield, Mappin Street, Sheffield, S1 3JD UK

**Keywords:** Titanium alloys, Field-Assisted Sintering Technology, Impact fragmentation, Ring expansion, Tube expansion

## Abstract

This paper explores the mechanics of high-velocity impact fragmentation in titanium alloys produced by Field-Assisted Sintering Technology. For that purpose, we have utilized the experimental setups recently developed by Nieto-Fuentes et al. (J Mech Phys Solids 174:105248, 2023a; Int J Impact Eng 180:104556, 2023b) for conducting dynamic expansion tests on rings and cylinders. The experiments involve firing a conical-nosed cylindrical projectile using a single-stage ight-gas gun against the stationary ring/cylinder at velocities ranging from $\approx 248~\text {m}/\text {s}$ to $\approx 390~\text {m}/\text {s}$, corresponding to estimated strain rates in the specimen varying from $\approx 10050~\text {s}^{-1}$ to $\approx 19125~\text {s}^{-1}$. The diameter of the cylindrical part of the projectile exceeds the inner diameter of the ring/cylinder, causing the latter to expand as the projectile moves forward, resulting in the formation of multiple necks and fragments. Two different alloys have been tested: Ti6Al4V and Ti5Al5V5Mo3Cr. These materials are widely utilized in aeronautical and aerospace industries for constructing structural elements such as compressor parts (discs and blades) and Whipple shields, which are frequently exposed to intense mechanical loading, including high-velocity impacts. However, despite the scientific and technological significance of Ti6Al4V and Ti5Al5V5Mo3Cr, and the extensive research on their mechanical and fracture behaviors, to the best of the authors’ knowledge, no systematic study has been conducted thus far on the dynamic fragmentation behavior of these alloys. Hence, this paper presents an ambitious fragmentation testing program, encompassing a total of 27 and 29 experiments on rings and cylinders, respectively. Monolithic and multimaterial samples—half specimen of Ti6Al4V and half specimen of Ti5Al5V5Mo3Cr—have been tested, taking advantage of the ability of Field-Assisted Sintering Technology to produce multimaterial parts. The fragments have been collected, weighed, sized, and analyzed using scanning electron microscopy. The experiments have shown that the number of necks, the number of fragments, and the proportion of necks developing into fragments generally increase with expansion velocity. The average distance between necks has been assessed against the predictions of a linear stability analysis (Zhou et al. in Int J Impact Eng 33:880–891 2006; Vaz-Romero et al. in Int J Solids Struct 125:232–243, 2017), revealing satisfactory agreement between theoretical predictions and experimental results. In addition, the experimental results have been compared with tests reported in the literature for various metals and alloys (Nieto-Fuentes et al. in J Mech Phys Solids 174:105248, 2023a; Zhang and Ravi-Chandar in Int J Fract 142:183–217, 2006, Zhang and Ravi-Chandar in Int J Fract 150:3–36, 2008) to examine the influence of material behavior on the statistics of fragments size and necks spacing.

## Introduction

The historical perspective on *metallurgical effects of high strain-rate deformation and fabrication* elaborated by Rinehart (1981) dated the first scientific experiments on dynamic fragmentation of metallic materials at the beginning of the $19^{\text {th}}$ century. French ordnance engineers started a program to detonate hollow spherical steel cannon balls under controlled loading conditions with the aim of establishing the geometrical and material properties which provide the optimal performance of the casings (Hélie 1884). The explosions were conducted in a pit with a damp clay floor, designed as a soft-recovery system to capture the ejected fragments. The velocity of the fragments was estimated by recording their penetration depth into the clay and comparing it to reference values obtained by firing pistol bullets of known velocity and mass into the same clay. The experiments revealed that the velocity and number of fragments increase with the weight of the exploding charge and decrease with the thickness and failure strength of the casing. Similar trends have been observed in nearly all fragmentation tests conducted on metallic shells over the last 200 years, highlighting the extreme care and precision with which this initial attempt to determine the mechanics of dynamic fragmentation was executed.

Over the next 100 years, research on the high-velocity impact fragmentation of metallic materials remained primarily driven by military requirements, with a notable increase in activity during World War II. A turning point was the series of reports elaborated by Sir Nevill F. Mott (Mott and Linfoot 1943; Mott 1943a, c, b) for the Ministry of Supply of the UK between January and May 1943 in which the main features of a theory to describe the process of fragmentation resulting from the explosive rupture of cylindrical structures were presented. The theory was applicable to materials which deform plastically before rupture, and provided formulas to compute the mean fragment size and the distribution of fragment sizes of certain types of steel bomb and shell. The reports were eventually compiled into a paper published in the open literature in 1947 (Mott 1947a). This article featured a one-dimensional model that considered the onset of fractures as a random process resulting from spatial variability in the strain-to-fracture of ductile materials. This variability was attributed to scattering in the local material and geometrical properties of the specimen.

After World War II, and continuing to the present day, there has been a growing development of research on the dynamic fragmentation of metallic structures, extending beyond military applications to encompass both fundamental and applied aspects of the subject. The goal is to unravel the mechanisms that control the energy absorption performance of protective structures in the automotive, aircraft, aerospace, and civilian-security industries. In these sectors, structural elements are often subjected to a wide range of unusually severe mechanical solicitations. For instance, components for satellites must be designed to withstand hypervelocity impacts of space debris (Ryan et al. 2011; Wen et al. 2020). Crashworthiness structures of ground and marine vehicles are intended to absorb energy in accidents and crashes (Paik and Seo 2007; Mujeeb-Ahmed et al. 2020). Protective structures of critical buildings, such as embassies and governmental facilities, are required to safeguard high-ranking officials and key infrastructures from blasts and attacks (Børvik et al. 2008; Aune et al. 2016). The increasing technological interest in the fragmentation of metallic structures has led to the design of modern experimental techniques conceived to perform fragmentation tests in a laboratory environment. In tandem, materials other than steel started to be investigated.

Notably, the ring expansion test developed by Niordson (1965) revolutionized the experimental investigation of dynamic fragmentation, opening the possibility to test materials at strain rates above $10^4~\text {s}^{-1}$ without the need for explosives, and under spatially uniform conditions of deformation. The technique involves expanding a thin circular ring at velocities up to several hundred meters per second through the application of transient magnetic fields. The high testing speed is achieved by rapidly discharging a current pulse from a capacitor bank into a solenoid through which the specimen is inserted. The current flowing through the solenoid creates an electromagnetic field that induces a current in the ring. The interaction of these two currents generates repulsive forces between the ring and the solenoid, resulting in the rapid radial expansion of the specimen. The nearly radial symmetry of the expansion virtually eliminates the propagation of waves throughout the circumference of the sample before the occurrence of multiple necking (in ductile metals) and fragmentation. This reveals the *true* dynamic properties of the material since the conditions of deformation are spatially uniform before necking (in ductile metals) and fracture. In addition, for rings with a large radius-to-thickness ratio, the radial stress is negligible compared to the circumferential stress, resulting in a predominantly uniaxial stress field. This essentially makes the problem one-dimensional, facilitating the interpretation of experimental findings. The expansion velocity is adjusted by varying the frequency and intensity of the current pulse discharged into the solenoid. This technique is particularly suitable for testing materials with high electrical conductivity; however, Joule heating effects induced by the high current density flowing through the ring can elevate the temperature of the specimen. Note that speeds of up to $200~\text {m}/\text {s}$ require currents of up to $20~\text {kA}$ to accelerate samples with approximately $1~\text {mm}$ thickness (Zhang and Ravi-Chandar 2006). Expanding larger specimens necessitates even higher currents, intensifying resistive heating and, consequently, thermal softening of the material, which may lead to eventual local melting. The electromagnetic scheme proposed by Niordson (1965) was later adapted by Wesenberg and Sagartz (1977) to expand thin-walled cylinders. The objective was to investigate the bi-dimensional features of multiple necking (in ductile metals) and fragmentation processes. Electromagnetically driven expansion of rings and cylinders has typically been conducted on copper, aluminum, and magnesium specimens. This is primarily because these three materials exhibit the highest electrical conductivity among structural metals (Grady and Benson 1983; Altynova et al. 1996; Tamhane et al. 1996; Zhang and Ravi-Chandar 2006, 2008, 2010; Kahana et al. 2015; Cliche and Ravi-Chandar 2018). Other metallic materials have been tested rarely. Exceptions include the ring expansion experiments conducted by Grady and Olsen (2003) with U6N uranium alloy, Janiszewski (2012) with barrel steel, and Wood et al. (2021) with tungsten heavy alloy and Inconel 718. These tests required a copper pusher ring to carry the induced electric current and launch the specimen, resulting in a reduced expansion speed due to the increased total mass that must be accelerated, encompassing both the pusher and the specimen.

An alternative to conducting fragmentation experiments with electromagnetic loading systems is the use of gas guns. Mechanical loading setups have the advantage of not imposing limitations on the materials that can be tested. For instance, Winter and Prestidge (1978) conducted an experiment in which a gas gun was utilized to expand thin-walled mild steel tubes with a thickness of $1~\text {mm}$. The test involved a hollow cylinder filled with rubber and positioned against a rigid anvil. When a short nylon rod was fired into the cylinder at a velocity of $630~\text {m}/\text {s}$, the radial momentum imparted to the specimen near the interface between the projectile and infill material caused the cylinder to bulge. The tests were recorded by high speed photography, which allowed to estimate that the range of strain rates in the specimen varied from $10^4$ to $5 \cdot 10^4~\text {s}^{-1}$. Multiple cracks initiated around the peak of the bulge, propagating along the axial direction of the cylinder and leading to the fragmentation of the specimen. The circumferential and longitudinal strains at which the cracks formed were estimated to be approximately $33\%$ and $11\%$, respectively. Similar experimental arrangement was used by Vogler et al. (2003) to investigate the fragmentation of AerMet 100 steel and U6N uranium alloy tubes. The specimens were $50.8~\text {mm}$ long with an inner diameter of $12.7~\text {mm}$, and they had different outer diameters ranging from $14.71~\text {mm}$ to $19.44~\text {mm}$. The specimen rested on a thick copper anvil backed by a foam of $19~\text {mm}$ thickness and $14~\text {mm}$ of steel, and it was filled with a $25.4~\text {mm}$ length polycarbonate solid cylinder. A two-stage gas gun was used to fire a polycarbonate projectile into the test tube, striking the polycarbonate insert. The deformation of the projectile and insert caused the test tube to expand outward, bulging and eventually breaking into multiple fragments. The projectile impact velocities ranged between $1.83~\text {km}/\text {s}$ and $1.94~\text {km}/\text {s}$. The experiments were recorded with high-speed photography and instrumented with a VISAR system and PDVF gauges to measure both the radial expansion velocity of the test tube and the fracture strain of the specimen. The evolution of the radial velocity with time exhibited a concave-downward shape, reaching a maximum value of approximately $200~\text {m}/\text {s}$ for both AerMet 100 steel and U6N uranium samples. The circumferential strain at fracture, measured near the peak of the bulge, was estimated to be $18\%$ for AerMet 100 steel. In the case of U6N uranium alloy tubes, it varied significantly from specimen to specimen, ranging between $12\%$ and $24\%$. Soft-recovery of the fragments was conducted by surrounding the specimen with foam and paper padding. The collected fragments were weighed and sized, revealing that the U6N uranium alloy tubes produced much smaller and more numerous fragments compared to the AerMet 100 steel specimens. More recently, Jones et al. (2013) modified the setup of Winter and Prestidge (1978) and Vogler et al. (2003) by placing a steel ogive inside the test tube instead of a cylindrical polymer, and the ogive was impacted by a polycarbonate projectile fired with a gas gun, deforming around the insert and driving radial expansion in the test tube. The uniform radial expansion created had a less complex interface than the previous setup of Winter and Prestidge (1978) and Vogler et al. (2003), as only one material was imparting momentum to the cylinder wall. Jones et al. (2013) tested 6061-T6 aluminum and Ti6Al4V samples. The 6061-T6 cylinders had an inner diameter of $30~\text {mm}$ and a wall thickness of $2~\text {mm}$, and the projectile impact velocity in the experiments was $\approx 915~\text {m}/\text {s}$. The tests on Ti6Al4V were performed with cylinders having $50~\text {mm}$ of inner diameter and $4~\text {mm}$ of wall thickness, and the projectile impact velocity was $\approx 1000~\text {m}/\text {s}$. The dimensions of the specimens and the impact velocities were chosen to achieve a maximum strain rate on the order of $10^4~\text {s}^{-1}$. Five identical tests were conducted with 6061-T6 aluminum, employing a high-speed camera and X-ray radiography to capture time-resolved images of the deformation of the polycarbonate projectile and its interaction with the test tube. The experiments conducted with the Ti6Al4V specimens were recorded using a high-speed camera, and a PDV system with four probes was employed to measure the radial velocity at various locations along the length of the cylinders. The fragmentation of the Ti6Al4V specimens was shown to be preceded by the formation of strain localization bands parallel to the axis of the test tubes that ultimately developed into multiple fractures. Building upon the concept proposed by Jones et al. (2013), Gant et al. (2021, 2024) designed an experiment in which a steel curved-nosed projectile is launched using a single-stage gas gun against a nearly incompressible high-density polyethylene disk. The disk is crushed onto a steel anvil, deforming and imparting radial momentum to a circular ring, which expands at a high strain rate. The experiments were conducted with a projectile impact velocity of $150~\text {m}/\text {s}$, utilizing rings made of 35NiCrMo16 high-strength steel, S455 mild steel, CuC2 pure copper, and aluminum alloy AA2017. The ring specimens had a mean diameter of $40~\text {mm}$ and a square cross-section of $1~\text {mm}^2$. Two high-speed cameras were used to record the tests, obtaining high-resolution images of the necking and fragmentation of the specimens. The time evolution of the specimens’ radial velocity was measured using a PDV system which showed that the maximum speed of the rings varied between $\approx 310~\text {m}/\text {s}$ for aluminum alloy AA2017 and $\approx 200~\text {m}/\text {s}$ for 35NiCrMo16 high-strength steel. The average number of fragments varied with the specimen, ranging from 25 for AA2017 to 11, 8, and 7 for CuC2, S455, and 35NiCrMo16, respectively, while the average strain at fragmentation was 0.16, 0.47, 0.30, and 0.18 for the corresponding materials. All the tests performed per material yielded similar results for the time evolution of the velocity profile, the number of fragments, and the samples’ ductility, showing the reliability of the setup to control the loading conditions and to provide compelling results. Very recently, Nieto-Fuentes et al. (2023a) developed an experiment which used a single-stage light-gas gun to fire a conical nosed cylindrical projectile into a thin-walled metal cylinder. The cylindrical samples were printed by Selective Laser Melting out of aluminum alloy AlSi10Mg, using two sets of printing parameters which led to two different levels of residual porosity in the specimens, $2\%$ and $6\%$, respectively. The samples were produced with two different outer diameters, $12~\text {mm}$ and $14~\text {mm}$, and two different wall thicknesses, $1~\text {mm}$ and $2~\text {mm}$. The diameter of the cylindrical part of the projectile was approximately twice that of the inner diameter of the cylindrical target. As the projectile moved forward, the target expanded, developing a trumpet-like shape, and eventually breaking into fragments. The tests were conducted with projectile impact velocities ranging from approximately $180~\text {m}/\text {s}$ to $390~\text {m}/\text {s}$, resulting in circumferential strain rates in the cylindrical target estimated to range between approximately $9000~\text {s}^{-1}$ and $23500~\text {s}^{-1}$. The experiments were recorded with two high-speed cameras, providing time-resolved information on the fragmentation mechanisms. Additionally, the recovered fragments were sized, weighed, and analyzed using X-ray tomography, revealing the influence of porous microstructure, specimen dimensions, and loading velocity on the number of fragments and the distribution of fragment sizes. Fragmentation occurred without noticeable necking, and the trajectory of cracks appeared to be guided by the large pores in the microstructure. The experimental setup of Nieto-Fuentes et al. (2023a) was shortly after adapted by Nieto-Fuentes et al. (2023b) to perform ring expansion tests. The specimen was placed over a ductile thin-walled tube made of AISI 316 L steel, which was expanded by a cylindrical conical-nosed projectile fired with a gas gun. This expansion pushed the ring radially outwards, eventually causing it to break into multiple fragments (i.e., the test specimen in Nieto-Fuentes et al. (2023a) is fabricated from wrought AISI 316 L steel in Nieto-Fuentes et al. (2023b) to serve as the pusher). Similarly to Nieto-Fuentes et al. (2023a), the rings were manufactured by 3D-printing technology out of AlSi10Mg. The samples had an outer diameter of $18~\text {mm}$ and a square cross section of $2 \times 2~\text {mm}^2$. The range of projectile impact velocities tested was the same investigated by Nieto-Fuentes et al. (2023a). The increase of the impact velocity was shown to shift the fragments size distribution towards smaller fragments, and towards narrower distributions. The evolution of the number of fragments with the impact velocity was modeled with the theory of Kipp and Grady (1985), coupled with the fracture energy criterion by Thomason (1970), and quantitative agreement with the experiments was obtained for the whole range of impact velocities investigated.

In this paper, we use the experimental setups developed by Nieto-Fuentes et al. (2023a, 2023b) to perform dynamic fragmentation tests on titanium rings and cylinders produced by Field-Assisted Sintering Technology (FAST). This manufacturing process is widely used in research laboratories as a rapid and cost-effective process to consolidate powders, and it is arousing the interest of different industrial sectors as an alternative to Hot Isostatic Pressing or conventional melt-wrought processing. Two different alloys are investigated in this work, Ti6Al4V and Ti5Al5V5Mo3Cr. These materials are extensively used in aeronautical and aerospace industries because of their high strength, excellent hardenability, fracture toughness and high fatigue resistance. However, despite the scientific and technological interest of titanium alloys, the only article cited in this introduction that included experiments on titanium is the work of Jones et al. (2013) —and only one experiment was reported therein (Bolis et al. (2013) reported a dynamic fragmentation experiment on a Ti6Al4V hemispherical shell using explosive loading, while Jones et al. (2012) investigated the blast-driven radial expansion of six Ti6Al4V rings with different length-to-thickness ratios). To the authors’ knowledge, this paper presents the most ambitious investigation on the fragmentation behavior of titanium alloys performed to date, including 27 and 29 tests on expanding rings and cylinders, respectively. The tests have been performed for expansion estimated strain rates varying from $\approx 10050~\text {s}^{-1}$ to $\approx 19125~\text {s}^{-1}$. The fragments have been collected, weighed, sized, and analyzed using scanning electron microscopy to investigate the fragmentation mechanisms. The number of necks, the number of fragments, and the proportion of necks developing into fragments have been observed to increase (slightly) with expansion velocity across the range of loading rates tested. The average distance between necks has been compared with the predictions of a linear stability analysis (Zhou et al. 2006; Vaz-Romero et al. 2017), and satisfactory qualitative agreement has been obtained between theoretical predictions and experiments. In addition, the experimental results for the number of necks and fragments have been compared with tests conducted by Nieto-Fuentes et al. (2023a) on additively-manufactured AlSi10Mg cylinders and by Zhang and Ravi-Chandar (2006, 2008) on Al 60610-O, Al 1100-H14 and Cu 10 rings. This comparison aims to investigate the influence of material behavior on the statistics of fragment sizes and neck spacings.

## Materials characterization and testing methods

This research involves manufacturing titanium alloy rings and cylinders using Field-Assisted Sintering Technology (FAST), followed by the microstructural analysis of the specimens and the execution of high-speed impact tests. Section 2.1 offers an overview of the titanium alloy grades under investigation, presenting their chemical composition, processing methodology, crystallographic microstructure, and basic mechanical properties. Section 2.2 details the impact testing setup used for the fragmentation experiments.

### Materials and specimens

Two titanium alloys were selected for this investigation: Ti6Al4V and Ti5Al5V5Mo3Cr. Both alloys are utilized in aerospace applications. For instance, Ti6Al4V is commonly employed as fan-disc and blade material for jet engines, while Ti5Al5V5Mo3Cr is used in aerostructural parts such as landing gear forgings. Ti6Al4V is an alpha $+$ beta titanium alloy, accounting for over $50\%$ of the world’s titanium use. Ti5Al5V5Mo3Cr, on the other hand, is a metastable beta alloy, with high strength or fracture toughness achievable through various heat treatments.

**Fig. 1:**
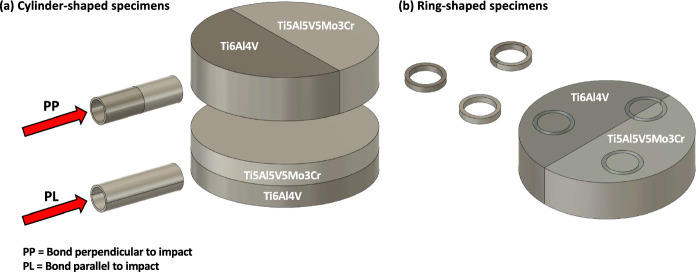
Schematic illustrating the extraction of cylinder-shaped and ring-shaped specimens from the $80~\text {mm}$ diameter billets processed via Field Assisted Sintering Technology

The ring-shape and cylinder-shape specimens investigated in this high-velocity impact fragmentation study were machined from $80~\text {mm}$ billets consolidated from powder via Field Assisted Sintering Technology (FAST) using a FCT HP D 25 FAST/SPS furnace housed at the Royce Discovery Centre of the University of Sheffield. The Ti6Al4V powder was produced by *Puris LLC* with a particle size distribution of $75 - 500~\mu \text {m}$, while the Ti5Al5V5Mo3Cr powder was manufactured by *AP* &*C* and had a particle size distribution of $45 - 100 ~\mu \text {m}$. FAST is a solid-state powder consolidation method where a pulsed DC current is applied concurrently with uniaxial pressure. This creates a joule heating effect in the graphite tooling stack causing the mould and its contents (powder) to heat up. The combined effects of heat and pressure allow full density to be achieved, with high heating rates and short processing times. The advantages of FAST over other solid-state consolidation techniques such as Hot Isostatic Pressing are that the tooling is reusable and heating occurs directly within the mould. Both the cylinders and rings were extracted from billets of either alloy that had been processed at different dwell temperatures of $970^{\circ }\text {C}$ and $1100^{\circ }\text {C}$. These processing temperatures were chosen with respect to the beta-transus temperature of Ti6Al4V $\left( \approx 990^{\circ }\text {C}\right)$, where the allotropic phase transformation between alpha and beta occurs. Therefore, by processing with these two temperatures, two different microstructures were achieved, further increasing the extent to which this study investigates the fragmentation behaviour based on chemistry and also microstructure. Monolithic samples of either alloy were machined as well as multi-material samples which contained both alloys, see Fig. 1. In the cylinders, multimaterial specimens contained a diffusion bond either perpendicular (PP) or parallel (PL) to the impact direction, as shown schematically in Fig. 1a. For the rings, samples which contained a diffusion bond were extracted alongside the monolithic alloy samples, see Fig. 1b. The ring-shaped specimens have an outer diameter of $18~\text {mm}$ and a square cross-section measuring $2 \times 2~\text {mm}^2$, while the cylinder-shaped samples have a diameter of $14~\text {mm}$, a thickness of $1~\text {mm}$, and a length of $40~\text {mm}$.

**Fig. 2:**
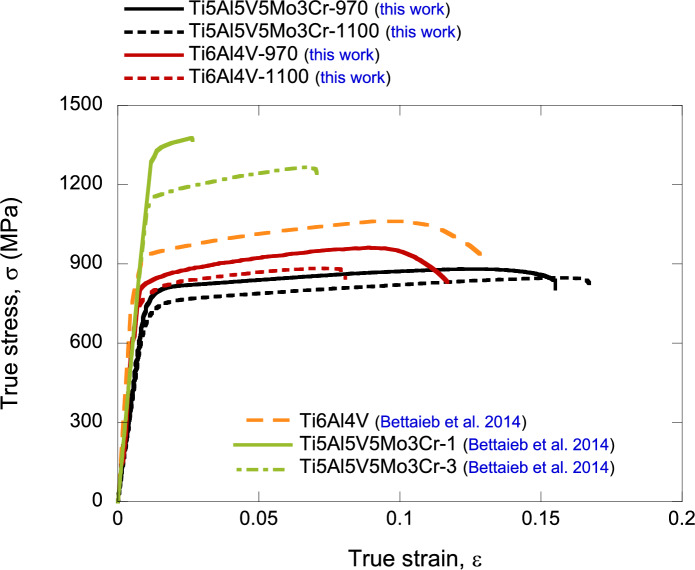
Tensile stress–strain characteristics of the titanium alloy grades investigated: Ti6Al4V-970, Ti6Al4V-1100, Ti5Al5V5Mo3Cr-970 and Ti5Al5V5Mo3Cr-1100. The numbers 970 and 1100 correspond to the dwell temperature at which the alloy was processed. Comparison with the stress–strain characteristics of forged Ti6Al4V and Ti5Al5V5Mo3Cr alloys reported by Bettaieb et al. (2014)

The tensile stress–strain characteristics of both of the alloys investigated, at each processing temperature, are shown in Fig. 2. The Ti6Al4V processed at a dwell temperature of $970^{\circ }\text {C}$, below the beta-transus, demonstrates the highest yield strength and ultimate tensile strength among the four material conditions. In contrast, Ti6Al4V processed at a dwell temperature of $1100^{\circ }\text {C}$, above the beta-transus, shows lower ultimate tensile strength, yield strength, and elongation. For Ti5Al5V5Mo3Cr, the sample processed at $970^{\circ }\text {C}$ has higher strength, but lower elongation than the sample processed at $1100^{\circ }\text {C}$. In this case, both samples were processed within the beta region, and therefore, the higher temperature sample had enhanced grain growth, leading to an increase in the elongation to failure at the expense of reducing the material flow strength. Fig. 2 compares the stress–strain characteristics of the Ti6Al4V and Ti5Al5V5Mo3Cr alloys studied here with those reported by Bettaieb et al. (2014), which were produced by forging and tested in uniaxial tension under quasi-static loading conditions. The FAST-manufactured Ti5Al5V5Mo3Cr alloys studied demonstrate lower yield stress but notably higher ductility, whereas the FAST-manufactured Ti6Al4V alloys exhibit slightly reduced flow strength with comparable elongation (for the case of Ti6Al4V-970).

**Fig. 3:**
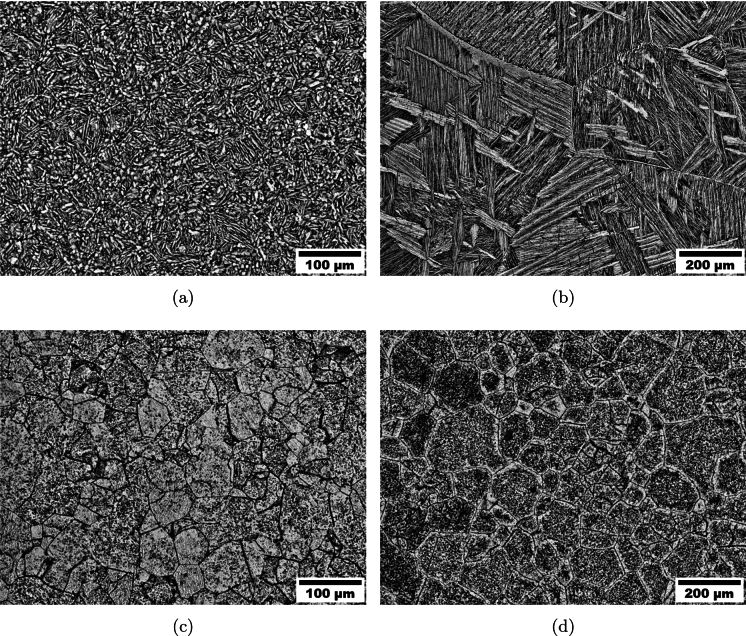
Light optical micrographs of FAST-manufactured titanium alloys: **a** Ti6Al4V-970, **b** Ti6Al4V-1100, **c** Ti5Al5V5Mo3Cr-970 and **d** Ti5Al5V5Mo3Cr-1100. The numbers 970 and 1100 correspond to the dwell temperature at which the alloy was processed

The samples were subjected to a standard titanium alloy preparation routine before being etched with Kroll’s Reagent to reveal the microstructure under optical light microscopy. The microstructure of each of the four conditions is shown in Fig. 3. Subplot 3a shows the microstructure of the Ti6Al4V processed below the beta transus temperature, with a bimodal microstructure shown made up of equiaxed primary alpha grains and a course transformed beta structure, owing to the relatively slow cooling rate during the FAST process. In comparison, subplot 3b shows the microstructure of the Ti6Al4V processed above the beta transus temperature, here a lamellar microstructure can be seen, made up of similarly-oriented alpha colonies separated by retained beta, with alpha along the prior beta grain boundaries. Subplots 3c and d show the Ti5Al5V5Mo3Cr at the two different processing temperatures, a similar microstructure is shown in both cases with larger grains in the higher temperature processed sample (as anticipated in previous paragraph).

**Fig. 4:**
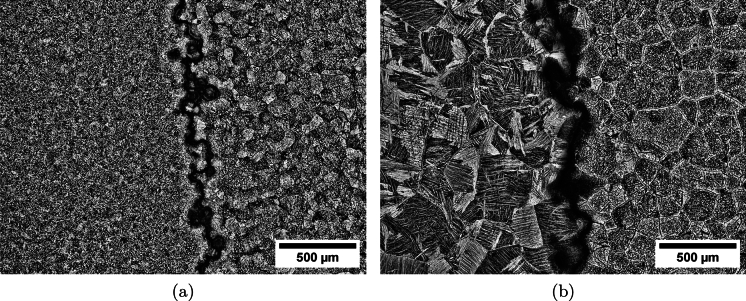
Light optical micrographs of difussion bond of multimaterial FAST-manufactured titanium alloys: **a** Ti6Al4V-970 / Ti5Al5V5Mo3Cr-970 and **b** Ti6Al4V-1100 / Ti5Al5V5Mo3Cr-1100. The numbers 970 and 1100 correspond to the dwell temperature at which the alloy was processed

Figure 4a and b show two micrographs of the diffusion bond between Ti6Al4V-970 and Ti5Al5V5Mo3Cr-970, and between Ti6Al4V-1100 and Ti5Al5V5Mo3Cr-1100, respectively. Both micrographs show the irregular bond morphology running vertically in the centre. The uneven interface can be attributed to the powder particle morphology during the mould lay-up. In both cases, the diffusion bond region appears darker in colour than the bulk material, this is an etching effect related to the fine-scale microstructure in the bond region between Ti6Al4V and Ti5Al5V5Mo3Cr, as previously reported by Pope et al. (2019). The bond width is considerably greater in the sample processed at $1100^{\circ }\text {C}$ due to the enhanced levels of diffusion at this temperature. Motyka et al. (2020) reported similar fine microstructural features in the diffusion bond between conventional CP-Ti and Ti15V3Al3Cr3Sn material, however the Frenkel pore effect reported in their study was not observed in the current or previous FAST diffusion bonding studies.

### Impact testing setup

The experimental configurations utilized in this study mirror those employed in Nieto-Fuentes et al. (2023a) and Nieto-Fuentes et al. (2023b) for conducting fragmentation tests on cylinders and rings, respectively. Note that the testing methodology for ring-shaped samples builds upon the approach used for cylinders. A concise overview of the impact testing setup is presented here, while a more detailed description can be found in Nieto-Fuentes et al. (2023a, 2023b).

A single-stage helium-driven gas gun, located at the Impact Laboratory of the University Carlos III of Madrid, has been utilized to propel a conical-nose cylindrical projectile towards the specimen at velocities within two different ranges: $248~\text {m}/\text {s} \le v_{z} \le 267 ~\text {m}/\text {s}$ (range 1) and $354~\text {m}/\text {s} \le v_{z} \le 390 ~\text {m}/\text {s}$ (range 2). The bore diameter of the gas gun barrel is $25~\text {mm}$. The projectile—also referred to as striker throughout this manuscript—is machined from a hardened alloy steel bar to prevent deformation during impact. It measures $64~\text {mm}$ in length, with a cone angle of $20^{\circ }$ and a base diameter of $24~\text {mm}$—see Fig. 2 in Nieto-Fuentes et al. (2023a). Note that the diameter of the cylindrical part of the projectile is approximately twice as large as the inner diameter of the rings and cylinders tested, see Sect. 2.1. The estimated nominal circumferential strain rate on the specimen is ${{\dot{\varepsilon }}}_{\theta }=\frac{v_r}{R_{m}}$, where $v_r$ denotes the radial expansion velocity, and $R_{m}$ is the radius of the specimen at half thickness. The radial expansion velocity, given by $v_r=\frac{v_z \sin (2\alpha )}{2}$, where $\alpha =70^{\circ }$ is the complement to the cone angle (see Fig. 2 in Nieto-Fuentes et al. (2023a)), is computed under the assumption that the specimen moves perpendicular to the conical nose of the projectile. A sabot, made of PLA and measuring $24.8~\text {mm}$ in diameter and $100~\text {mm}$ in length, is inserted into a pin machined at the base of the projectile—refer to Fig. 2 in Nieto-Fuentes et al. (2023a)—to stabilize the striker’s motion within the gun barrel and achieve axial impact on the specimen. The combined mass of the projectile-sabot assembly is $157~\text {g}$.

**Fig. 5:**
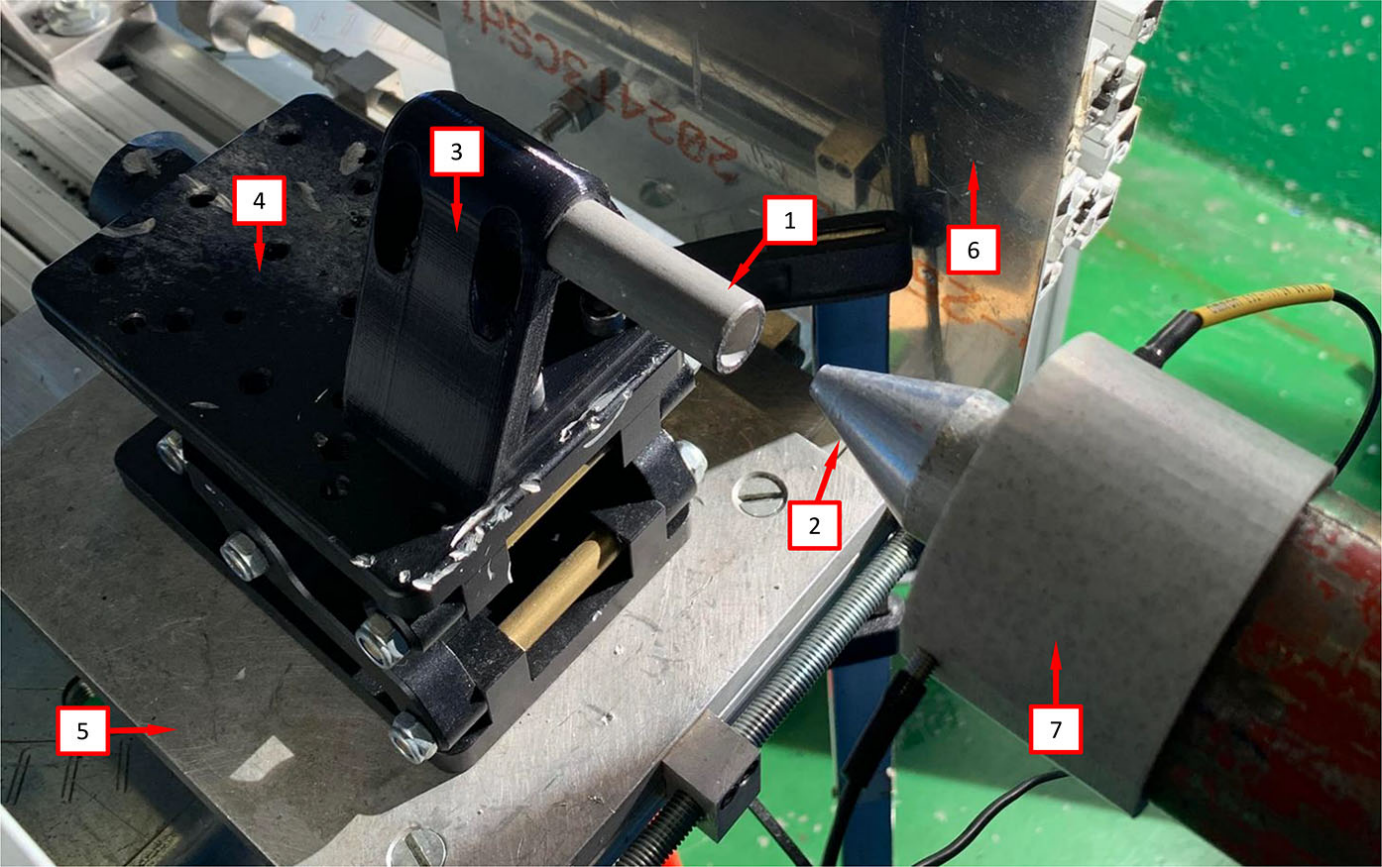
Experimental setup. Cylinder-shape specimens: (1) sample, (2) conical-nosed cylindrical projectile, (3) printed PLA pyramidal support, (4) height regulator jack, (5) XY precision table, (6) tunnel-shaped aluminum casing (the polymer foam has been removed to lighten the picture) and (7) gun barrel

**Fig. 6:**
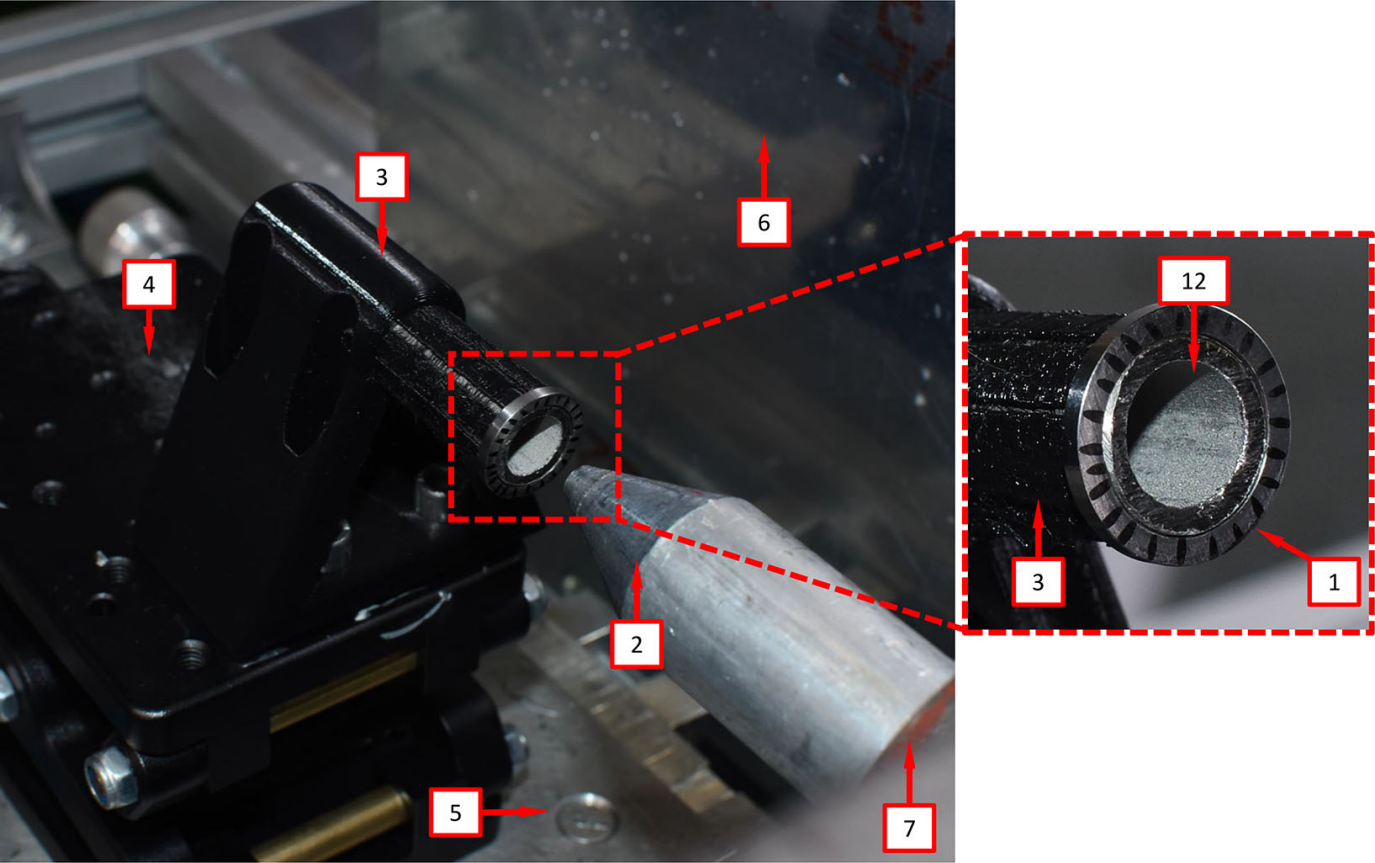
Experimental setup. Ring-shape specimens: (1) sample (with a black striped pattern), (2) conical-nosed cylindrical projectile, (3) printed PLA pyramidal support, (4) height regulator jack, (5) XY precision table, (6) tunnel-shaped aluminum casing (the polymer foam has been removed to lighten the picture), (7) gun barrel and (12) pusher

**Fig. 7:**
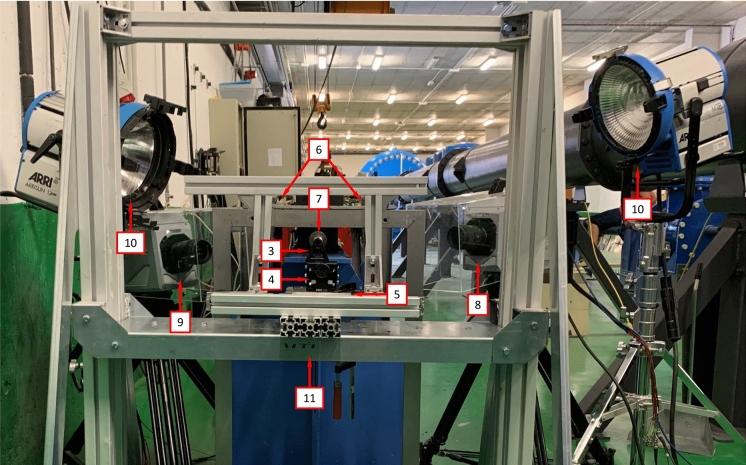
Experimental setup: (3) printed PLA pyramidal support, (4) height regulator jack, (5) XY precision table, (6) tunnel-shaped aluminum casing (the polymer foam has been removed to lighten the picture), (7) gun barrel, (8) high-speed camera 1, (9) high-speed camera 2, (10) lampheads and (11) aluminum structure screwed to the laboratory floor

- **Cylinder-shape specimens**. The sample is positioned with one end affixed to a printed PLA pyramidal support (referred to as the clamped end), while the opposite end is cantilevered (known as the impacted end)—see Fig. 5. The PLA support is situated on an XYZ assembly, incorporating an XY precision table and a height regulator jack. This setup enables the alignment of the cylindrical specimen’s axis with the gun barrel before conducting the tests. The XYZ assembly is attached to an aluminum structure that is fixed to the laboratory floor, see number 11 in Fig. 7. Refinements to the projectile trajectory are made by employing a laser that traverses both the gun barrel and the axis of the hollow cylinder before the experiment. Upon impact, the projectile axially penetrates the stationary cylinder-shape specimen, causing it to expand radially, triggering the formation and development of multiple cracks, ultimately resulting in the fragmentation of the specimen. Note that friction between the striker and the specimen may affect the fragmentation process. In future studies, experiments should involve lubricating the impacted end and the inner surface of the tube to evaluate whether the friction between the projectile and the cylinder influences the number of cracks and the size of the fragments.
- **Ring-shape specimens**. The specimen is inserted over a thin-walled tube made of $316\text {L}$ steel which is impacted axially by the conical-nose cylindrical projectile—see Fig. 6. The difference with respect to the setup used for the fragmentation of cylinder-shape specimens lies in the role of the steel tube, which serves as a pusher (the steel tube will be also referred to as the pusher throughout this manuscript), propelling the ring radially outward, causing it to break into multiple fragments. The 316 L steel is more ductile than the tested titanium alloys, leading to the failure of the pusher occurring after the fragmentation of the ring-shaped specimens. Moreover, a thin layer of grease is applied between the ring and the pusher to minimize friction effects during the tests. Note that the PLA support, into which the pusher is inserted, features 8 thin petals embracing the steel tube and extending to the rear of the specimen, preventing the airflow generated by the gas-gun to displace the ring before the projectile impacts the pusher—see Fig. 6.

The impact tests were recorded with two high-speed cameras Photron Fastcam SA-Z $2100~\text {K}$ using frame rates of $200000~\text {fps}$ and $350000~\text {fps}$ for the experiments on tubes and rings, respectively, resolutions of $256~\text {px}\times 232~\text {px}$ and $128~\text {px}\times 152~\text {px}$ for the tests on tubes and rings, respectively, and a shutter speed of $1/2880000~\text {s}$, see numbers 8 and 9 in Fig. 7. Two 1800-W open-face lampheads were utilized to provide sufficient lighting for capturing clear images, see number 10 in Fig. 7. The video recordings provide real-time insights into the localization and fracture mechanisms of rings and cylinders, as well as the number and location of necks and fractures formed. The recordings are also used to calculate the impact velocity by establishing a reference length prior to the test and determining the time it took for the projectile to cover this distance in the video footage. Furthermore, the recordings enabled us to verify whether the projectile impacted the target uniformly. The experiments in which the striker trajectory deviated more than $0.2~\text {mm}$ from the specimen axis were discarded (Nieto-Fuentes et al. 2023a). The fragments were soft-recovered using a tunnel-shaped casing lined with polymer foam placed around the specimen, see number 6 in Figs. 5, 6 and 7. The collected fragments were photographed, measured, and weighed to determine the spacing between necks and compile statistics on the distribution of fragment sizes—see Sects. 3 and 4. Additionally, selected fragments were subjected to fractography analysis using a FEI Inspect F FEG-SEM system in secondary electron mode with an accelerating voltage of $5~\text {kV}$, located at the Sorby Centre for Electron Microscopy at the University of Sheffield. The aim is to gain insights into the effect of impact velocity and materials selection on the dynamic localization and fragmentation behavior of FAST-manufactured titanium samples.

## Cylinder expansion tests

The impact fragmentation campaign for cylinder-shaped specimens comprised a total of 29 tests. Table 1 presents detailed information on each experiment: specimen designation, axial impact velocity ($v_z$), estimated circumferential strain rate (${\dot{\varepsilon }}_{\theta }$), average fragments length measured in the circumferential direction of the specimen along with its standard deviation (${\overline{L}}_{\theta } \pm$ SD), average fragments length measured in the axial direction of the specimen along with its standard deviation (${\overline{L}}_{z} \pm$ SD), average fragments thickness along with its standard deviation (${\overline{t}} \pm$ SD), average fragments mass along with its standard deviation (${\overline{m}} \pm$ SD), number of fragments recovered ($N_r$), and percentage of mass recovered relative to the complete cylinder (${{\widehat{m}}}$). The dimensions and mass of all recovered fragments are provided in Tables 8 to 36 of Appendix A. The circumferential length, axial length and thickness of each fragment were measured at three different locations using a digital caliper with a resolution of $0.01~\text {mm}$. The mean values of these three readings correspond to $L_{\theta }$, $L_{z}$, and *t* in the data sets of Appendix A. Note that the circumferential and axial length measurements account for the curvature of the fragments (i.e., the length was determined by accounting for the curvature of the fragments, rather than measuring along a straight line). Averaging the values of $L_{\theta }$, $L_{z}$, and *t* for all fragments recovered from each sample yields the average fragments length along the circumferential direction, ${\overline{L}}_{\theta }$, the average fragments length along the axial direction, ${\overline{L}}_{z}$, and the average fragments thickness, ${\overline{t}}$, as included in Table 1. We performed finite element calculations of the cylinder expansion tests, which indicated that the stress state in the specimen close to the impact zone is nearly uniaxial tension prior to neck formation. However, the results of these calculations are not shown here for the sake of brevity. The tests on monolithic and multimaterial samples are presented in Sects. 3.1 and 3.2, respectively. The notation used for the specimens designation is as follows: C refers to cylinder, Ti6Al4V and Ti5Al5V5Mo3Cr indicate the titanium alloy grades used to produce the specimens, and 970 and 1100 are the dwell temperatures at which the FAST process was conducted (as mentioned in Sect. 2.1). Moreover, S and D determine whether the specimen is manufactured using one or two titanium alloy grades, and PP and PL define, in the case of specimens produced with two grades, whether the diffusion bond between the two different grades was placed perpendicular or parallel to the axis of the cylinder (see Sect. 2.1). The specimens manufactured with a single titanium grade will be referred to as monolithic along this manuscript, while those produced with two titanium grades will be referred to as multimaterial (as mentioned in Sect. 2.1). The last digit in the specimens designation, ranging from 1 to 6, identifies the test number for the same material(s) system. Missing numbers correspond to failed tests that have been discarded due to the nonuniform impact of the striker on the inner circumference of the cylinder, see Sect. 2.2.

**Table 1:** The impact fragmentation campaign on cylinder-shape specimens consists of 29 experiments: specimen designation (see Sect. 2.1), axial impact velocity ($v_z$), estimated circumferential strain rate (${\dot{\varepsilon }}_{\theta }$), average fragments length measured in the circumferential direction of the specimen along with its standard deviation (${\overline{L}}_{\theta } \pm$ SD), average fragments length measured in the axial direction of the specimen along with its standard deviation (${\overline{L}}_{z} \pm$ SD), average fragments thickness along with its standard deviation (${\overline{t}} \pm$ SD), average fragments mass along with its standard deviation (${\overline{m}} \pm$ SD), number of fragments recovered ($N_r$), and percentage of mass recovered relative to the complete cylinder (${{\widehat{m}}}$)

*Specimen*	$v_{z}$ (m/s)	${\dot{\varepsilon }}_{\theta }$ ($\text {s}^{-1}$)	${\overline{L}}_{\theta }$ ± SD (mm)		${\overline{L}}_{z}$ ± SD (mm)		${\overline{t}}$ ± SD (mm)		${\overline{m}}$ ± SD (mm)		$N_r$	${{\widehat{m}}}$ (%)
C-Ti6Al4V-970-S-2	267.6	13216.8	16.06	6.50	13.97	4.13	0.99	0.03	1.978	1.336	3	81.4
C-Ti6Al4V-970-S-4	355.3	17561.9	11.77	5.00	21.22	10.37	0.91	0.02	1.892	1.623	3	78.6
C-Ti6Al4V-970-S-5	362.7	17871.8	18.62	13.44	20.75	9.64	0.98	0.05	2.946	2.641	2	81.7
C-Ti6Al4V-970-S-6	370.1	18272.5	14.26	5.10	24.29	8.51	0.96	0.05	1.696	0.698	4	92.9
C-Ti6Al4V-1100-S-1	255.5	12589.1	10.01	1.20	27.27	6.55	1.01	0.02	1.355	0.490	5	93.5
C-Ti6Al4V-1100-S-2	261.5	12887.7	9.41	3.82	17.09	9.55	0.99	0.03	0.969	1.149	7	94.1
C-Ti6Al4V-1100-S-3	256.4	12640.1	10.87	1.92	14.96	4.46	1.00	0.03	1.042	0.464	5	72.1
C-Ti6Al4V-1100-S-4	364.5	17966.9	8.04	2.02	19.41	12.80	0.96	0.02	0.735	0.498	9	91.6
C-Ti6Al4V-1100-S-5	354.3	17458.5	9.52	3.11	18.04	5.84	0.96	0.02	0.889	0.394	7	86.4
C-Ti6Al4V-1100-S-6	360.7	17773.3	8.10	1.51	17.43	9.16	0.95	0.03	0.597	0.395	11	90.8
C-Ti5Al5V5Mo3Cr-970-S-1	255.5	12570.4	10.01	2.40	29.31	8.88	0.94	0.04	1.391	0.575	5	91.8
C-Ti5Al5V5Mo3Cr-970-S-2	261.5	12907.5	10.20	1.43	26.56	11.17	0.96	0.04	1.418	0.661	5	93.5
C-Ti5Al5V5Mo3Cr-970-S-3	259.1	12755.8	15.45	8.45	14.77	3.24	0.94	0.07	1.404	1.010	5	92.7
C-Ti5Al5V5Mo3Cr-970-S-4	374.5	18423.8	8.18	1.56	25.19	12.19	0.95	0.03	0.866	0.403	8	91.4
C-Ti5Al5V5Mo3Cr-970-S-5	368.8	18139.9	9.14	2.64	18.27	3.25	0.95	0.04	0.849	0.344	7	78.3
C-Ti5Al5V5Mo3Cr-970-S-6	356.6	17551.2	8.34	1.93	25.27	12.60	0.94	0.04	0.877	0.423	8	92.5
C-Ti6Al4V/Ti5Al5V5Mo3Cr-970-D-PP-1	261.3	12911.8	10.23	1.79	13.38	2.82	0.91	0.06	0.746	0.197	2	20.2
C-Ti6Al4V/Ti5Al5V5Mo3Cr-970-D-PP-2	261.4	12898.6	9.62	2.20	12.77	4.03	0.94	0.04	0.890	0.250	8	88.6
C-Ti6Al4V/Ti5Al5V5Mo3Cr-970-D-PP-3	354.5	17510.0	17.79	7.27	25.16	12.01	0.90	0.05	2.401	1.069	3	97.3
C-Ti6Al4V/Ti5Al5V5Mo3Cr-970-D-PP-4	255.0	12590.0	9.10	3.21	28.26	9.77	0.94	0.03	1.454	0.505	5	98.0
C-Ti6Al4V/Ti5Al5V5Mo3Cr-970-D-PP-6	374.2	18475.7	8.56	1.90	17.89	10.47	0.95	0.03	0.805	0.545	9	97.5
C-Ti6Al4V/Ti5Al5V5Mo3Cr-970-D-PL-1	362.6	17881.3	7.75	2.13	24.84	10.36	0.96	0.05	0.967	0.646	7	91.4
C-Ti6Al4V/Ti5Al5V5Mo3Cr-970-D-PL-2	254.7	12561.5	11.19	2.88	19.84	5.10	0.94	0.03	1.459	0.762	5	98.6
C-Ti6Al4V/Ti5Al5V5Mo3Cr-970-D-PL-3	248.8	12290.9	9.59	3.32	24.21	9.63	0.97	0.03	1.301	0.730	5	87.6
C-Ti6Al4V/Ti5Al5V5Mo3Cr-970-D-PL-4	389.8	19267.2	9.37	2.16	20.91	9.93	0.95	0.03	0.984	0.660	7	99.7
C-Ti6Al4V/Ti5Al5V5Mo3Cr-1100-D-PL-1	266.0	13090.2	8.49	1.45	25.51	9.88	0.94	0.04	0.990	0.141	4	53.4
C-Ti6Al4V/Ti5Al5V5Mo3Cr-1100-D-PL-2	387.5	19124.9	7.68	2.03	20.14	9.26	0.97	0.05	0.777	0.514	9	94.1
C-Ti6Al4V/Ti5Al5V5Mo3Cr-1100-D-PL-3	263.7	13020.5	9.10	3.73	20.93	10.78	0.96	0.02	0.875	0.754	8	94.7
C-Ti6Al4V/Ti5Al5V5Mo3Cr-1100-D-PL-4	382.1	18877.8	7.33	1.53	21.44	11.95	0.96	0.02	0.684	0.463	9	83.2

### Monolithic samples

**Fig. 8:**
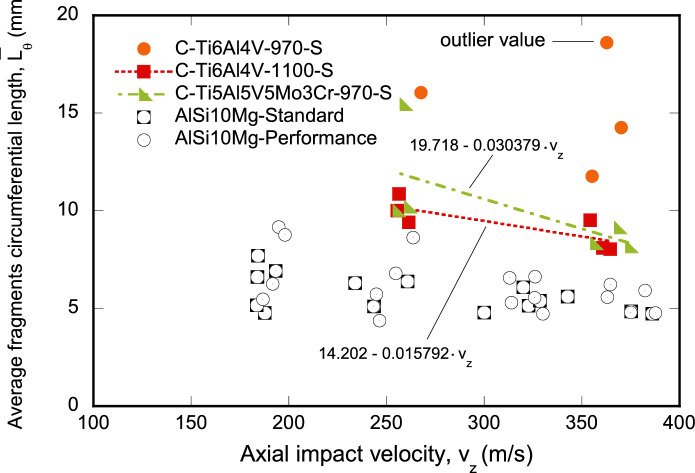
Variation of the average fragments circumferential length ${\bar{L}}_{\theta }$ with respect to the axial impact velocity $v_{z}$. Results corresponding to monolithic cylindrical specimens, see Table 1. Orange circles correspond to C-Ti6Al4V-970-S, red squares to C-Ti6Al4V-1100-S, and green triangles to C-Ti5Al5V5Mo3Cr-970-S. The experimental data for C-Ti6Al4V-1100-S and C-Ti5Al5V5Mo3Cr-970-S are fitted to straight lines. The results obtained with the monolithic titanium specimens fabricated with FAST are compared with tests reported by Nieto-Fuentes et al. (2023a) on additive manufactured AlSi10Mg samples. The black squares correspond to samples fabricated with standard quality and $\approx 6\%$ porosity, and the black circles to samples fabricated with performance quality and $\approx 2\%$ porosity. For interpretation of the references to color in this figure, the reader is referred to the web version of this article

Figure 8 shows the average fragments circumferential length ${\overline{L}}_{\theta }$ versus the axial impact velocity $v_{z}$ for the tests conducted on monolithic specimens: orange circles, red squares and green triangles correspond to C-Ti6Al4V-970-S, C-Ti6Al4V-1100-S, and C-Ti5Al5V5Mo3Cr-970-S, respectively. The experimental data for C-Ti6Al4V-1100-S and C-Ti5Al5V5Mo3Cr-970-S are fitted to straight lines, and the corresponding coefficients are provided in the figure legend. Despite the limited number of experiments for each material, the tests on C-Ti6Al4V-1100-S and C-Ti5Al5V5Mo3Cr-970-S specimens reveal a clear decrease in the fragments circumferential length with an increase in impact velocity. Based on the fitted curves, for an impact velocity of $250~\text {m}/\text {s}$, the average fragments circumferential length for C-Ti6Al4V-1100-S and C-Ti5Al5V5Mo3Cr-970-S is $10.25~\text {mm}$ and $12.20~\text {mm}$, respectively. For $370~\text {m}/\text {s}$, these values drop to $8.35~\text {mm}$ and $8.47~\text {mm}$. However, the results for C-Ti6Al4V-970-S do not exhibit a distinct trend concerning impact velocity, primarily due to the large value of ${\overline{L}}_{\theta }$ for $v_z=362.7~\text {m}/\text {s}$. Upon excluding this outlier, it seems like the average fragments circumferential length might be getting shorter as the impact speed increases, similar to what it is observed for C-Ti6Al4V-1100-S and C-Ti5Al5V5Mo3Cr-970-S. However, additional tests are required to ensure statistically significant results and establish clear patterns in how ${\overline{L}}_{\theta }$ varies with impact velocity for C-Ti6Al4V-970-S. On the other hand, a notable observation is the consistently larger fragments circumferential length for C-Ti6Al4V-970-S when compared to C-Ti6Al4V-1100-S and C-Ti5Al5V5Mo3Cr-970-S. Establishing a definitive correlation between the mechanical behavior of the three alloys and the size of the resulting fragments proves challenging. Nonetheless, these results suggest that the increased flow strength of C-Ti6Al4V-970-S compared to C-Ti6Al4V-1100-S and C-Ti5Al5V5Mo3Cr-970-S leads to a reduction in fragment count, see Fig. 3.

The results obtained with the monolithic titanium specimens fabricated with FAST are compared with tests reported by Nieto-Fuentes et al. (2023a) on additive manufactured AlSi10Mg samples. The black squares correspond to samples fabricated with standard quality and $\approx 6\%$ porosity, and the black circles to samples fabricated with performance quality and $\approx 2\%$ porosity. The AlSi10Mg printed specimens exhibit a smaller value for the average fragment length than the titanium samples manufactured with FAST across the entire range of impact velocities tested. The likely reason is the significant presence of porosity defects in the additive manufactured AlSi10Mg cylinders (Nieto-Fuentes et al. 2023a), which promote the formation of cracks favoring multiple fragmentation. However, note that, while AlSi10Mg cylinders exhibit a higher susceptibility to fragmentation, the rate of decrease in average fragment length with impact velocity appears to be similar for both printed AlSi10Mg and FAST titanium samples.

**Fig. 9:**
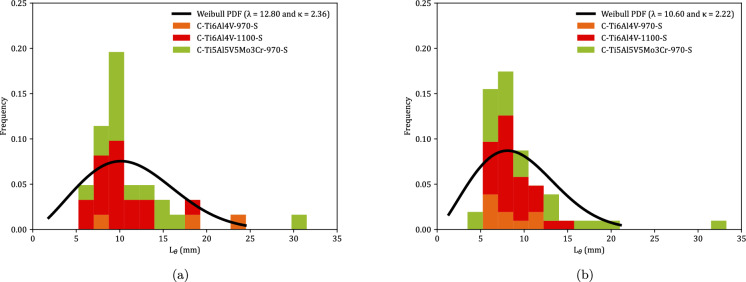
Distributions of fragments circumferential length $L_{\theta }$ corresponding to monolithic cylindrical specimens, see Tables 8 to 23. Orange, red and green blocks correspond to C-Ti6Al4V-970-S, C-Ti6Al4V-1100-S, and C-Ti5Al5V5Mo3Cr-970-S samples, respectively. The results are collected as a function of the axial impact velocity: **a** range 1—$248~\text {m}/\text {s} \le v_z \le 267~\text {m}/\text {s}$ and **b** range 2—$354~\text {m}/\text {s} \le v_z \le 390~\text {m}/\text {s}$. A Weibull probability density function, see equation (1), was fitted to the experimental measurements (black solid line). For interpretation of the references to color in this figure, the reader is referred to the web version of this article

Figure 9 shows the distributions of fragments length $L_{\theta }$ corresponding to monolithic specimens, see Tables 8 to 23. Orange, red and green blocks correspond to Ti6Al4V-970, Ti6Al4V-1100, and Ti5Al5V5Mo3Cr-970, respectively (the same color coding used in Fig. 8). The height of a colored block within a bar of the histogram provides the number of fragments for a given interval of fragments length. The results are collected as a function of the impact velocity: (a) range 1—$248~\text {m}/\text {s} \le v_z \le 267~\text {m}/\text {s}$ and (b) range 2—$354~\text {m}/\text {s} \le v_z \le 390~\text {m}/\text {s}$. The presentation of data for grades Ti6Al4V-970, Ti6Al4V-1100, and Ti5Al5V5Mo3Cr-970 in the same graph ensures statistically significant results. However, since data from different materials processed at different dwell temperatures are utilized, the interpretation of Fig. 9 should be qualitative rather than quantitative (note that the experimental results presented in the paper do not provide definitive conclusions regarding the influence of material microstructure on the fragmentation mechanisms of titanium alloys produced via FAST). The range of fragment lengths narrows as the impact velocity increases, resulting in a reduction of both the mean $\left( \mu \right)$ and the standard deviation (SD) in the distribution of fragments—refer to Table 2 for details. The trend is consistent with the results obtained by Nieto-Fuentes et al. (2023a) with AlSi10Mg cylinders—see Fig. 24 therein. The decrease in fragment size with the loading rate might be explained by the theory of Mott (1947b), which suggests that as the loading rate increases, the release waves originating from initial fractures have less time to propagate, thereby limiting the extent of unloading and enabling the initiation of additional fractures at nearby locations. Mott (1947b) assumes that the fracture sites correspond to material points with low failure strain, which is attributed to the presence of defects (such as microvoids), statistical variations in material properties, and microstructure differences. Furthermore, the fragments length distributions have been fitted to a Weibull Probability Distribution Function (PDF) represented by a black solid line, see equation (1), with $\lambda$ and $\kappa$ being the scale and shape parameters, which are included in the upper-right part of Figs. 9a and b. The decrease in the scale parameter with increasing impact velocity is consistent with the ring expansion experiments performed by Zhang and Ravi-Chandar (2006) on aluminum 6061-O samples, illustrating a reduction in the distance between fractures as the loading rate rises. Moreover, note that the explosively driven fragmentation experiment conducted by Bolis et al. (2013) on a Ti6Al4V hemispherical shell at a strain rate of $17000~\text {s}^{-1}$ resulted in an average fragment size of $150~\text {mm}^2$ (no information was provided on the shell’s thickness or diameter). Assuming the fragments are approximately square, the average fragment length is $12.25~\text {mm}$. This value is similar to the average fragment lengths observed in the cylinder expansion tests reported in this study, see Table 2.

1 $$\begin{aligned} f = \frac{k}{\lambda } \left( \frac{L_\theta }{\lambda }\right) ^{k-1} e^{-(L_\theta /\lambda )^k} \end{aligned}$$

**Fig. 10:**
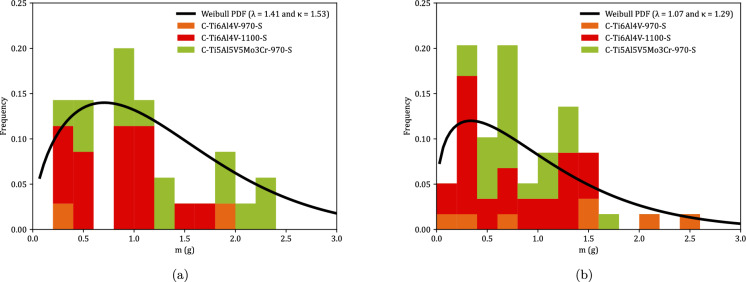
Distributions of fragments mass *m* corresponding to monolithic cylindrical specimens, see Tables 8 to 23. Orange, red and green blocks correspond to C-Ti6Al4V-970-S, C-Ti6Al4V-1100-S, and C-Ti5Al5V5Mo3Cr-970-S samples, respectively. The results are collected as a function of the axial impact velocity: (a) range 1—$248~\text {m}/\text {s} \le v_z \le 267~\text {m}/\text {s}$ and (b) range 2—$354~\text {m}/\text {s} \le v_z \le 390~\text {m}/\text {s}$. A Weibull probability density function, see equation (1), was fitted to the experimental measurements (black solid line). For interpretation of the references to color in this figure, the reader is referred to the web version of this article

Figure 10 illustrates the fragment mass distributions *m* for monolithic specimens, see Tables 8 to 23. The presentation of results is similar to that in Fig. 9, using the same color coding and displaying the data as a function of loading speed. Experiments for impact velocity ranges 1 and 2 are shown in subplots 10a and b, respectively. Both the mean ($\mu$) and standard deviation (SD) of the fragment mass distribution decrease with increasing impact velocity, see Table 3. The results have been fitted to a Weibull distribution function, indicated by the solid black line. The scale parameter of the distribution function decreases with increasing impact velocity, supporting the notion that the distance between fractures diminishes as the loading rate rises. The comparison of Figs. 9 and 10 reveals that whether assessing fragment length or mass, the results are qualitatively the same, indicating that the fragments are smaller with increasing impact velocity. Moreover, the thickness measurements of the fragments presented in Tables 8 to 23 demonstrate that the nominal strain at failure for the specimens tested is relatively low, with thickness reductions of less than $5\%$ in most cases. However, the variability in thickness measurements observed across experiments complicates the ability to draw definitive conclusions regarding potential differences in failure strain among the titanium alloys investigated.

**Table 2:** Mean ($\mu$) and standard deviation (SD) of the distribution of fragments length $L_{\theta }$ corresponding to monolithic cylindrical specimens, see Tables 8 to 23. Impact velocity range 1–$248~\text {m}/\text {s} \le v_z \le 267~\text {m}/\text {s}$. Impact velocity range 2–$354~\text {m}/\text {s} \le v_z \le 390~\text {m}/\text {s}$

	Impact velocity range 1	Impact velocity range 2
$\mu \; (\upmu \text {m})$	11.34	9.39
SD$\;(\upmu \text {m})$	4.94	4.33

**Table 3:** Mean ($\mu$) and standard deviation (SD) of the distribution of fragments mass *m* corresponding to monolithic cylindrical specimens, see Tables 8 to 23. Impact velocity range 1 – $248~\text {m}/\text {s} \le v_z \le 267~\text {m}/\text {s}$. Impact velocity range 2 – $354~\text {m}/\text {s} \le v_z \le 390~\text {m}/\text {s}$

	Impact velocity range 1	Impact velocity range 2
$\mu \; ({\text {g}})$	1.26	0.98
SD$\;(\text {g})$	0.89	0.88

**Fig. 11:**
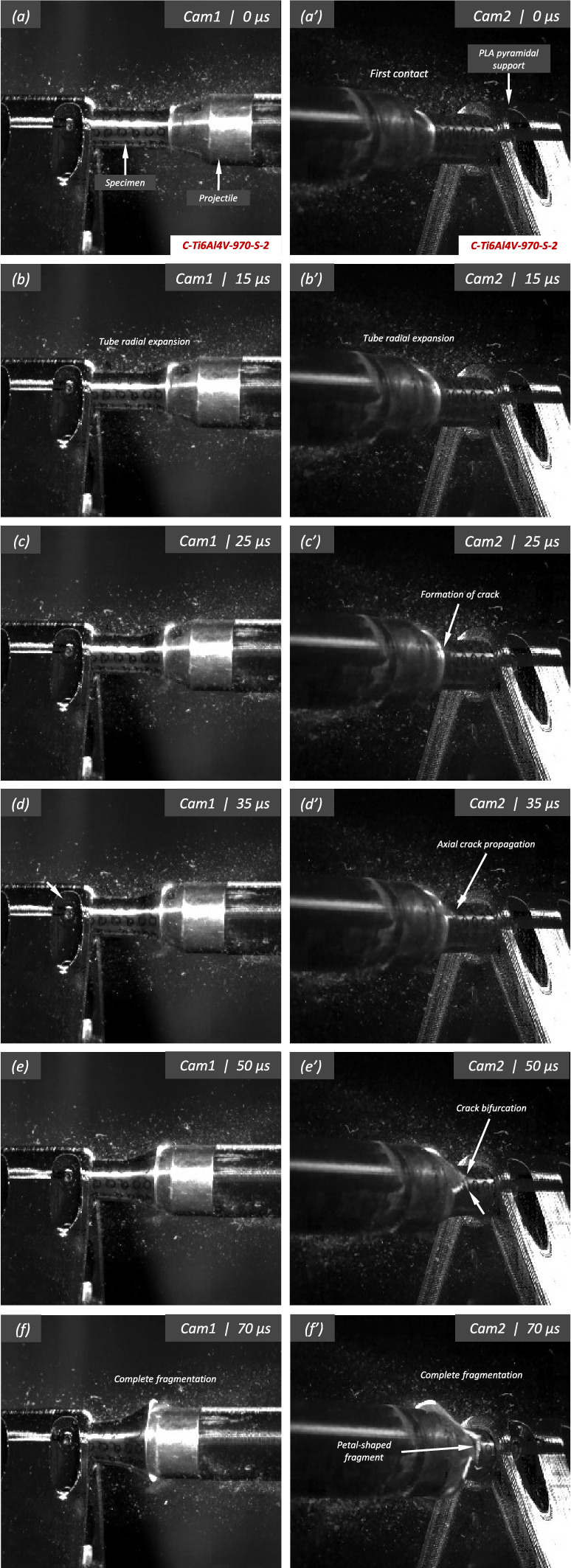
Sequence of images of the impact test for specimen C-Ti6Al4V-970-S-2 for different loading times: **a**–**a’** $\text {t}=0~\upmu \text {s}$, **b–b’** $\text {t}=15~\upmu \text {s}$, **c–c’** $\text {t}=25~\upmu \text {s}$, **d–d’** $\text {t}=35~\upmu \text {s}$, **e–e’** $\text {t}=50~\upmu \text {s}$ and **f–f’** $\text {t}=70~\upmu \text {s}$. Images obtained by camera 1 are on the left side of the sequence, while those obtained by camera 2 are on the right side. The impact velocity is $v_{z} = 267.6~\text {m}/\text {s}$ (impact velocity range 1). For interpretation of the references to color in this figure, the reader is referred to the web version of this article

**Fig. 12:**
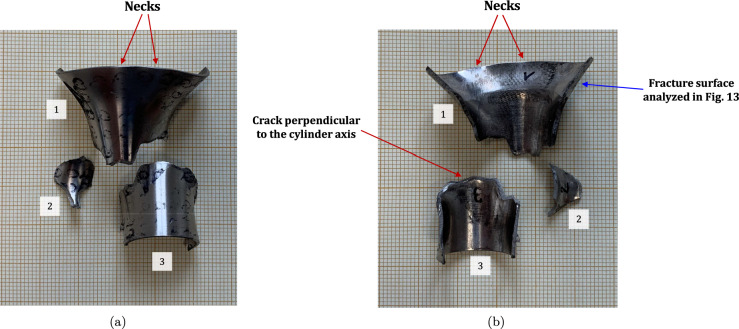
Post-mortem photography of the recovered fragments corresponding to specimen C-Ti6Al4V-970-S-2: **a** outer surface, **b** inner surface. The impact velocity is $v_{z} = 267.6~\text {m}/\text {s}$ (impact velocity range 1). The fragments are numbered the same as in Table 8 of Appendix A. Millimeter graph paper is used as a reference for the dimensions

**Fig. 13:**
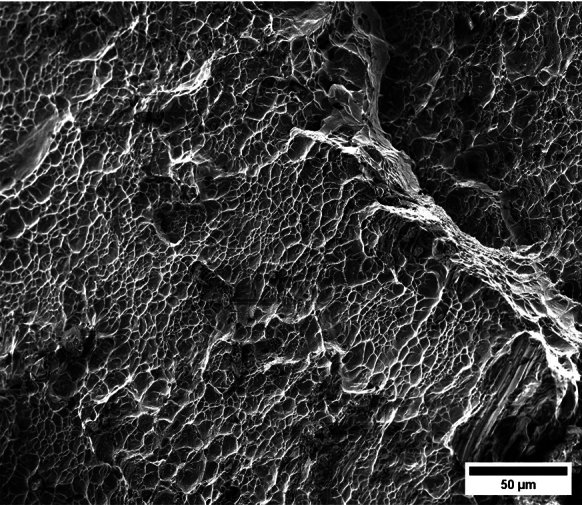
High-magnification SEM micrograph of fracture surface corresponding to fragment 1 of specimen C-Ti6Al4V-970-S-2 indicated in Fig. 12b with a blue arrow. The impact velocity is $v_{z} = 267.6~\text {m}/\text {s}$ (impact velocity range 1). For interpretation of the references to color in this figure caption, the reader is referred to the web version of this article

Figure 11 shows snapshots of the impact test on specimen C-Ti6Al4V-970-S-2 for different loading times. Recall that the sample was made of Ti6Al4V, and FAST processing was conducted at a dwell temperature of $970~^\circ \text {C}$, see Sect. 2.1 and Table 1 for details on the specimen processing and designation, respectively. The striker velocity is $v_{z} = 267.6~\text {m}/\text {s}$ (impact velocity range 1), and the estimated strain rate is $13216.8~\text {s}^{-1}$. This particular experiment has been selected to showcase the impact process due to the high-quality image recording and clear identification of the onset of fracture. The images obtained by camera 1 are on the left side of the sequence, while those obtained by camera 2 are on the right side. The first pair of images, snapshots (a)-(a’), corresponds to the time of impact $t = 0~\upmu \text {s}$. Observe the precise alignment between the conical nose of the projectile and the longitudinal axis of the cylinder. The uniform impact of the striker on the inner circumference of the cylinder is essential to ensure homogeneous deformation along the circumferential direction of the specimen. Images (b)-(b’) at $t=15~\upmu \text {s}$ showcase the radial expansion and axial bending of the cylinder, which develops a funnel-like shape as the projectile advances. The first fracture, indicated by a white arrow in snapshot (c’), forms at $t = 25~\upmu \text {s}$ and is preceded by a neck-like local thinning of the specimen thickness due to the circumferential stretching of the cylindrical casing. The crack initially extends in the axial direction of the specimen, as seen in (d’), and then bifurcates into two at $t=50~\upmu \text {s}$, as shown in (e’), eventually giving rise to a petal-shaped fragment indicated with a white arrow in image (f’). At $t=70~\upmu \text {s}$, the fragmentation process is complete. Subsequently, upon unloading the sample, the resulting fragments undergo radial expansion and (sometimes) bending during their free flight, most likely due to the sudden momentum change resulting from the violent fracture process. Postmortem photographs of the three recovered fragments are shown in Fig. 12 (only $81.4\%$ of the cylinder mass was recovered). The fragments are numbered the same as in Table 8 of Appendix A. The impacted end shows several necked sections pointed out with red arrows, which were formed during the expansion of the cylinder. As one moves away from the impacted end, fractures are not preceded by the formation of well-defined necks. Instead, cracks propagate at an oblique angle in relation to the circumferential direction of the ring. Note that slant fracture is commonly observed in Ti6Al4V sheets and shells subjected to high strain rates (Verleysen and Peirs 2017). The cracks are observed to initiate parallel to the projectile trajectory, then proceed to zigzag and twist (most likely due to the axial bending of the cylinder), ultimately resulting in the cylinder breaking perpendicular to the loading direction. Figure 13 shows a high-magnification SEM micrograph of the fracture surface indicated in Fig. 12b with a blue arrow. The fracture surface exhibits two distinct tiers, linked by a step indicating transient crack propagation perpendicular to the image, likely resulting from either the convergence of initially separate cracks or the bifurcation of an initial crack into branches. Notice the equiaxed dimples—flat dimple rupture (Barsoum and Faleskog 2007)—, characteristic of ductile tensile-dominated fracture (Tang et al. 2020) caused by the stretching of the material during the expansion and fragmentation of the cylinder. The larger dimples are likely caused by voids formed at prior beta grain boundaries and alpha/beta subgrain interfaces, while smaller dimples of varying sub-micron sizes may have formed at different stages of the deformation and damage process (Pineau et al. 2016).

**Fig. 14:**
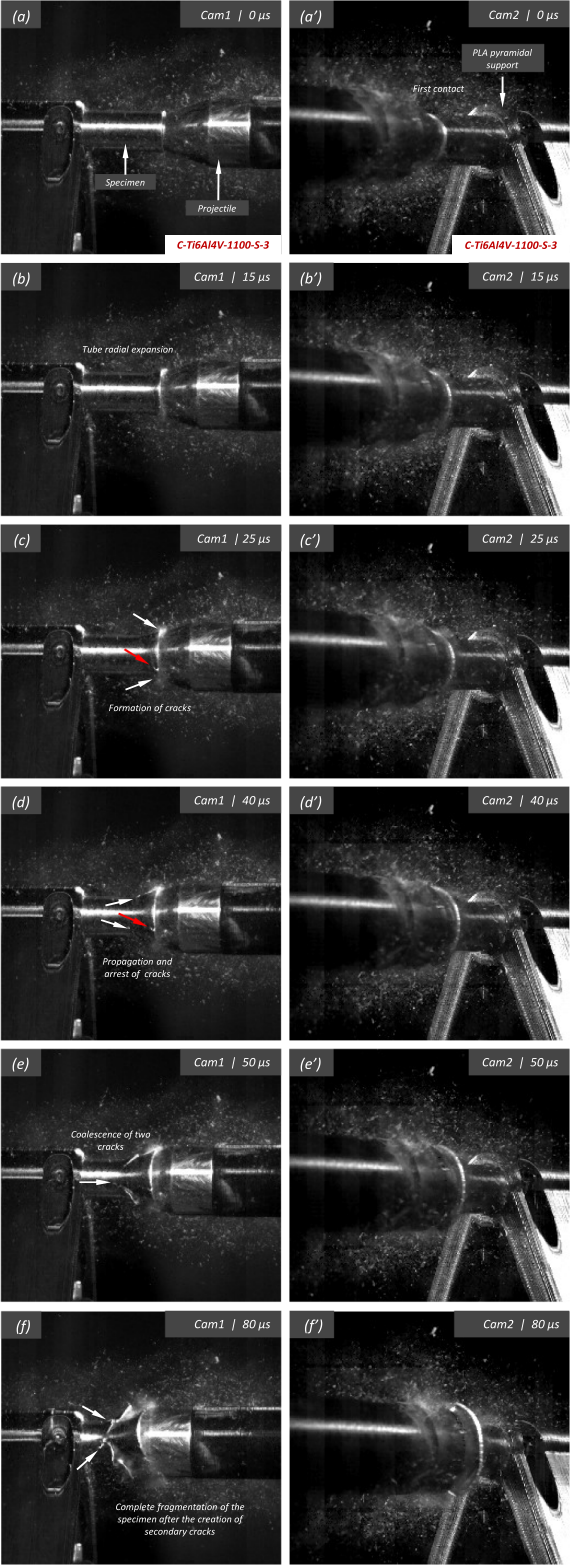
Sequence of images of the impact test for specimen C-Ti6Al4V-1100-S-3 for different loading times: **a**–**a’** $\text {t}=0~\upmu \text {s}$, **b**–**b’** $\text {t}=15~\upmu \text {s}$, **c**–**c’** $\text {t}=25~\upmu \text {s}$, **d**–**d’** $\text {t}=40~\upmu \text {s}$, **e**–**e’** $\text {t}=50~\upmu \text {s}$ and **f**–**f’** $\text {t}=80~\upmu \text {s}$. Images obtained by camera 1 are on the left side of the sequence, while those obtained by camera 2 are on the right side. The impact velocity is $v_{z} = 256.4~\text {m}/\text {s}$ (impact velocity range 1). For interpretation of the references to color in this figure, the reader is referred to the web version of this article

Figure 14 displays a series of snapshots corresponding to the impact test on specimen C-Ti6Al4V-1100-S-3. The material was FAST-processed at a dwell temperature of $1100~^{\circ }\text {C}$, differing from the $970~^{\circ }\text {C}$ used for specimen C-Ti6Al4V-970-S-2 depicted in Figs. 11, 12, 13. Recall that the effect of the dwell temperature on the stress–strain characteristic and microstructure of Ti6Al4V was discussed in Sect. 2.1. The striker velocity is $v_{z} = 256.4~\text {m}/\text {s}$ (impact velocity range 1), which is *only* $4\%$ less compared to specimen C-Ti6Al4V-970-S-2. The snapshots illustrate the impact process from the time at which the projectile hits the target until complete fragmentation of the cylindrical specimen. Images (a)-(a’) show the uniform contact of the striker on the inner circumference of the cylinder, while images (b)-(b’) depict the axial penetration of the striker into the specimen, resulting in radial expansion and axial bending of the cylinder wall. Several cracks are formed at the impacted end at $\text {t}=25~\upmu \text {s}$, as indicated in (c). The specific locations where the first cracks form are likely determined by the presence of material defects or by local perturbations in the stress field caused by the projectile impact. The white arrows in (c), (d), and (e) indicate fractures initially propagating toward the axial direction of the specimen due to circumferential stretching of the cylindrical casing but gradually twisting and eventually progressing at a certain inclination with respect to the specimen axis, likely due to bending of the cylinder wall. These two fractures ultimately meet at $\text {t}=80~\upmu \text {s}$ leading to the formation of a triangular-shape fragment (number 4 in Fig. 16 and Table 14). The red arrow in (c) and (d) highlights a crack that forms between the two indicated by white arrows. It progressed only a short distance before arrest, see Fig. 16a, likely due to unloading caused by the fast-propagating neighboring fractures, which prevent the specimen portion between them from continuing to deform. The five fragments recovered correspond to $72.1\%$ of the mass of the specimen and are shown in Fig. 16. Note that none of the fragments cover the entire length of the cylinder, and both fragments corresponding to the impacted and clamped ends tend to exhibit slant fracture and sharp corners due to the intersection of cracks diagonally traversing the specimen. Figure 18a shows a high-magnification SEM micrograph of the fracture surface indicated in Fig. 16b with a blue arrow. The fracture surface displays both equiaxial dimples (nearly spherical) and elongated shallow dimples (nearly elliptical) of largely varying sizes. This observation suggests that the material experienced a combination of tensile and shear stress states (Barsoum and Faleskog 2007), likely arising from the stretching and bending of the cylinder, which is instrumental for the twisting of the cracks during the fragmentation process, and the slanted fracture occurring along planes oriented at $\approx 45^\circ$ with respect to the circumferential direction of the cylinder. Moreover, fractography analysis of both C-Ti6Al4V-970-S-2 and C-Ti6Al4V-1100-S-3 samples, as depicted in Figs. 13 and 18a, reveals evident signs of ductile fracture characterized by extensive plastic deformation. This underscores the ability of FAST-processed Ti6Al4V alloys to absorb energy under impact loading.

**Fig. 15:**
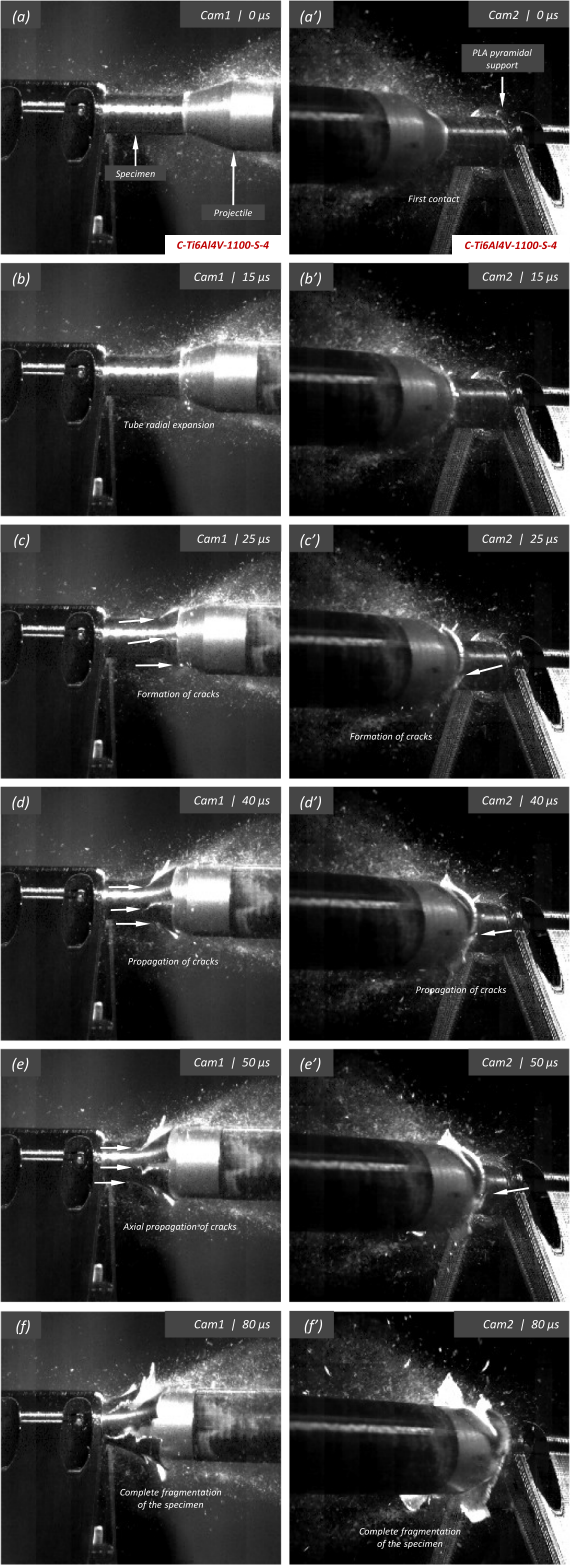
Sequence of images of the impact test for specimen C-Ti6Al4V-1100-S-4 for different loading times: **a**–**a’** $\text {t}=0~\upmu \text {s}$, **b–b’** $\text {t}=15~\upmu \text {s}$, **c–c’** $\text {t}=25~\upmu \text {s}$, **d–d’** $\text {t}=40~\upmu \text {s}$, **e–e’** $\text {t}=50~\upmu \text {s}$ and **f–f’** $\text {t}=80~\upmu \text {s}$. Images obtained by camera 1 are on the left side of the sequence, while those obtained by camera 2 are on the right side. The impact velocity is $v_{z} = 364.5~\text {m}/\text {s}$ (impact velocity range 2). For interpretation of the references to color in this figure, the reader is referred to the web version of this article

**Fig. 16:**
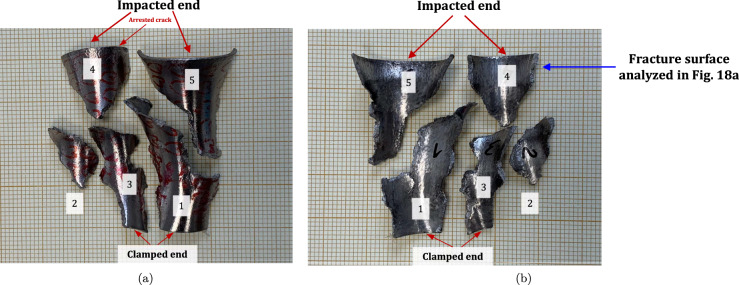
Post-mortem photography of the recovered fragments corresponding to specimen C-Ti6Al4V-1100-S-3: (a) outer surface, (b) inner surface. The impact velocity is $v_{z} = 256.4~\text {m}/\text {s}$ (impact velocity range 1). The fragments are numbered the same as in Table 14 of Appendix A. Millimeter graph paper is used as a reference for the dimensions

**Fig. 17:**
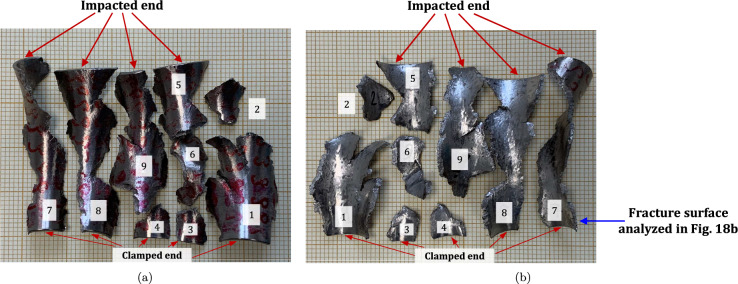
Post-mortem photography of the recovered fragments corresponding to specimen C-Ti6Al4V-1100-S-4: (a) outer surface, (b) inner surface. The impact velocity is $v_{z} = 364.5~\text {m}/\text {s}$ (impact velocity range 2). The fragments are numbered the same as in Table 15 of Appendix A. Millimeter graph paper is used as a reference for the dimensions

**Fig. 18:**
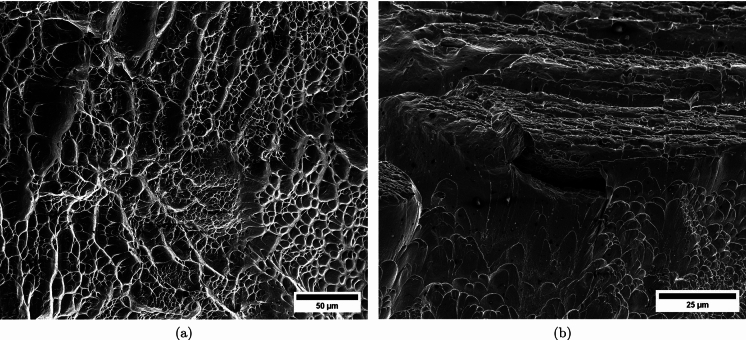
High-magnification SEM micrographs of fracture surfaces corresponding to: **a** fragment 4 of specimen C-Ti6Al4V-1100-S-3 indicated in Fig. 16b with a blue arrow and **b** fragment 7 of specimen C-Ti6Al4V-1100-S-4 indicated in Fig. 17b with a blue arrow. The samples C-Ti6Al4V-1100-S-3 and C-Ti6Al4V-1100-S-4 are tested at impact velocities of $256.4~\text {m}/\text {s}$ (impact velocity range 1) and $364.5~\text {m}/\text {s}$ (impact velocity range 2), respectively. For interpretation of the references to color in this figure caption, the reader is referred to the web version of this article

Figure 15 presents a sequence of snapshots recorded during the impact experiment on specimen C-Ti6Al4V-1100-S-4. The distinction from the test in Fig. 14 is the higher impact velocity: $v_{z} = 364.5~\text {m}/\text {s}$ (impact velocity range 2). The sequence of images captures the time at which the impact occurs (a)-(a’), followed by the uniform radial expansion of the specimen and axial bending of the cylinder wall (b)-(b’), the formation of fractures (c)-(c’), and their subsequent propagation (d)-(d’)-(e)-(e’), ultimately leading to the fragmentation of the sample (f)-(f’). In comparison to the test on sample C-Ti6Al4V-1100-S-3, an increase in impact velocity leads to the formation of a greater number of cracks. The fractures display a zigzag propagation pattern, where some appear to intersect, while others traverse the entire sample, resulting in longer and more regular fragments compared to the tests performed at lower speed (for projectile velocities within the impact speed range 1). An increase in impact velocity makes cracks less likely to twist and crisscross. This suggests that the axial bending of the cylinder wall diminishes with the loading rate, or is at least less pronounced compared to the radial expansion of the tube, which is responsible for the circumferential stretching of the cylindrical casing and the axial trajectory of the cracks. The same effect of impact velocity on cracks path was obtained in the tests performed by Nieto-Fuentes et al. (2023a) on printed AlSi10Mg cylinders. The nine fragments recovered from the test on sample C-Ti6Al4V-1100-S-4 are shown in Fig. 17. These fragments correspond to $91.6\%$ of the total mass of the cylinder. Fragments 7 and 8 encompass the entire length of the cylinder, see Table 15. Although the cracks shaping these two fragments followed a zigzag path (and the fragments are slightly bent since the specimen develops a funnel shape during the test), they did not intersect but instead traversed the cylinder from end to end, leading to the formation of slender strips with a *relatively* uniform cross-section. Fragment 9 is also notably long, covering more than three-fifths of the sample length,

see Table 15. While there are some short, triangle-shaped fragments (e.g., numbers 2, 3, and 4), a comparison with Fig. 16 reveals that an increase in impact speed tends to generate (some) longer fragments, which are also narrower due to the formation of more cracks at the impacted end of the sample (the size of Figs. 16 and 17 is practically the same, as observed on the millimeter paper, enabling a visual comparison of the fragment sizes in both experiments). Note that specimens C-Ti6Al4V-1100-S-5 and C-Ti6Al4V-1100-S-6, which were also tested within the impact velocity range 2, exhibited a similar fragmentation pattern with multiple slender fragments, see Tables 16 and 17. Note also that the impact tests conducted by Nieto-Fuentes et al. (2023a) on printed AlSi10Mg cylinders also showed that higher impact velocities lead to the formation of longer fragments. Figure 18b depicts a high-magnification SEM micrograph of the fracture surface of sample C-Ti6Al4V-1100-S-4, indicated by the blue arrow in Fig. 17b. The fractography analysis shows a multitude of flat sheared dimples, revealing a locally shear-dominated fracture (Liao and Duffy 1998; Xu et al. 2019). The formation of slant fractures, accompanied by elongated sheared dimples, is likely attributed to the sensitivity of Ti6Al4V to shear localization development at high loading rates (Da Silva and Ramesh 1997; Peirs et al. 2010). Upon reviewing the fractography analyses depicted in Figs. 13 and 18, it seems that distinct segments of the cracks demonstrate dominance either in tensile or shear loading, thereby revealing a complex stress state at the fracture level.

**Fig. 19:**
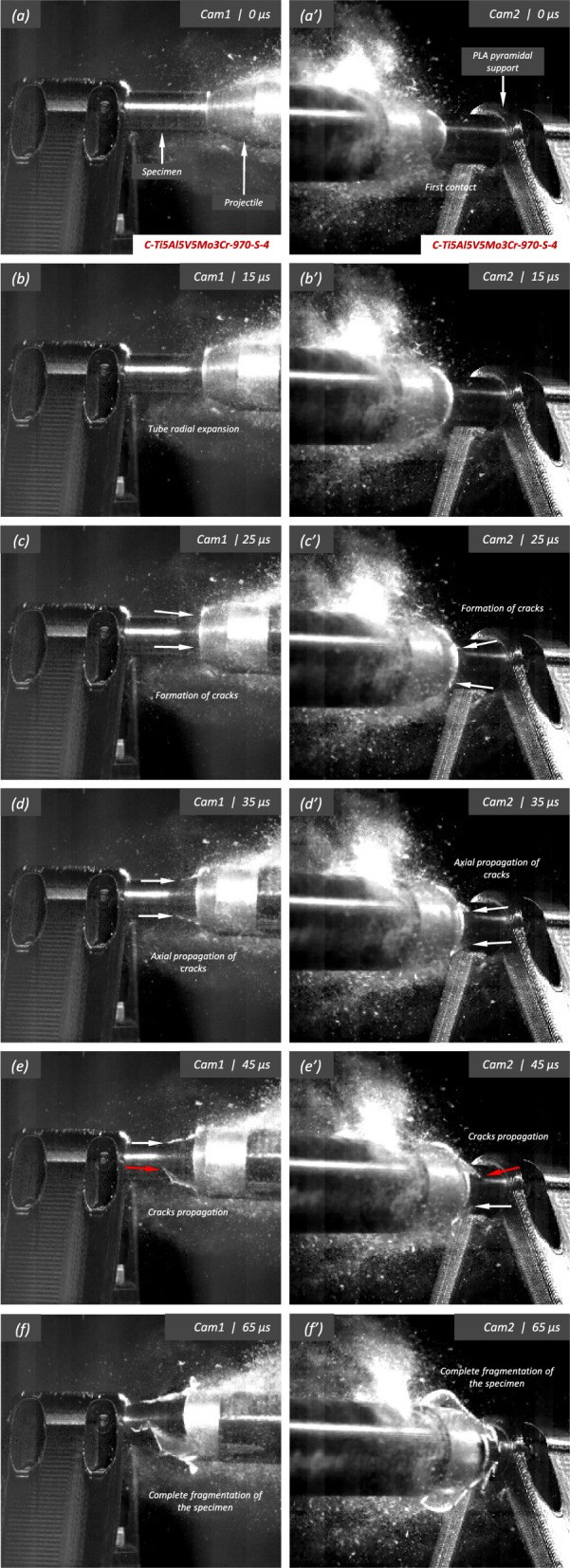
Sequence of images of the impact test for specimen C-Ti5Al5V5Mo3Cr-970-S-4 for different loading times: **a–a’** $\text {t}=0~\upmu \text {s}$, **b–b’** $\text {t}=15~\upmu \text {s}$, **c–c’** $\text {t}=25~\upmu \text {s}$, **d–d’** $\text {t}=35~\upmu \text {s}$, **e–e’** $\text {t}=45~\upmu \text {s}$ and **f–f’** $\text {t}=65~\upmu \text {s}$. Images obtained by camera 1 are on the left side of the sequence, while those obtained by camera 2 are on the right side. The impact velocity is $v_{z} = 374.5~\text {m}/\text {s}$ (impact velocity range 2). For interpretation of the references to color in this figure, the reader is referred to the web version of this article

**Fig. 20:**
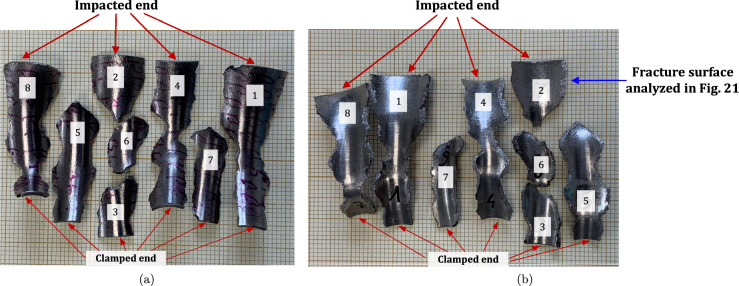
Post-mortem photography of the recovered fragments corresponding to specimen C-Ti5Al5V5Mo3Cr-970-S-4: **a** outer surface, **b** inner surface. The impact velocity is $v_{z} = 374.5~\text {m}/\text {s}$ (impact velocity range 2). The fragments are numbered the same as in Table 21 of Appendix A. Millimeter graph paper is used as a reference for the dimensions

**Fig. 21:**
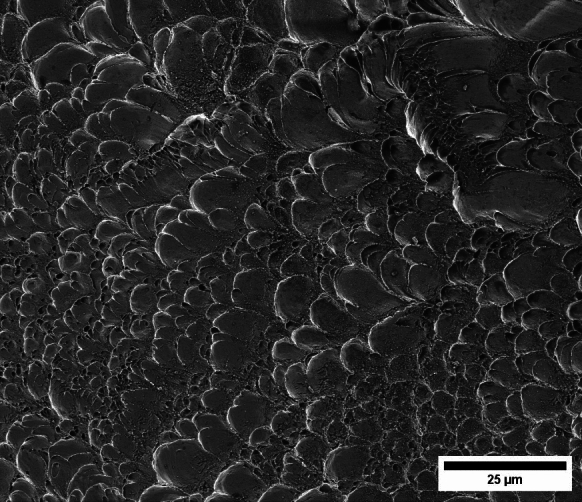
High-magnification SEM micrographs of fracture surface corresponding to fragment 2 of specimen C-Ti5Al5V5Mo3Cr-970-S-4 indicated in Fig. 20b with a blue arrow. The impact velocity is $v_{z} = 256.4~\text {m}/\text {s}$ (impact velocity range 1). For interpretation of the references to color in this figure caption, the reader is referred to the web version of this article

Figure 19 shows a series of images of the impact test on specimen C-Ti5Al5V5Mo3Cr-970-S-4 for different loading times. Note that the cylinder is made out of Ti5Al5V5Mo3Cr instead of the Ti6Al4V alloy used to produce the samples tested in the experiments shown in Figs. 11 to 18. Recall that the effect of the titanium alloy grade on the stress–strain characteristic and microstructure of the cylindrical specimens was discussed in Sect. 2.1. The impact velocity is $v_{z} = 374.5~\text {m}/\text {s}$ (impact velocity range 2). Notably, the fragmentation process closely resembles that observed in the experiment conducted on sample C-Ti6Al4V-1100-S-4, as analyzed in Figs. 15 to 17, where the impact velocity was (only) $3\%$ smaller. The cracks initiated at the impacted end of the cylinder propagate axially towards the clamped end, zigzagging and branching as they traverse the specimen. Several of these fractures intersect each other, leading to the formation of short fragments, while some other span the cylinder from end to end and lead to the formation of long, slender fragments (similar fragmentation process was observed for specimens C-Ti5Al5V5Mo3Cr-970-S-5 and C-Ti5Al5V5Mo3Cr-970-S-6 which were also tested within the impact velocity range 2). These findings suggest that the impact speed is a critical factor governing the fragmentation of titanium cylinders within the loading rates considered in this investigation. Despite the differences in material composition between specimens C-Ti6Al4V-1100-S-4 and C-Ti5Al5V5Mo3Cr-970-S-4, the observed fragmentation mechanisms show remarkable similarity, likely attributed to the fact that both samples were tested at similar speed. Figure 20 shows photographs of the eight fragments recovered from sample C-Ti5Al5V5Mo3Cr-970-S-4 which correspond to $91.4\%$ of the total mass of the cylinder. All fragments show slanted fracture surfaces. Fragments numbered 1, 4, and 8 cover the entire length of the specimen, while fragment 5 is three-fifths of the specimen’s length, see Table 21. The dimensions of Figs. 17 and 20 are nearly identical, as demonstrated by the millimeter paper, illustrating the comparable distributions of fragment sizes observed in both experiments. Figure 21 displays a secondary electron microscope image of the fracture surface referenced in Fig. 20b by a blue arrow. Observe the severely stretched dimples, elongated and displaying distinctive parabolic patterns. The material has flowed in the direction of maximum shear, resulting in a slant shear fracture inclined approximately $45^\circ$ relative to the circumferential direction of the specimen (Bron et al. 2004).

### Multimaterial samples

**Fig. 22:**
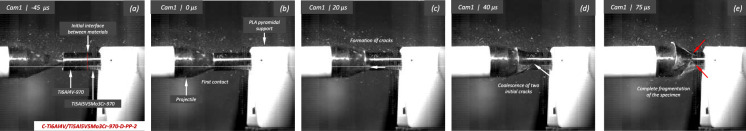
Sequence of images of the impact test for specimen C-Ti6Al4V/Ti5Al5V5Mo3Cr-970-D-PP-2 for different loading times: **a** $\text {t}=-45~\upmu \text {s}$, **b** $\text {t}=0~\upmu \text {s}$, **c** $\text {t}=20~\upmu \text {s}$, **d** $\text {t}=40~\upmu \text {s}$ and **e** $\text {t}=75~\upmu \text {s}$. Images obtained by camera 1. The impact velocity is $v_{z} = 261.4~\text {m}/\text {s}$ (impact velocity range 1). For interpretation of the references to color in this figure, the reader is referred to the web version of this article

**Fig. 23:**
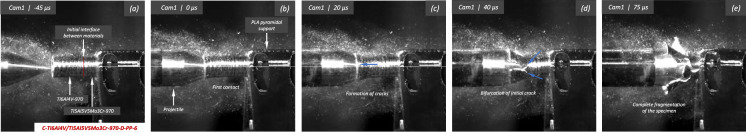
Sequence of images of the impact test for specimen C-Ti6Al4V/Ti5Al5V5Mo3Cr-970-D-PP-6 for different loading times: **a** $\text {t}=-45~\upmu \text {s}$, **b** $\text {t}=0~\upmu \text {s}$, **c** $\text {t}=20~\upmu \text {s}$, **d** $\text {t}=40~\upmu \text {s}$ and **e** $\text {t}=75~\upmu \text {s}$. Images obtained by camera 1. The impact velocity is $v_{z} = 374.2~\text {m}/\text {s}$ (impact velocity range 2). For interpretation of the references to color in this figure, the reader is referred to the web version of this article

**Fig. 24:**
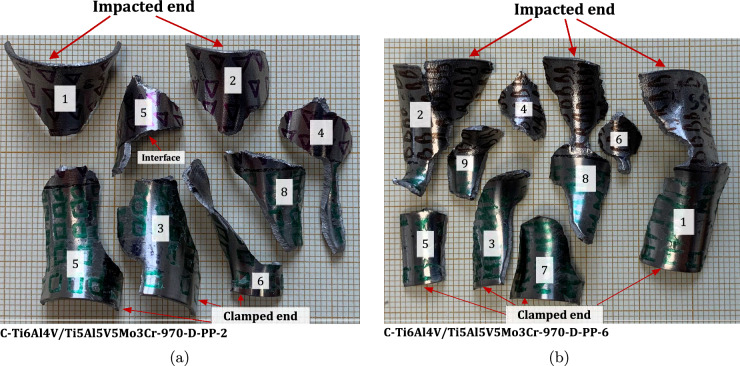
Post-mortem photography of the outer surface of recovered fragments corresponding to specimens: (a) C-Ti6Al4V/Ti5Al5V5Mo3Cr-970-D-PP-2 tested at $v_{z} = 261.4~\text {m}/\text {s}$ (impact velocity range 1) and (b) C-Ti6Al4V/Ti5Al5V5Mo3Cr-970-D-PP-6 tested at $v_{z} = 374.2~\text {m}/\text {s}$ (impact velocity range 2). The fragments are numbered the same as in Tables 25 and 28 of Appendix A. Millimeter graph paper is used as a reference for the dimensions

Figures 22 and 23 show the fragmentation process for specimens C-Ti6Al4V/Ti5Al5V5Mo3Cr-970-D-PP-2 and C-Ti6Al4V/Ti5Al5V5Mo3Cr-970-D-PP-6, respectively. The samples consist of two halves composed of Ti6Al4V-970 and Ti5Al5V5Mo3Cr-970, which divide the cylinder along a plane perpendicular to its axis, see Fig. 1 and Table 1. The distinction between these experiments is the impact velocity which is $261.4~\text {m}/\text {s}$ for C-Ti6Al4V/Ti5Al5V5Mo3Cr-970-D-PP-2 (velocity range 1) and $374.2~\text {m}/\text {s}$ for C-Ti6Al4V/Ti5Al5V5Mo3Cr-970-D-PP-6 (velocity range 2). The section made of Ti6Al4V is positioned on the impacted side of the specimens. The interface between the two materials is indicated with a red dashed line in snapshots 22a and 23a. The mechanisms governing crack formation and propagation closely resemble those observed in the experiments discussed in Sect. 3.1. The fractures initiated at the impacted end of the sample traverse the cylinder, exhibiting a distinct crisscross pattern. Some cracks interact, leading to the formation of short fragments, while others extend across the entire sample, forming long strips. Notably, the interface between the two materials exhibits no discernible impact on the propagation path of the fractures. The fragments recovered from these two experiments are included in Fig. 24. In the post-mortem photograph of specimen C-Ti6Al4V/Ti5Al5V5Mo3Cr-970-D-PP-2, the Ti6Al4V and Ti5Al5V5Mo3Cr sections are identified by motifs painted in purple and green, respectively, while in the case of sample C-Ti6Al4V/Ti5Al5V5Mo3Cr-970-D-PP-6, the two alloys are marked with black and green motifs. Note that it is only in fragment 5 of sample C-Ti6Al4V/Ti5Al5V5Mo3Cr-970-D-PP-2 that a crack (partially) propagates through the interface between the two materials. None of the other cracks forming the fragments extend along the boundary between the two specimen sections. Similar observations have been made for specimens C-Ti6Al4V/Ti5Al5V5Mo3Cr-970-D-PP-1, C-Ti6Al4V/Ti5Al5V5Mo3Cr-970-D-PP-3, and C-Ti6Al4V/Ti5Al5V5Mo3Cr-970-D-PP-4. However, the detailed results for these samples are not presented here to maintain brevity.

**Fig. 25:**
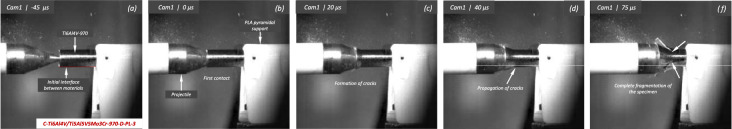
Sequence of images of the impact test for specimen C-Ti6Al4V/Ti5Al5V5Mo3Cr-970-D-PL-3 for different loading times: **a** $\text {t}=-45~\upmu \text {s}$, **b** $\text {t}=0~\upmu \text {s}$, **c** $\text {t}=20~\upmu \text {s}$, **d** $\text {t}=40~\upmu \text {s}$ and **e** $\text {t}=75~\upmu \text {s}$. Images obtained by camera 1. The impact velocity is $v_{z} = 261.4~\text {m}/\text {s}$ (impact velocity range 1). For interpretation of the references to color in this figure, the reader is referred to the web version of this article

**Fig. 26:**
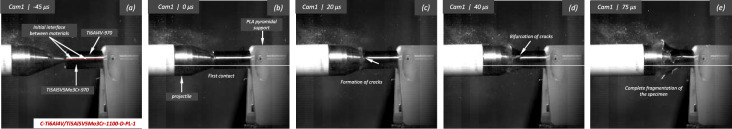
Sequence of images of the impact test for specimen C-Ti6Al4V/Ti5Al5V5Mo3Cr-1100-D-PL-1 for different loading times: **a–a’** $\text {t}=0~\upmu \text {s}$, **b–b’** $\text {t}=15~\upmu \text {s}$, **c–c’** $\text {t}=25~\upmu \text {s}$, **d–d’** $\text {t}=35~\upmu \text {s}$, **e–e’** $\text {t}=45~\upmu \text {s}$ and **f–f’** $\text {t}=65~\upmu \text {s}$. Images obtained by camera 1 are on the left side of the sequence, while those obtained by camera 2 are on the right side. The impact velocity is $v_{z} = 266.0~\text {m}/\text {s}$ (impact velocity range 1). For interpretation of the references to color in this figure, the reader is referred to the web version of this article

**Fig. 27:**
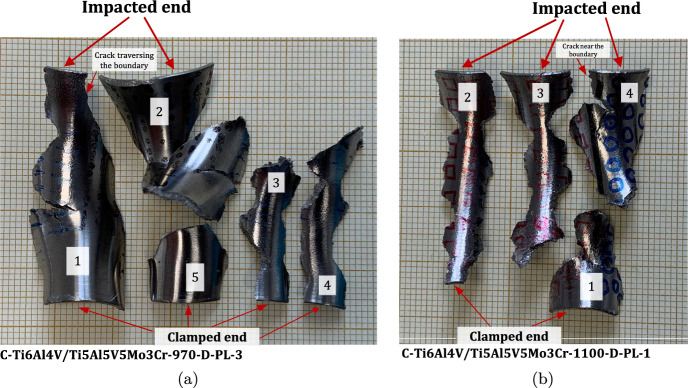
Post-mortem photography of the outer surface of recovered fragments corresponding to specimens: **a** C-Ti6Al4V/Ti5Al5V5Mo3Cr-970-D-PL-3 tested at $v_{z} = 261.4~\text {m}/\text {s}$ (impact velocity range 1) and **b** C-Ti6Al4V/Ti5Al5V5Mo3Cr-1100-D-PL-1 tested at $v_{z} = 266.0~\text {m}/\text {s}$ (impact velocity range 1). The fragments are numbered the same as in Tables 31 and 33 of Appendix A. Millimeter graph paper is used as a reference for the dimensions

Figures 25 and 26 showcase images captured during the fragmentation tests performed on specimens C-Ti6Al4V/Ti5Al5V5Mo3Cr-970-D-PL-3 and C-Ti6Al4V/Ti5Al5V5Mo3Cr-1100-D-PL-1, respectively. The samples consist of two halves composed of Ti6Al4V and Ti5Al5V5Mo3Cr which divide the cylinder along a plane parallel to its axis, see Fig. 1 and Table 1, so that the striker impacts both materials simultaneously. The interface between the two titanium alloy grades is indicated with a red dashed line in snapshots 25a and 26a. The projectile speed lies within the impact velocity range 1: $261.4~\text {m}/\text {s}$ in Fig. 25 and $266.0~\text {m}/\text {s}$ in Fig. 26. Note that the main distinction between these specimens is that the dwell temperature is $970^{\circ }$ for C-Ti6Al4V/Ti5Al5V5Mo3Cr-970-D-PL-3 and $1100^{\circ }$ for C-Ti6Al4V/Ti5Al5V5Mo3Cr-1100-D-PL-1. The video footage of Figs. 25 and 26 showcase that the interface has no noticeable effect on the specimens fragmentation; cracks neither preferentially form at the interface nor propagate along the materials boundary. Instead, the fractures exhibit the same zigzag pattern observed in all other specimens previously analyzed in Sects. 3.1 and 3.2, with some of these cracks intersecting as they propagate towards the clamped end of the cylinder. The fragments recovered from specimens C-Ti6Al4V/Ti5Al5V5Mo3Cr-970-D-PL-3 and C-Ti6Al4V/Ti5Al5V5Mo3Cr-1100-D-PL-1 are shown in Fig. 27a and b, respectively. Five fragments corresponding to $87.6\%$ of the total specimen mass were recuperated for C-Ti6Al4V/Ti5Al5V5Mo3Cr-970-D-PL-3. The Ti6Al4V and Ti5Al5V5Mo3Cr sections were colored with black dots and blue lines, respectively. The cracks do not propagate through the material interface; instead, they repeatedly traverse the boundary between the two halves of dissimilar materials. Similar observations are made based on the four fragments recovered for specimen C-Ti6Al4V/Ti5Al5V5Mo3Cr-1100-D-PL-1, which correspond to $53.4\%$ of the total cylinder mass. The Ti6Al4V and Ti5Al5V5Mo3Cr sections were colored with blue circles and purple squares, respectively. None of the cracks appears to progress through the material interface; and it is only in the case of fragment 4 that a fracture initiates near the boundary (albeit not directly at the boundary). These observations underscore that, in the tube expansion experiments conducted in this work, the fragmentation dynamics are largely independent of the material interface. Similar results have been obtained for all other multimaterial cylinders tested, although the video footage and fragments photographs are not presented for brevity.

## Ring expansion tests

The impact fragmentation campaign for ring-shaped specimens includes 27 tests. Table 4 presents detailed information on each experiment: specimen designation, axial impact velocity ($v_z$), estimated circumferential strain rate (${\dot{\varepsilon }}_{\theta }$), average fragments length measured in the circumferential direction of the specimen along with its standard deviation (${\overline{L}}_{\theta } \pm$ SD), average fragments width along with its standard deviation (${\overline{w}} \pm$ SD), average fragments thickness along with its standard deviation (${\overline{t}} \pm$ SD), average fragments mass along with its standard deviation (${\overline{m}} \pm$ SD), average necks spacing measured in the circumferential direction of the specimen along with its standard deviation (${\overline{L}}_{\theta }^{neck} \pm$ SD), number of fragments based on the video recordings ($N_v$), number of fragments recovered ($N_r$), number of necks in the fragments recovered including the necks that developed into fractures ($N_r^{neck}$), and percentage of mass recovered relative to the complete ring (${{\widehat{m}}}$). The dimensions and mass of all recovered fragments are provided in Tables 37 to 63, of Appendix B. The circumferential length, width and thickness of each fragment were measured following the same procedure applied to the cylinder-shape specimens, see Sect. 3. Despite recovering all visible fragments in some experiments, the mass of the fragments never matches the mass of the ring before testing. This discrepancy is likely attributed to the ejection of material particles and debris during crack formation and fragmentation. The criterion to consider that a neck is formed is that the square root of the quadratic sum of the engineering strains in both circumferential and axial directions at the necked section is greater than $5\%$. Note that the engineering strains are computed using the thickness and width of the specimen before and after testing. This necking criterion is arbitrary, yet we have checked that the number of necks considered is hardly sensitive to the cutoff value of strain chosen. Note that all the fractures originated at necked sections of the ring, see Sects. 4.1 and 4.2. The tests on monolithic and multimaterial samples are presented in Sects. 4.1 and 4.2, respectively. The notation used for designating the specimens is the same as that employed for cylinders, except the initial C has been replaced by an R, indicating a reference to rings.

**Table 4:**
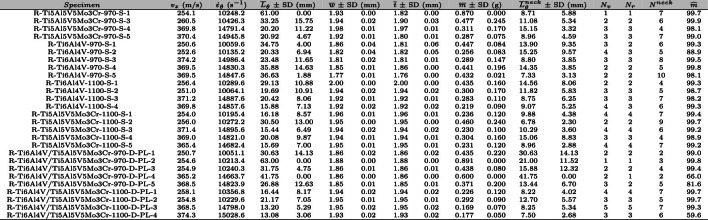
The impact fragmentation campaign on ring-shape specimens consists of 27 experiments: specimen designation (see Sect. 2.1), axial impact velocity ($v_z$), estimated circumferential strain rate (${\dot{\varepsilon }}_{\theta }$), average fragments length measured in the circumferential direction of the specimen along with its standard deviation (${\overline{L}}_{\theta }$ ± SD), average fragment width along with its standard deviation (${\overline{w}}$ ± SD), average fragments thickness along with its standard deviation (${\overline{t}}$ ± SD), average fragments mass along with its standard deviation (${\overline{m}}$ ± SD), average necks spacing measured in the circumferential direction of the specimen along with its standard deviation (${\overline{L}}_{\theta }^{neck}$ ± SD), number of fragments based on video recordings ($N_v$), number of fragments recovered ($N_r$), number of necks in the fragments recovered including the necks that developed into fractures ($N^{neck}$) and percentage of mass recovered relative to the complete ring (${\widehat{m}}$)

### Monolithic samples

**Fig. 28:**
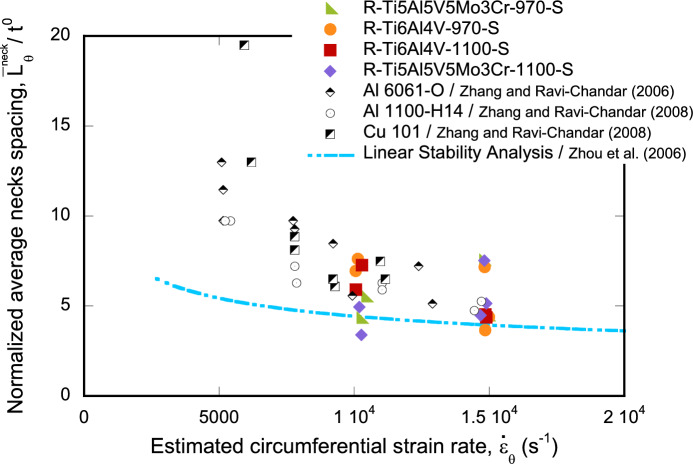
Variation of the normalized average necks spacing ${\overline{L}}^{neck}_{\theta } / t^{0}$ with respect to the estimated circumferential strain rate ${\dot{\varepsilon }}_{\theta }$. Results corresponding to monolithic ring specimens, see Table 4. Green triangles correspond to R-Ti5Al5V5Mo3Cr-970-S, orange circles to R-Ti6Al4V-970-S, red squares to R-Ti6Al4V-1100-S and purple diamonds to R-Ti5Al5V5Mo3Cr-1100-S. The results obtained with the monolithic titanium specimens fabricated with FAST are compared with ring expansion tests conducted by Zhang and Ravi-Chandar (2006, 2008) using an electromagnetic loading scheme on Al 60610-O, Al 1100-H14 and Cu 101 samples. For this comparison, the strain rate has been calculated by computing the ratio between the expansion velocity in Table 1 from Zhang and Ravi-Chandar (2006) and Tables 1 and 2 from Zhang and Ravi-Chandar (2008), and the mean radius of the specimens. The average neck spacing has been determined by calculating the ratio between the specimens’ perimeter before testing and the number of necks reported in Table 1 from Zhang and Ravi-Chandar (2006) and Tables 1 and 2 from Zhang and Ravi-Chandar (2008). The results obtained from the monolithic titanium specimens fabricated with FAST are also compared with the predictions of 1D linear stability analysis developed by Zhou et al. (2006). For interpretation of the references to color in this figure, the reader is referred to the web version of this article

Figure 28 shows the normalized average necks spacing ${\overline{L}}^{neck}_{\theta } / t^{0}$ versus the estimated circumferential strain rate ${\dot{\varepsilon }}_{\theta }$ for the tests conducted on monolithic specimens: green triangles, orange circles, red squares, and purple diamonds correspond to R-Ti5Al5V5Mo3Cr-970-S, R-Ti6Al4V-970-S, R-Ti6Al4V-1100-S and R-Ti5Al5V5Mo3Cr-1100-S, respectively. The results for the normalized average necks spacing vary between 3.4 and 7.6, with an average value of 5.5. While these experiments do not show definite trend regarding the effect of loading velocity on ${\overline{L}}^{neck}_{\theta } / t^{0}$ (more tests at different projectile velocities are needed), the average value of the necks spacing is slightly lower in the impact velocity range 2 than in range 1 (see the discussion below). Since data from different materials are utilized, this interpretation should be approached with caution (e.g., the results for R-Ti6Al4V-970-S are slightly higher than those for the other titanium alloys, see also Fig. 8). Nevertheless, the decrease in the distance between necks with the loading rate is consistent with experimental results obtained from ring expansion tests in the literature. For instance, Fig. 28 includes experimental data obtained from the tests conducted by Zhang and Ravi-Chandar (2006, 2008) using an electromagnetic loading scheme on Al 60610-O, Al 1100-H14 and Cu 101 rings (black and white diamonds, circles and squares). Note that normalizing the average neck spacing by the thickness enables a meaningful comparison of samples with different cross-sections (the samples tested in Zhang and Ravi-Chandar (2006, 2008) have a thickness of $0.5~\text {mm}$). The results obtained from the monolithic titanium specimens fabricated with FAST are quantitatively very close to the experimental data obtained by Zhang and Ravi-Chandar (2006, 2008) for the three materials they tested. The comparison with the experiments of Zhang and Ravi-Chandar (2006, 2008) suggests that the ring expansion tests conducted in this paper do not exhibit a clear trend regarding the effect of loading rate on reducing neck spacing of titanium alloys, likely due to the relatively narrow range of impact velocities investigated (within the same range of loading rates, the tests of Zhang and Ravi-Chandar (2006, 2008) also show a mild decrease of the neck spacing).

The experimental results have also been compared with the predictions of the 1D linear stability analysis developed by Zhou et al. (2006). The linear stability approach involves introducing a small perturbation to the fundamental analytical solution of the ring expansion problem at a specified loading time (Fressengeas and Molinari 1985, 1994). The perturbation grows for strains greater than the Considère criterion at a rate denoted as $\eta ^+$, within a finite number of modes which are assumed to define the range of neck spacings observed in the localization pattern (Vaz-Romero et al. 2017). The mode that exhibits the fastest growth rate, denoted as $\eta ^+_c$, is characterized by a perturbation wavenumber referred to as the critical wavenumber $\xi _c$, which is used to compute the average neck spacing ${\overline{L}}_{\theta }^{neck}=\dfrac{2\pi }{\xi _c}$. The critical wavenumber evolves with the sample straining during the ring expansion (under dynamic loading, necking localization occurs for strains greater than the Considère criterion due to inertia effects (Vaz-Romero et al. 2017)). Therefore, the comparison between experiments and linear stability analysis requires to define a criterion for the perturbation mode to turn into a necking mode (N’souglo et al. 2020, 2021). For that purpose, Dudzinski and Molinari (1991) introduced the concept of effective instability, which is based on the assumption that a localized neck is triggered when the cumulative instability index $I=\int ^t_{t_{\textrm{consid}{\grave{\textrm{e}}}\textrm{re}}} \eta ^+ dt$ reaches a critical value (Vaz-Romero et al. 2017; N’souglo et al. 2020), which is known as the critical cumulative instability index $I_c$. In this study, we set the critical value at $I_c =0.18$, calibrated to fit the experiments conducted on Ti6Al4V-970 samples tested within the higher velocity range (Vaz-Romero et al. 2017). In the linear stability analysis, the mechanical behavior of the material is considered to follow rate-independent von Mises plasticity with the yield stress evolution given by $\sigma _Y=\sigma _0 \left( 1 + \frac{\varepsilon ^p}{\varepsilon _0} \right) ^n$, where $\sigma _0=807.48~\text {MPa}$ is the reference yield stress, $\varepsilon _0=0.0051$ is the reference strain, and $n=0.060$ is the strain hardening exponent. The values of $\sigma _0$, $\varepsilon _0$, and *n* have been adjusted to fit the stress–strain characteristic of Ti6Al4V-970 shown in Fig. 2. The stability analysis predictions are represented in Fig. 28 by a light-blue solid line. The analytical results find satisfactory qualitative correlation with the experimental data obtained from the FAST-processed titanium samples, showing a mild decrease of the average necks spacing within the range of loading rates investigated in this work. Despite the analytical predictions presented here being only partially validated by the experiments, they can be further assessed through independent tests that will be published in future work on multiple necking in titanium alloys (this is work in progress). Moreover, the observed underestimation of the average neck spacing at lower strain rates may be attributed to decreased inertia effects, leading to a necking pattern that is less controlled by the development of specific unstable necking wavelengths and more influenced by the formation of necks in the weaker sections of the specimens, likely due to material defects or deviations from perfectly uniform radial loading (N’souglo et al. 2018).

**Table 5:** Mean ($\mu$) and standard deviation (SD) of the distribution of necks spacing $L_{\theta }^ {neck}$ corresponding to monolithic ring specimens, see Tables 37 to 54. Impact velocity range 1 – $248~\text {m}/\text {s} \le v_z \le 267~\text {m}/\text {s}$. Impact velocity range 2 – $354~\text {m}/\text {s} \le v_z \le 390~\text {m}/\text {s}$

	Impact velocity range 1	Impact velocity range 2
$\mu \; (\upmu \text {m})$	10.77	9.97
SD $\;(\upmu \text {m})$	6.88	5.29

**Fig. 29:**
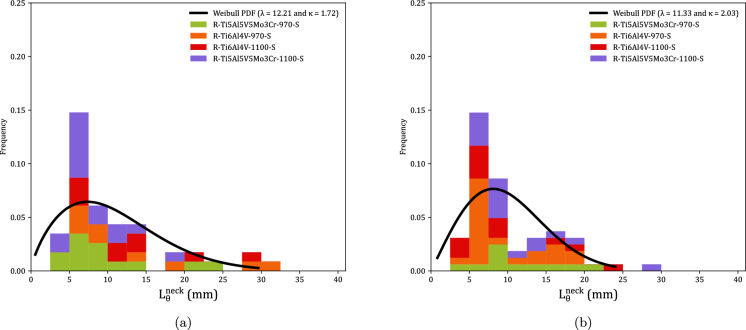
Distributions of necks spacing $L_{\theta }^{neck}$ corresponding to monolithic ring specimens, see Tables 37 to 54. Green, orange, red and purple blocks correspond to R-Ti5Al5V5Mo3Cr-970-S, R-Ti6Al4V-970-S, R-Ti6Al4V-1100-S and R-Ti5Al5V5Mo3Cr-1100-S samples, respectively. The results are collected as a function of the impact velocity: (a) range 1— $248~\text {m}/\text {s} \le v_z \le 267~\text {m}/\text {s}$ and (b) range 2—$354~\text {m}/\text {s} \le v_z \le 390~\text {m}/\text {s}$. A Weibull probability density function, see equation (1), was fitted to the experimental measurements (black solid line). For interpretation of the references to color in this figure, the reader is referred to the web version of this article

Figure 29 shows the distributions of necks spacing $L_{\theta }^{neck}$ corresponding to monolithic FAST-processed titanium ring specimens, see Table 4. Arrested necks and fractured necks are considered. Orange, red, green and purple blocks correspond to R-Ti5Al5V5Mo3Cr-970-S, R-Ti6Al4V-970-S, R-Ti6Al4V-1100-S and R-Ti5Al5V5Mo3Cr-1100-S samples, respectively. The results are collected as a function of the striker speed, with tests performed for impact velocity ranges 1 and 2 included in subplots 29a and b, respectively. Combining data for R-Ti5Al5V5Mo3Cr-970-S, R-Ti6Al4V-970-S, R-Ti6Al4V-1100-S, and R-Ti5Al5V5Mo3Cr-1100-S in a single graph enhances statistical significance of results, yet the interpretation of Fig. 29 should be qualitative due to the inclusion of data from two titanium grades processed at two different dwell temperatures. The range of necks spacing narrows as the impact velocity increases, leading to a (slight) reduction in both the mean ($\mu$) and the standard deviation (SD) in the distribution of neck-free segments, see Table 5. Furthermore, the necks spacing distributions have been fitted to a Weibull probability distribution function, see equation (1), represented in Fig. 29 by a black solid line. The numerical values of the scale and shape parameters are provided in the upper-right part of subplots 29a and b. Similar to the experiments conducted on cylinder-shaped specimens, the decrease in the scale parameter with the striker speed illustrates the reduction in the distance between necks as the loading rate increases. This finding might be rationalized using the theory proposed by Mott (1947b), suggesting that necks form at specific locations in the specimen due to statistical fluctuations in the critical necking strain resulting from material heterogeneity at the microstructural scale (Zhang and Ravi-Chandar 2006). The release wave emanating from necks that nucleate at a lower strain level is assumed to inhibit the nucleation of additional necks in nearby sections, so that the length of the neck-free segments is determined by the distance traveled by the unloading wave. Increasing the loading rate causes the release wave to cover a shorter distance, potentially explaining the decrease in neck spacing as the projectile speed increases (Zhang and Ravi-Chandar 2006). In addition, the stabilizing effect of inertia on long necking wavelengths may also contribute to the decrease in neck spacing with the loading rate. The stability analysis used to predict the experimental data in Fig. 28 states that inertia leads to a decrease in the average neck spacing as the loading rate increases (Mercier and Molinari 2003; Rodríguez-Martínez et al. 2013, 2017), favoring the formation of more necks nucleating at shorter distances due to the development of unstable necking modes. In this work, we assume that both defects and inertia interact to determine the final characteristics of the necking pattern in the ring expansion experiments, with their relative contributions dictated by the specific loading rate. The role of defects as preferential nucleation sites for necks, combined with the unloading waves generated by early necks, constitutes a mechanism of growing importance in controlling the spacing between adjacent necks as the strain rate decreases. Conversely, the effect of inertia, which promotes the growth of specific necking wavelengths that determine the spacing between consecutive necks, becomes more pronounced as the strain rate increases. When both defects and inertia contribute to the fragmentation of the rings, defects likely establish preferential sites for plastic localization, some of which evolve into necks, with spacing influenced by unstable wavelengths driven by inertia. Note that the inertia parameter ${\bar{H}}^{-1}$, as defined in equation $\left( 3.28\right)$ of N’souglo et al. (2018), has a value of approximately 0.025 for the experiments conducted in this study, while numerical simulations reported in N’souglo et al. (2018) indicated that the effect of defects on multiple necking formation is important for inertia parameter values below 0.1 (therefore, it appears plausible that both defects and inertia contribute to the formation of the multiple necking pattern observed in the experiments conducted in this study).

**Fig. 30:**
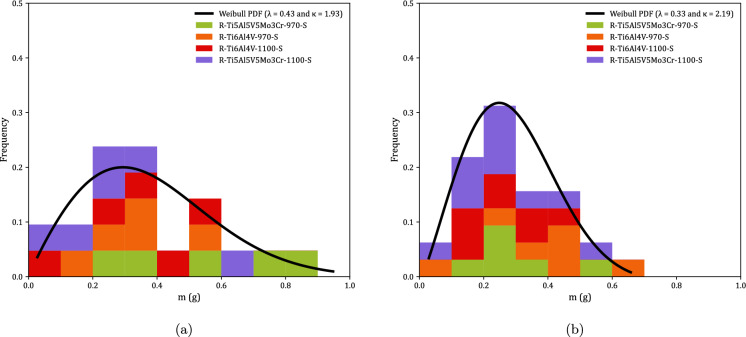
Distributions of fragments mass *m* corresponding to monolithic ring specimens, see Tables 37 to 54. Green, orange, red and purple blocks correspond to R-Ti5Al5V5Mo3Cr-970-S, R-Ti6Al4V-970-S, R-Ti6Al4V-1100-S and R-Ti5Al5V5Mo3Cr-1100-S samples, respectively. The results are collected as a function of the impact velocity: (a) range 1— $248~\text {m}/\text {s} \le v_z \le 267~\text {m}/\text {s}$ and (b) range 2—$354~\text {m}/\text {s} \le v_z \le 390~\text {m}/\text {s}$. A Weibull probability density function, see equation (1), was fitted to the experimental measurements (black solid line). For interpretation of the references to color in this figure, the reader is referred to the web version of this article

Figure 30 illustrates the fragment mass distributions *m* for monolithic specimens, see Tables 37 to 54. The results follow the format of Fig. 29, using the same color coding to differentiate the tested alloys and showing the data as a function of loading speed, with impact velocity ranges 1 and 2 illustrated in subplots 30a and b, respectively. The mass of the fragments decreases with increasing velocity, leading to a distribution that exhibits greater uniformity in fragment mass as the impact velocity rises. For instance, Table 6 shows that the mean fragment mass decreases from $0.38~\text {g}$ to $0.29~\text {g}$ as the loading speed increases from range 1 to range 2, while the standard deviation drops from $0.21~\text {g}$ to $0.14~\text {g}$. The experimental data has been fitted to a Weibull probability function, represented by the solid black line. The scale parameter, $\lambda$, decreases from 0.43 to 0.33 as the impact velocity increases from 1 to 2, consistent with experiments on cylinders, see Fig. 10, and illustrating a reduction in fracture spacing with higher loading rates. Note that the decrease in fragment size with increasing loading rate has been documented in the literature for most tested materials and appears to be independent of the material’s microstructure (Grady and Benson 1983; Zhang and Ravi-Chandar 2006; Nieto-Fuentes et al. 2023b). Moreover, the fragment sizes observed in the tests presented in this paper are consistent with experimental data reported in previous studies. For instance, Jones et al. (2012) conducted explosively driven ring fragmentation experiments on Ti6Al4V samples with thickness-to-length aspect ratios of 1 : 1, 1 : 2, and 1 : 4. The strain rate in the tests was approximately $10^4~\text {s}^{-1}$ (this value is similar to the experiments in this paper). The inner diameter and wall thickness of all specimens were $49~\text {mm}$ and $3~\text {mm}$, respectively. The samples of three different thickness-to-length aspect ratios tested by Jones et al. (2012) yielded average fragment sizes of $23.10~\text {mm}$, $12.82~\text {mm}$, and $18.17~\text {mm}$, respectively. These results are relatively similar to those obtained in this study, where the average fragment size for samples tested within the impact velocity ranges 1 and 2 is $30.60~\text {mm}$ and $22.46~\text {mm}$, respectively.

**Fig. 31:**
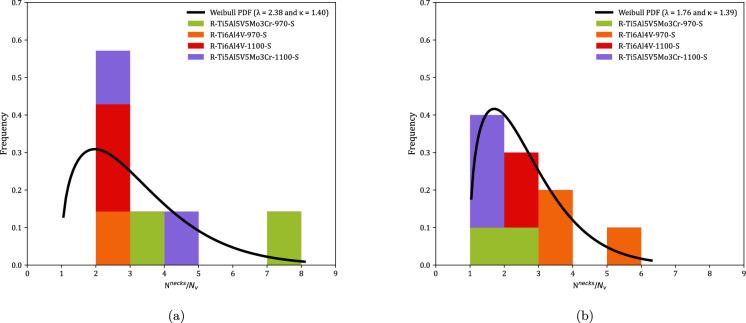
Distributions of neck-to-fragment ratio $N^{neck} / N_v$ corresponding to monolithic ring specimens, see Tables 37 to 54. Green, orange, red and purple blocks correspond to R-Ti5Al5V5Mo3Cr-970-S, R-Ti6Al4V-970-S, R-Ti6Al4V-1100-S and R-Ti5Al5V5Mo3Cr-1100-S samples, respectively. The results are collected as a function of the impact velocity: (a) range 1— $248~\text {m}/\text {s} \le v_z \le 267~\text {m}/\text {s}$ and (b) range 2—$354~\text {m}/\text {s} \le v_z \le 390~\text {m}/\text {s}$. A Weibull probability density function, see equation (1), was fitted to the experimental measurements (black solid line). For interpretation of the references to color in this figure, the reader is referred to the web version of this article

**Table 6:** Mean ($\mu$) and standard deviation (SD) of the distribution of fragments mass *m* corresponding to monolithic ring specimens, see Tables 37 to 54. Impact velocity range 1 – $248~\text {m}/\text {s} \le v_z \le 267~\text {m}/\text {s}$. Impact velocity range 2 – $354~\text {m}/\text {s} \le v_z \le 390~\text {m}/\text {s}$

	Impact velocity range 1	Impact velocity range 2
$\mu \; (\text {g})$	0.38	0.29
SD $\;(\text {g})$	0.21	0.14

Fig. 31 shows the distributions of neck-to-fragment ratio $N^{neck} / N_v$ corresponding to monolithic ring specimens. The data only consider experiments in which all fragments were recovered (${\hat{m}}>98\%$ in Table 4). The results for impact velocity ranges 1 and 2 are included in subplots 31a and b, respectively. The experimental data has been fitted to a Weibull probability density function. Increasing the loading speed leads to a narrower distribution, where the ratio $N^{neck} / N_v$ decreases toward 1, indicating that higher loading rates result in a greater proportion of necks developing into fractures. For the impact velocity range 1, the mean neck-to-fragment ratio is 3.14, while in the higher impact velocity range, this value drops to 2.10, see Table 7. The increased proportion of necks producing fractures at higher applied velocities is attributed to inertia effects, which promote uniform necks growth and fracture rates (Vaz-Romero et al. 2019). Additionally, increasing the applied velocity reduces the time for release waves from early necks and fractures to halt the development of adjacent necks before they break (Zhang and Ravi-Chandar 2006). It seems that, despite the microstructural differences between the tested titanium alloys, the distribution of neck and fragment sizes, as well as the evolution of the number of necks and fragments with the loading speed, can likely be analyzed using macromechanical theories based on the unstable growth of specific necking modes and the propagation of release waves from early necks and fractures. Further experimental investigations are likely needed to identify the specific effects of microstructure on the fragmentation characteristics of titanium alloys. Moreover, note that similar to the experiments on cylindrical samples, the thickness and width measurements of the ring fragments suggest that the nominal strain at failure is relatively low, with reductions in thickness and width being less than $10\%$ in most cases, see Tables 37 to 54. Notably, the thickness and width reductions are consistent across most fragments, suggesting that the mechanical behavior is nearly isotropic between the radial and axial directions. The average nominal strain at failure for the rings, based on the reduction in cross-sectional area, closely matches that of the cylinders, which is determined by the reduction in thickness, with both around $5\%$.

**Table 7:** Mean ($\mu$) and standard deviation (SD) of the distribution of neck-to-fragment ratio $N^{neck} / N_v$ corresponding to monolithic ring specimens, see Tables 37 to 54. Impact velocity range 1 – $248~\text {m}/\text {s} \le v_z \le 267~\text {m}/\text {s}$. Impact velocity range 2 – $354~\text {m}/\text {s} \le v_z \le 390~\text {m}/\text {s}$

	Impact velocity range 1	Impact velocity range 2
$\mu$	3.14	2.10
SD	1.72	1.22

**Fig. 32:**
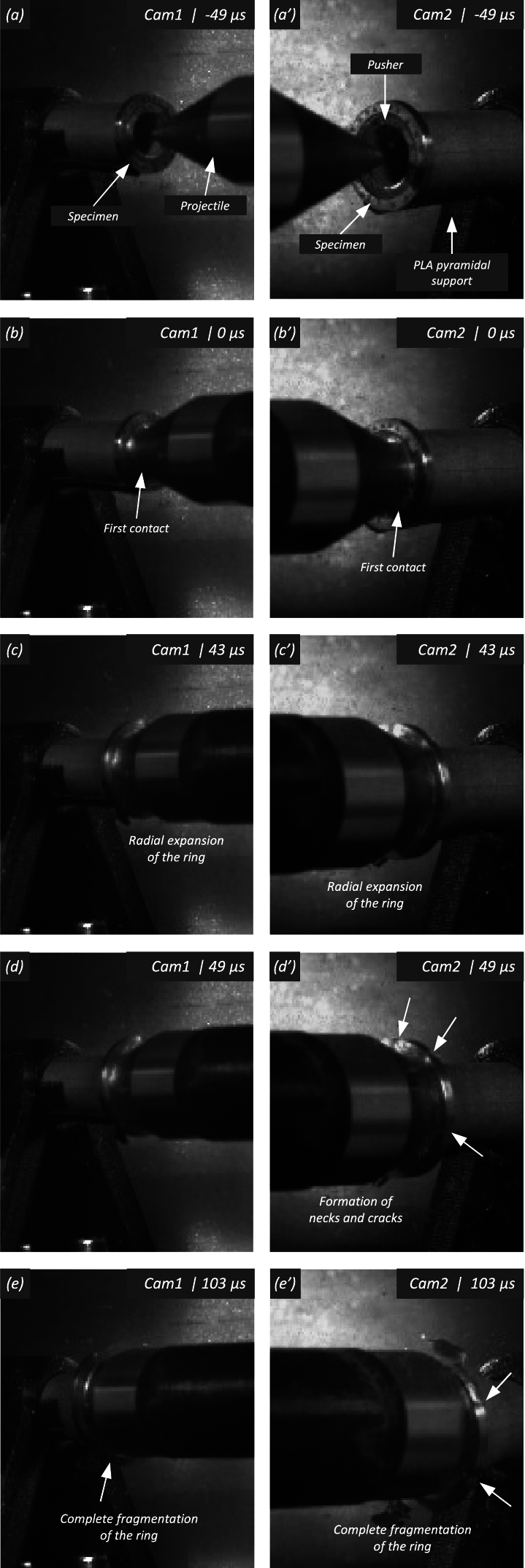
Sequence of images of the impact test for specimen R-Ti6Al4V-970-S-2 for different loading times: **a–a’** $\text {t}=-49~\upmu \text {s}$, **b–b’** $\text {t}=0~\upmu \text {s}$, **c–c’** $\text {t}=43~\upmu \text {s}$, **d–d’** $\text {t}=49~\upmu \text {s}$ and **e–e’** $\text {t}=103~\upmu \text {s}$. Images obtained by camera 1 are on the left side of the sequence, while those obtained by camera 2 are on the right side. The impact velocity is $v_{z} = 252.6~\text {m}/\text {s}$ (impact velocity range 1). For interpretation of the references to color in this figure, the reader is referred to the web version of this article

**Fig. 33:**
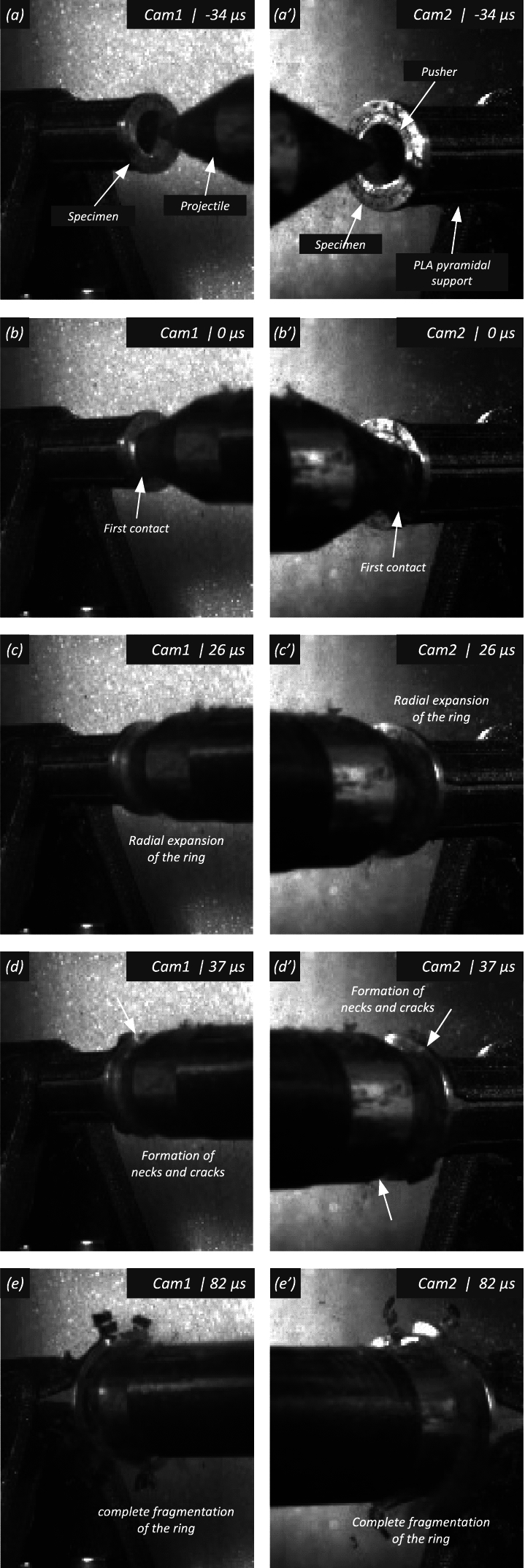
Sequence of images of the impact test for specimen R-Ti6Al4V-970-S-3 for different loading times: **a–a’** $\text {t}=-34~\upmu \text {s}$, **b–b’** $\text {t}=0~\upmu \text {s}$, **c–c’** $\text {t}=26~\upmu \text {s}$, **d–d’** $\text {t}=37~\upmu \text {s}$ and **e–e’** $\text {t}=82~\upmu \text {s}$. Images obtained by camera 1 are on the left side of the sequence, while those obtained by camera 2 are on the right side. The impact velocity is $v_{z} = 374.2~\text {m}/\text {s}$ (impact velocity range 2). For interpretation of the references to color in this figure, the reader is referred to the web version of this article

Figure 32 shows snapshots of the impact test on specimen R-Ti6Al4V-970-S-2 for different loading times. The striker velocity is $v_{z} = 252.6~\text {m}/\text {s}$ (impact velocity range 1), and the estimated strain rate is $10135.2~\text {s}^{-1}$. This specific experiment has been chosen due to the high-quality image recording and the clear identification of the uniform expansion, necking localization, and fragmentation processes. The first pair of images, snapshots (a)-(a’), was taken at $t = -49~\upmu \text {s}$, showing the striker approaching the pusher over which the ring specimen is inserted. The moment when the striker impacts the pusher is depicted in images (b)-(b’). Observe the uniform contact of the projectile nose with the inner circumference of the pusher. Following the impact, the projectile penetrates the hollow cylinder, expanding it and driving the metal ring outward in a radial direction, see (c)-(c’). To ensure homogeneous deformation in the ring during radial expansion, it is necessary for the contact between the projectile and the tube to remain uniform throughout the loading process. On the other hand, any deviation from perfect radial loading perturbs the mechanical fields in the ring and disrupts the symmetry of the problem, thereby favoring the formation of necks and cracks. Snapshots (e)-(e’) corresponding to loading time $t=49~\upmu \text {s}$ illustrate the nucleation of a series of necks throughout the circumference of the ring which are indicated with white arrows. Some of these necks develop into fractures, ultimately resulting in the complete fragmentation of the ring and the free-flight of the fragments, as depicted in (f)-(f’). The non-uniform spatial distribution of necks and fractures is likely attributable to spatial microstructural variations in the specimen, activating sections with lower necking strain, and to departures from perfect radial loading that may favor earlier localization of strains at specific sections of the specimen. Figure 33 shows images obtained from the video recording corresponding to specimen R-Ti6Al4V-970-S-3. The difference from the test in Fig. 32 is the increased impact velocity to $v_{z} = 374.2~\text {m/s}$. The mechanics of the ring expansion remain consistent, wherein the striker penetrates the pusher, causing the specimen to expand radially until a series of necks form (indicated with white arrows), and some of these develop into fractures. Similar observations are noted across all recordings involving monolithic ring specimens listed in Table 4, which are not presented here for the sake of brevity.

**Fig. 34:**
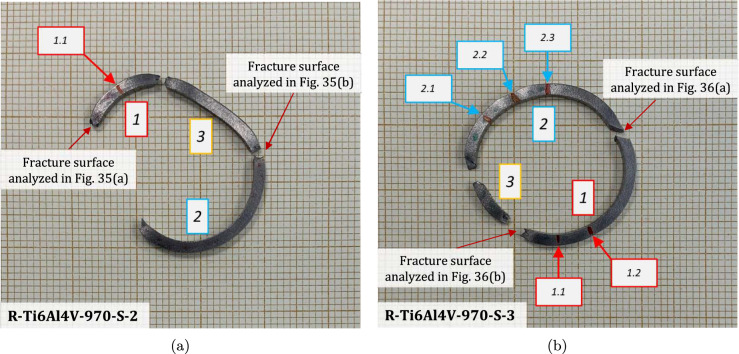
Post-mortem photography of the recovered fragments corresponding to specimens: **a** R-Ti6Al4V-970-S-2 tested at $v_{z} = 252.6~\text {m}/\text {s}$ (impact velocity range 1) and **b** R-Ti6Al4V-970-S-3 tested at $v_{z} = 374.2~\text {m}/\text {s}$ (impact velocity range 2). The fragments and necks are numbered the same as in Tables 42 and 43 of Appendix B. The fragments are labeled with single digits, while the arrested necks are labeled with two digits, the first of which corresponds to the fragment number. The color code is such that the necks match the color of their respective fragments. Millimeter graph paper is used as a reference for the dimensions

**Fig. 35:**
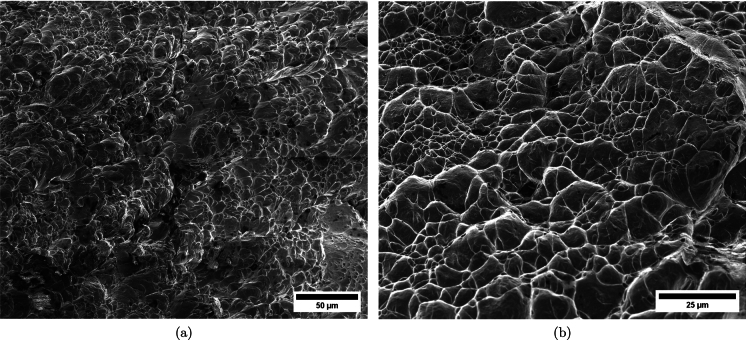
High-magnification SEM micrographs of fracture surfaces corresponding to specimen R-Ti6Al4V-970-S-2 tested at $v_{z} = 252.6~\text {m}/\text {s}$ (impact velocity range 1): **a** fracture of fragment 1 and **b** fracture between fragments 2 and 3. The fractures analyzed are indicated in Fig. 34a with red arrows. For interpretation of the references to color in this figure caption, the reader is referred to the web version of this article

**Fig. 36:**
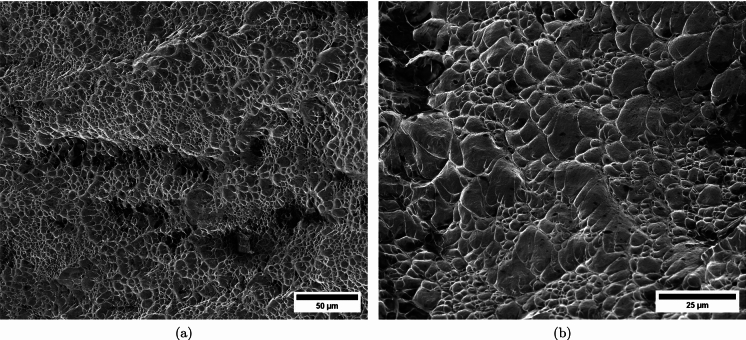
High-magnification SEM micrographs of fracture surfaces corresponding to specimen R-Ti6Al4V-970-S-3 tested at $v_{z} = 374.2~\text {m}/\text {s}$ (impact velocity range 2): **a** fracture between fragments 1 and 2 and **b** fracture between fragments 1 and 3. The fractures analyzed are indicated in Fig. 34b with red arrows. For interpretation of the references to color in this figure caption, the reader is referred to the web version of this article

Figure 34 presents postmortem photographs of the fragments recovered from the tests on samples R-Ti6Al4V-970-S-2 and R-Ti6Al4V-970-S-3, corresponding to the video recordings in Figs. 32 and 33, respectively. The fragments and necks are numbered the same as in Tables 42 and 43 of Appendix B. Similar to the case of the cylinder-shape samples, the fragments are labeled with single digits, while the arrested necks are labeled with two digits. The color of the necks corre33sponds to that of their respective fragments. Millimeter graph paper is used as a reference for the dimensions. Figure 34a shows the three fragments recuperated from ring R-Ti6Al4V-970-S-2 which corresponds to $88.9\%$ of the total mass of the specimen. The fractures develop in necked sections of the ring, characterized by localized thinning of the specimen’s cross-section, ultimately resulting in cup-cone ductile failure (Tvergaard and Needleman 1984). Recall that at the fracture sites, the reduced sample cross-section satisfies the necking criterion introduced in the first paragraph of Sect. 4. The development of well-defined necks, as opposed to the tests on cylindrical specimens, is likely attributed to the (relatively small) square cross-section of the samples which favors *easy* identification of necks. Note that, in addition to the necks leading to fractures, an arrested neck is observed within fragment 1, indicated by a red arrow and numbered 1.1. Figure 35 shows high-magnification SEM micrographs of the fracture surfaces marked with red arrows in Fig. 34a. Both the fracture of fragment 1 and the fracture between fragments 2 and 3 exhibit moderately elongated dimples, indicating a fracture mode characterized by tensile loading and eventual shear failure. This observation is consistent with the macroscopically observed cup-cone fracture pattern, where initial void growth in the center of the sample generally evolves into localized shear fracture along the maximum shear directions (Tvergaard and Needleman 1984). Figure 34b displays the three fragments formed in the test on sample R-Ti6Al4V-970-S-3, constituting $99.5\%$ of the total mass of the ring. Recall that the mass of the fragments does not match the mass of the ring before testing due to debris ejected during crack propagation and fragmentation. In addition to the necked sections where fractures occur, fragment 1 features two arrested necks, and fragment 2 presents three more. Note that the fractures between fragments 1-3 and 2-3 display a cup-cone shape, whereas the fracture between fragments 1-2 resembles a shear-dominated failure. Fractures displaying the cup-cone pattern typically align with more developed necks, which induce larger hydrostatic stress in the center of the specimen cross-section, consequently promoting void growth. Subsequent cracking in the center arises from the coalescence of voids under further strain, generating deformation shear bands at $45^{\circ }$ from the tensile axis and ultimately leading to the cup-cone pattern (Chen et al. 2018). Fractures displaying shear-dominated failure generally correspond to less developed necks. The fracture initiates near the specimen’s surface and propagates through the cross-section, following the direction of maximum shear stress. High-magnification SEM micrographs of the fracture surfaces marked in Fig. 34b with red arrows are shown in Fig. 36. While the images indicate ductile failure in both the fracture between fragments 1 and 2 and the fracture between fragments 1 and 3, the dimples exhibit shallow and elongated characteristics, highlighting local shear localization and failure.

**Fig. 37:**
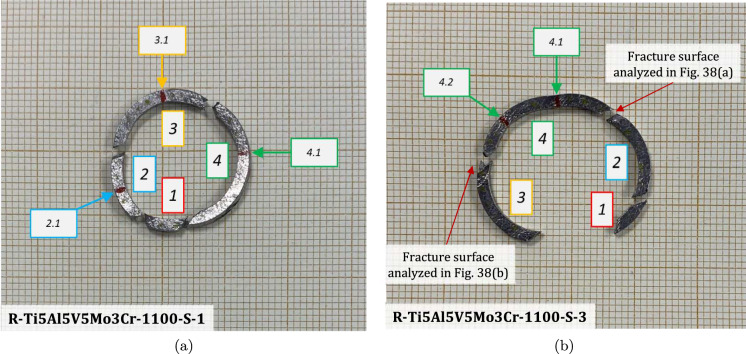
Post-mortem photography of the recovered fragments corresponding to specimens: (a) R-Ti5Al5V5Mo3Cr-1100-S-1 tested at $v_{z} = 254.0~\text {m}/\text {s}$ (impact velocity range 1) and (b) R-Ti5Al5V5Mo3Cr-1100-S-3 tested at $v_{z} = 371.4~\text {m}/\text {s}$ (impact velocity range 2). The fragments and necks are numbered the same as in Tables 50 and 52 of Appendix B. The fragments are labeled with single digits, while the arrested necks are labeled with two digits, the first of which corresponds to the fragment number. The color code is such that the necks match the color of their respective fragments. Millimeter graph paper is used as a reference for the dimensions

**Fig. 38:**
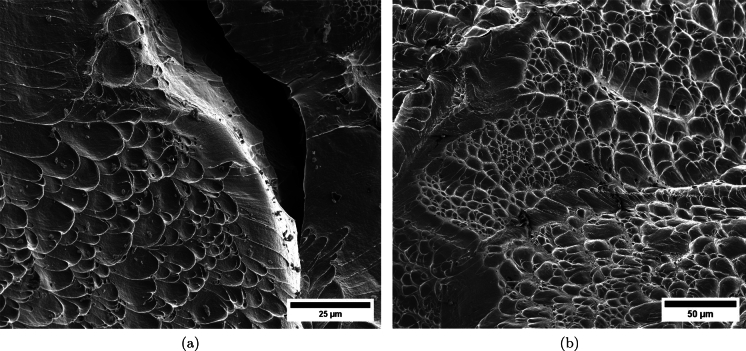
High-magnification SEM micrographs of fracture surfaces corresponding to specimen R-Ti5Al5V5Mo3Cr-1100-S-3 tested at $v_{z} = 371.4~\text {m}/\text {s}$ (impact velocity range 2): **a** fracture between fragments 2 and 4 and **b** fracture between fragments 3 and 4. The fractures analyzed are indicated in Fig. 37b with red arrows. For interpretation of the references to color in this figure caption, the reader is referred to the web version of this article

The formation of multiple necks and the coexistence of cup-cone fractures and shear-dominated fractures are observed in most of the tested rings. For instance, Fig. 37 shows post-mortem photography of the recovered fragments corresponding to specimens R-Ti5Al5V5Mo3Cr-1100-S-1 tested at $254.0~\text {m}/\text {s}$ and R-Ti5Al5V5Mo3Cr-1100-S-3 tested at $371.4~\text {m}/\text {s}$. The difference with the specimens in Fig. 34 is that the rings are made of Ti5Al5V5Mo3Cr and FAST processed at a higher dwell temperature of $1100~^{\circ }\text {C}$. Notably, in contrast to specimens FAST-processed at $970~^{\circ }\text {C}$, the surfaces of these samples after deformation exhibit a rough appearance reminiscent of an *orange peel*. This distinct texture, also known as deformation-induced surface roughening (Nie et al. 2021), appears consistent across other specimens FAST-processed at $1100~^{\circ }\text {C}$. The four recovered fragments of ring R-Ti5Al5V5Mo3Cr-1100-S-1 are shown in Fig. 37a. There is one arrested neck in fragments 2, 3, and 4. The fractures between fragments 1-2 and 3-4 exhibit the cup-and-cone shape, while the fractures in 1-4 and 2-3 resemble a shear-dominated failure. The four recovered fragments of ring R-Ti5Al5V5Mo3Cr-1100-S-3 are shown in Fig. 37b. Fragment 4 contains 2 arrested necks. Considering that all fractures occur at necked sections, more than half of the total number of necks nucleated develop into fractures. The fracture between fragments 3-4 displays the cup-and-cone shape (and eventually shear fracture), while the rest of the fractures exhibit shear-dominated failures accompanied by relatively less cross-section reduction. High-magnification SEM micrographs of the fracture surfaces indicated in Fig. 37b with red arrows are shown in Fig. 38. The images display large elliptical shallow dimples, which are representative of shear localization and fracture, akin to the fractography analysis depicted in Figs. 35 and 36 for samples R-Ti6Al4V-970-S-2 and R-Ti6Al4V-970-S-3. Moreover, the crack perpendicular to the main fracture surface in Fig. 38a bears resemblance to observations found by Draelos-Hagerty et al. (2023) in fracture surfaces of additively manufactured Ti6Al4V notched tensile specimens. The crack may stem from either a pre-existing material defect (similar cracks are commonly observed in mechanical tests of Ti6Al4V samples at the prior-beta grain boundary) or as a consequence of ejected debris during fragmentation.

**Fig. 39:**
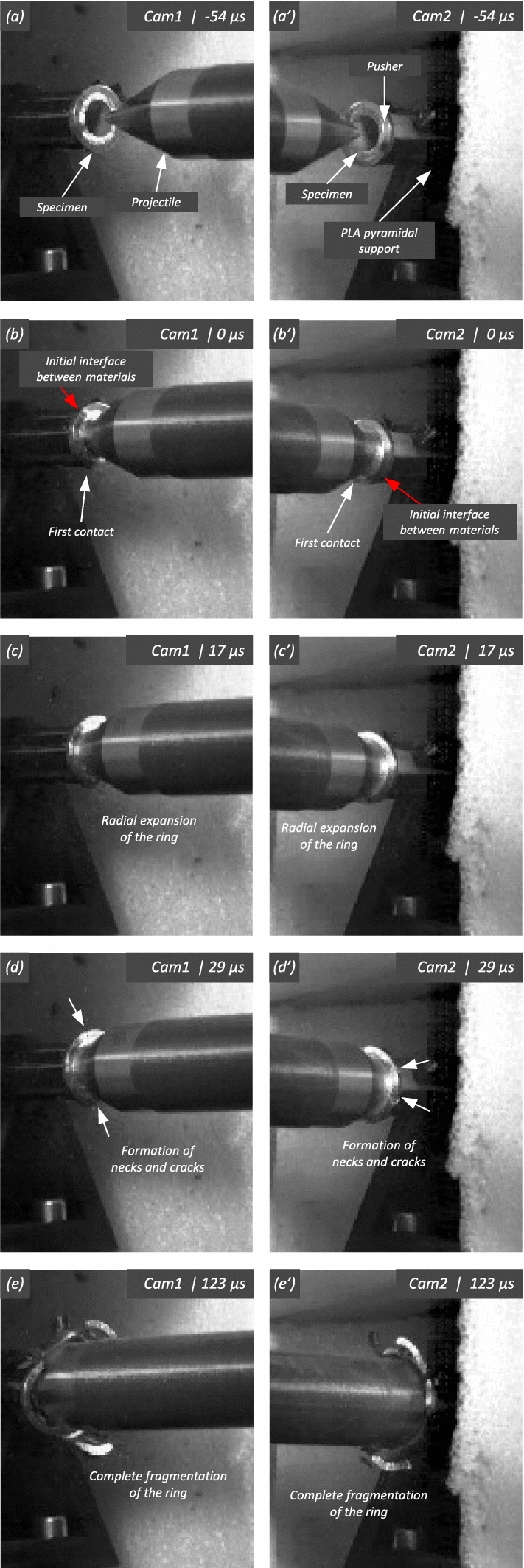
Sequence of images of the impact test for specimen R-Ti6Al4V/Ti5Al5V5Mo3Cr-1100-D-PL-1 for different loading times: **a–a’** $\text {t}=-54~\upmu \text {s}$, **b–b’** $\text {t}=0~\upmu \text {s}$, **c–c’** $\text {t}=17~\upmu \text {s}$, **d–d’** $\text {t}=29~\upmu \text {s}$ and **e–e’** $\text {t}=123~\upmu \text {s}$. Images obtained by camera 1 are on the left side of the sequence, while those obtained by camera 2 are on the right side. The impact velocity is $v_{z} = 258.1~\text {m}/\text {s}$ (impact velocity range 1). For interpretation of the references to color in this figure, the reader is referred to the web version of this article

**Fig. 40:**
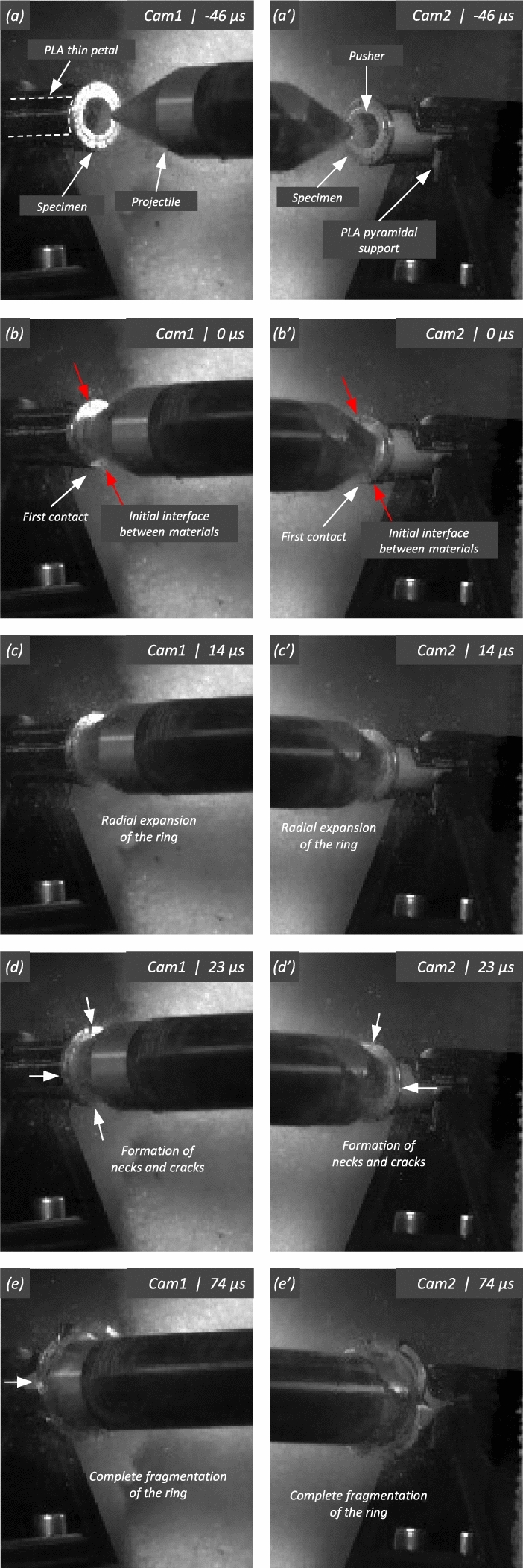
Sequence of images of the impact test for specimen R-Ti6Al4V/Ti5Al5V5Mo3Cr-1100-D-PL-3 for different loading times: **a–a’** $\text {t}=-46~\upmu \text {s}$, **b–b’** $\text {t}=0~\upmu \text {s}$, **c–c’** $\text {t}=14~\upmu \text {s}$, **d–d’** $\text {t}=23~\upmu \text {s}$ and **e–e’** $\text {t}=74~\upmu \text {s}$. Images obtained by camera 1 are on the left side of the sequence, while those obtained by camera 2 are on the right side. The impact velocity is $v_{z} = 368.5~\text {m}/\text {s}$ (impact velocity range 2). For interpretation of the references to color in this figure, the reader is referred to the web version of this article

### Multimaterial samples

**Fig. 41:**
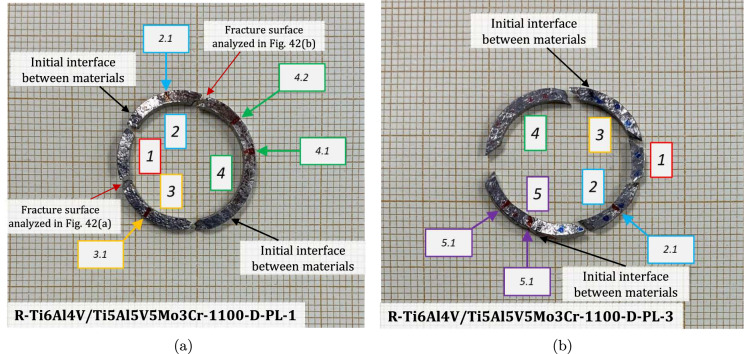
Post-mortem photography of the recovered fragments corresponding to specimens: **a** R-Ti6Al4V/Ti5Al5V5Mo3Cr-1100-D-PL-1 tested at $v_{z} = 258.1~\text {m}/\text {s}$ (impact velocity range 1) and **b** R-Ti6Al4V/Ti5Al5V5Mo3Cr-1100-D-PL-3 tested at $v_{z} = 368.5~\text {m}/\text {s}$ (impact velocity range 2). The fragments and necks are numbered the same as in Tables 60 and 62 of Appendix B. The fragments are labeled with single digits, while the arrested necks are labeled with two digits, the first of which corresponds to the fragment number. The color code is such that the necks match the color of their respective fragments. The interface between the two halves of the specimen made with Ti6Al4V and Ti5Al5V5Mo3Cr is indicated with black arrow. Millimeter graph paper is used as a reference for the dimensions

**Fig. 42:**
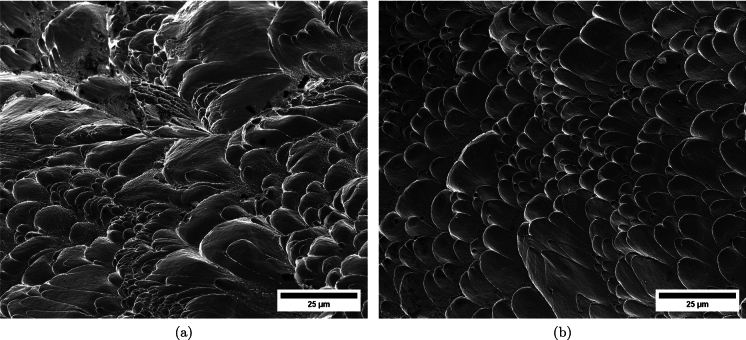
High-magnification SEM micrographs of fracture surfaces corresponding to specimen R-Ti6Al4V/Ti5Al5V5Mo3Cr-1100-D-PL-1 tested at $v_{z} = 258.1~\text {2 m}/\text {s}$ (impact velocity range 1): **a** fracture between fragments 1 and 3 corresponding to Ti5Al5V5Mo3Cr and **b** fracture between fragments 2 and 4 corresponding to Ti6Al4V. The fractures analyzed are indicated in Fig. 41a with red arrows. For interpretation of the references to color in this figure caption, the reader is referred to the web version of this article

Figures 39 and 40 show video recordings corresponding to the tests on specimens R-Ti6Al4V/Ti5Al5V5Mo3Cr-1100-D-PL-1 and R-Ti6Al4V/Ti5Al5V5Mo3Cr-1100-D-PL-3, respectively. The samples consist of two halves composed of Ti6Al4V and Ti5Al5V5Mo3Cr, which divide the ring along a plane parallel to its axis, see Fig. 1 and Table 4. The interface between the two materials is indicated with a red arrow in snapshots 39b-b’ and 40b-b’. The difference between the two experiments is the projectile impact velocity which is $258.1~\text {m}/\text {s}$ for R-Ti6Al4V/Ti5Al5V5Mo3Cr-1100-D-PL-1 (impact velocity range 1), and $368.5~\text {m}/\text {s}$ for R-Ti6Al4V/Ti5Al5V5Mo3Cr-1100-D-PL-3 (impact velocity range 2). The mechanisms governing ring expansion, necking formation and fragmentation are the same observed in the experiments discussed in Sect. 4.1. Notably, the interface between the two materials shows no apparent influence on the spatial location of necks and fractures (the same observation applies to all other multimaterial rings tested). Notice the *orange peel* texture on the specimens’ surface, akin to that observed in Fig. 37 for post-mortem rings that underwent FAST processing at a dwell temperature of $1100~^{\circ }\text {C}$. The orange peel texture is likely due to grain rotation during deformation, favored by the large grain size of the material. The four fragments recovered from sample R-Ti6Al4V/Ti5Al5V5Mo3Cr-1100-D-PL-1 corresponding to $99.7\%$ of the total mass of the ring are shown in Fig. 41a. The Ti6Al4V and Ti5Al5V5Mo3Cr sections were colored with brown and blue lines, respectively. The necks do not seem to be preferentially located in either of the two materials used to produce the sample, and neither the necks nor the fractures are situated at the interface between the two materials (as mentioned earlier). Figure 42 shows high-magnification SEM micrographs of the fracture surfaces indicated in Fig. 41a with red arrows. The fracture between fragments 1 and 3 correspond to Ti5Al5V5Mo3Cr, and the fracture between fragments 2 and 4 to Ti6Al4V. There are no significant differences observed in the morphology of the fracture surfaces between the two materials. Both the fractures in Ti6Al4V and Ti5Al5V5Mo3Cr exhibit large shallow elliptical dimples of comparable size and shape. Similar observations were obtained from the fractography analysis of all multi-material rings tested; however, the results are not shown for the sake of brevity. Figure 41b shows the five fragments recuperated from sample R-Ti6Al4V/Ti5Al5V5Mo3Cr-1100-D-PL-3. Blue and red dots were used to identify Ti6Al4V and Ti5Al5V5Mo3Cr sections, respectively. Fragment 2 contains a neck and fragment 5 two more. Similar to the results presented in Figs. 34 and 37, more than half of the total number of necks nucleated develop into fractures (considering that all fractures occur at necked sections). As for specimen R-Ti6Al4V/Ti5Al5V5Mo3Cr-1100-D-PL-1, none of the necks or fractures coincide with the interface between the two materials. These findings emphasize the notion that, in the fragmentation tests presented in this paper, the bonding between the two materials in the multimaterial specimens does not appear to be a weak link that promotes specimen fragmentation. This conclusion is in line with the results presented by Pope et al. (2019), showing that during tensile testing, failure predominantly occurs within the weaker alloy rather than at the interface of multimaterial specimens. Levano-Blanch et al. (2021) also observed this behavior during the tensile testing of multi-material FAST-DB samples, demonstrating that strain localization occurs in the weaker alloy.

## Concluding remarks

This paper investigates the mechanics of high-velocity fragmentation in titanium alloys fabricated through Field-Assisted Sintering Technology. Dynamic expansion tests were conducted on rings and cylinders at strain rates varying from $\approx 10050~\text {s}^{-1}$ to $\approx 19125~\text {s}^{-1}$. Two alloys, Ti6Al4V and Ti5Al5V5Mo3Cr, processed at different dwell temperatures, were tested, including both monolithic and multi-material samples. Fragments were collected, weighed, sized, and analyzed using scanning electron microscopy. The experiments revealed that as the expansion velocity increases, the number of necks and fragments, as well as the proportion of necks developing into fragments, generally increase, while the range of fragment lengths and neck spacings narrows. This results in a reduction in both the mean and standard deviation of the fragment size and neck-free segment distributions. The average distance between necks was compared with predictions from linear stability analysis, showing good agreement between theory and experiments. The fractographic analysis revealed that the fracture of both rings and cylinders is essentially ductile, caused by the micro-void nucleation, growth, and coalescence. The fracture surfaces exhibit a morphology that combines nearly spherical dimples typical of tensile cup-cone failure with shallow elliptical dimples characteristic of shear-dominated failure. Various fractures exhibit varying degrees of dominance in spherical and elliptical dimples, indicating that the stress state at the fracture level varies from one crack to another. The cup-cone fracture is usually observed in well-developed necked sections, while the shear fracture is generally accompanied by relatively less cross-sectional reduction of the samples. The formation of shear fractures inclined at $45^{\circ }$ with respect to the circumferential direction of the specimens is attributed to the susceptibility of titanium alloys to develop shear plastic localization. On the other hand, no discernible differences have been observed in the deformation and fracture mechanisms between Ti6Al4V and Ti5Al5V5Mo3Cr. Furthermore, in all tests conducted with multi-material specimens, necks and fractures nucleated away from the interface between the two alloys, highlighting the strength of the bond between Ti6Al4V and Ti5Al5V5Mo3Cr, which exhibited no signs of structural weakness. These outcomes underscore the efficacy of the FAST technique in fabricating titanium multi-material structural systems resilient to impact loads.

Additional future work will involve comparing the fragmentation experiments performed on FAST specimens with tests conducted on titanium samples manufactured using other techniques, such as forging and 3D printing, to investigate the effect of microstructure on the dynamic fracture behavior and energy absorption capacity of titanium alloys under high-velocity impacts.

**Table 8:** List of fragments size and mass for C-Ti6Al4V-970-S-2. The impact velocity is $v_{z} = 267.6~\text {m}/\text {s}$ (impact velocity range 1)

*Fragment*	$L_{\theta }~(\text {mm})$	$L_z~(\text {mm})$	$t~(\text {mm})$	$m~(\text {g})$
1	22.84	19.52	0.97	3.613
2	7.29	9.60	0.98	0.341
3	18.05	12.79	1.03	1.980

**Table 9:** List of fragments size and mass for C-Ti6Al4V-970-S-4. The impact velocity is $v_{z} = 355.3~\text {m}/\text {s}$ (impact velocity range 2)

*Fragment*	$L_{\theta }~(\text {mm})$	$L_z~(\text {mm})$	$t~(\text {mm})$	$m~(\text {g})$
1	10.31	28.74	0.92	1.418
2	6.52	6.56	0.88	0.184
3	18.49	28.37	0.93	4.074

**Table 10:** List of fragments size and mass for C-Ti6Al4V-970-S-5. The impact velocity is $v_{z} = 362.7~\text {m}/\text {s}$ (impact velocity range 2)

*Fragment*	$L_{\theta }~(\text {mm})$	$L_z~(\text {mm})$	$t~(\text {mm})$	$m~(\text {g})$
1	5.18	11.12	1.03	0.305
2	32.06	30.39	0.93	5.587

**Table 11:** List of fragments size and mass for C-Ti6Al4V-970-S-6. The impact velocity is $v_{z} = 370.1~\text {m}/\text {s}$ (impact velocity range 2)

*Fragment*	$L_{\theta }~(\text {mm})$	$L_z~(\text {mm})$	$t~(\text {mm})$	$m~(\text {g})$
1	20.69	21.10	0.90	2.577
2	6.63	28.96	1.03	0.679
3	16.21	12.22	1.00	1.509
4	13.52	34.89	0.92	2.017

**Table 12:** List of fragments size and mass for C-Ti6Al4V-1100-S-1. The impact velocity is $v_{z} = 255.51~\text {m}/\text {s}$ (impact velocity range 1)

*Fragment*	$L_{\theta }~(\text {mm})$	$L_z~(\text {mm})$	$t~(\text {mm})$	$m~(\text {g})$
1	11.67	38.43	1.01	2.104
2	7.92	19.15	1.00	0.607
3	10.21	23.17	1.02	1.199
4	10.27	25.97	1.05	1.286
4	9.97	29.66	0.99	1.580

## Data Availability

No datasets were generated or analysed during the current study.

## Appendix A. List of fragments from cylinder expansion tests

This section provides the circumferential length $L_{\theta }~(\text {mm})$, axial length $L_z~(\text {mm})$, thickness $t~(\text {mm})$ and mass $m~(\text {g})$ of all fragments recovered from the impact experiments performed on FAST-processed titanium cylindrical specimens.

**Table 13:** List of fragments size and mass for C-Ti6Al4V-1100-S-2. The impact velocity is $v_{z} = 261.5~\text {m}/\text {s}$ (impact velocity range 1)

*Fragment*	$L_{\theta }~(\text {mm})$	$L_z~(\text {mm})$	$t~(\text {mm})$	$m~(\text {g})$
1	18.12	37.62	0.93	3.754
2	7.62	9.34	1.02	0.289
3	10.26	11.89	0.95	0.593
4	9.69	8.34	1.02	0.369
5	7.31	11.63	0.99	0.394
6	6.54	19.49	1.01	0.569
7	6.35	21.30	0.99	0.817

**Table 14:** List of fragments size and mass for C-Ti6Al4V-1100-S-3. The impact velocity is $v_{z} = 256.4~\text {m}/\text {s}$ (impact velocity range 1)

*Fragment*	$L_{\theta }~(\text {mm})$	$L_z~(\text {mm})$	$t~(\text {mm})$	$m~(\text {g})$
1	11.33	21.10	0.99	1.656
2	8.41	10.38	1.04	0.413
3	8.89	19.62	1.01	0.802
4	12.47	11.73	0.97	0.843
5	13.25	11.99	0.96	1.495

**Table 15:** List of fragments size and mass for C-Ti6Al4V-1100-S-4. The impact velocity is $v_{z} = 364.5~\text {m}/\text {s}$ (impact velocity range 2)

*Fragment*	$L_{\theta }~(\text {mm})$	$L_z~(\text {mm})$	$t~(\text {mm})$	$m~(\text {g})$
1	12.69	21.38	0.99	1.391
2	5.77	6.25	0.97	0.241
3	6.56	6.04	0.98	0.175
4	6.53	5.80	0.94	0.163
5	9.43	14.70	0.97	0.629
6	6.23	14.66	0.97	0.341
7	8.37	40.00	0.97	1.269
8	8.37	40.00	0.95	1.324
9	8.41	25.83	0.91	1.078

**Table 16:** List of fragments size and mass for C-Ti6Al4V-1100-S-5. The impact velocity is $v_{z} = 354.3~\text {m}/\text {s}$ (impact velocity range 2)

*Fragment*	$L_{\theta }~(\text {mm})$	$L_z~(\text {mm})$	$t~(\text {mm})$	$m~(\text {g})$
1	9.06	25.18	0.98	1.415
2	15.10	12.19	0.93	1.386
3	7.84	23.78	0.97	0.805
4	7.60	19.19	0.97	0.633
5	12.28	10.47	1.00	0.681
6	4.82	12.19	0.96	0.242
7	9.93	23.28	0.93	1.063

**Table 17:** List of fragments size and mass for C-Ti6Al4V-1100-S-6. The impact velocity is $v_{z} = 360.7~\text {m}/\text {s}$ (impact velocity range 2)

*Fragment*	$L_{\theta }~(\text {mm})$	$L_z~(\text {mm})$	$t~(\text {mm})$	$m~(\text {g})$
1	8.31	10.73	0.93	0.379
2	9.00	40.00	0.91	1.452
3	11.72	27.60	0.92	1.274
4	7.11	13.34	0.98	0.368
5	6.48	11.80	0.94	0.241
6	6.89	19.35	0.94	0.522
7	6.56	16.10	0.95	0.387
8	8.26	15.67	0.92	0.548
9	6.72	6.90	0.98	0.233
10	8.72	8.58	0.98	0.340
11	9.32	21.72	0.98	0.826

**Table 18:** List of fragments size and mass for C-Ti5Al5V5Mo3Cr-970-S-1. The impact velocity is $v_{z} = 255.5~\text {m}/\text {s}$ (impact velocity range 1)

*Fragment*	$L_{\theta }~(\text {mm})$	$L_z~(\text {mm})$	$t~(\text {mm})$	$m~(\text {g})$
1	14.50	40.00	0.89	2.286
2	9.56	40.00	0.88	1.856
3	9.76	19.76	0.99	1.009
4	8.90	22.00	0.97	0.990
5	7.34	24.78	0.96	0.815

**Table 19:** List of fragments size and mass for C-Ti5Al5V5Mo3Cr-970-S-2. The impact velocity is $v_{z} = 261.5~\text {m}/\text {s}$ (impact velocity range 1)

*Fragment*	$L_{\theta }~(\text {mm})$	$L_z~(\text {mm})$	$t~(\text {mm})$	$m~(\text {g})$
1	12.75	40.00	0.90	2.281
2	10.68	40.00	0.91	2.056
3	8.82	18.49	0.99	1.314
4	9.70	20.40	0.99	0.872
5	9.04	13.91	0.99	0.567

**Table 20:** List of fragments size and mass for C-Ti5Al5V5Mo3Cr-970-S-3. The impact velocity is $v_{z} = 259.1~\text {m}/\text {s}$ (impact velocity range 1)

*Fragment*	$L_{\theta }~(\text {mm})$	$L_z~(\text {mm})$	$t~(\text {mm})$	$m~(\text {g})$
1	30.76	19.37	0.97	3.048
2	6.79	11.84	1.00	0.345
3	8.58	11.31	1.00	0.436
4	15.99	13.52	0.94	1.234
5	15.16	17.80	0.82	1.957

**Table 21:** List of fragments size and mass for C-Ti5Al5V5Mo3Cr-970-S-4. The impact velocity is $v_{z} = 374.5~\text {m}/\text {s}$ (impact velocity range 2)

*Fragment*	$L_{\theta }~(\text {mm})$	$L_z~(\text {mm})$	$t~(\text {mm})$	$m~(\text {g})$
1	10.84	40.00	0.91	1.484
2	10.27	11.03	0.94	0.624
3	7.99	12.80	1.00	0.437
4	7.63	40.00	0.95	1.161
5	8.24	24.66	0.96	0.924
6	7.24	12.99	0.99	0.364
7	5.63	20.01	0.95	0.573
8	7.63	40.00	0.93	1.363

**Table 22:** List of fragments size and mass for C-Ti5Al5V5Mo3Cr-970-S-5. The impact velocity is $v_{z} = 368.8~\text {m}/\text {s}$ (impact velocity range 2)

*Fragment*	$L_{\theta }~(\text {mm})$	$L_z~(\text {mm})$	$t~(\text {mm})$	$m~(\text {g})$
1	6.10	21.99	0.99	0.752
2	6.26	21.20	0.95	0.645
3	7.92	17.93	1.02	0.628
4	9.65	12.89	0.91	0.735
5	13.90	17.70	0.90	1.682
6	8.43	21.40	0.97	0.759
7	11.70	14.77	0.92	0.742

**Table 23:** List of fragments size and mass for C-Ti5Al5V5Mo3Cr-970-S-6. The impact velocity is $v_{z} = 356.6~\text {m}/\text {s}$ (impact velocity range 2)

*Fragment*	$L_{\theta }~(\text {mm})$	$L_z~(\text {mm})$	$t~(\text {mm})$	$m~(\text {g})$
1	8.87	40.00	0.93	1.378
2	11.67	8.79	0.89	0.515
3	6.89	22.28	1.00	0.691
4	7.93	40.00	0.93	1.161
5	6.59	8.10	0.97	0.238
6	5.90	18.76	0.96	0.466
7	10.97	24.25	0.87	1.392
8	7.87	40.00	0.94	1.179

**Table 24:** List of fragments size and mass for C-Ti6Al4V/Ti5Al5V5Mo3Cr-970-D-PP-1. The impact velocity is $v_{z} = 261.3~\text {m}/\text {s}$ (impact velocity range 1)

*Fragment*	$L_{\theta }~(\text {mm})$	$L_z~(\text {mm})$	$t~(\text {mm})$	$m~(\text {g})$
1	12.02	16.20	0.85	0.943
2	8.43	10.56	0.98	0.549

**Table 25:** List of fragments size and mass for C-Ti6Al4V/Ti5Al5V5Mo3Cr-970-D-PP-2. The impact velocity is $v_{z} = 261.4~\text {m}/\text {s}$ (impact velocity range 1)

*Fragment*	$L_{\theta }~(\text {mm})$	$L_z~(\text {mm})$	$t~(\text {mm})$	$m~(\text {g})$
1	12.65	10.98	0.90	1.278
2	9.97	11.41	0.91	0.946
3	11.95	16.55	0.93	1.080
4	5.69	9.00	0.98	0.650
5	11.23	21.57	0.96	1.183
6	8.57	9.19	0.89	0.585
7	9.46	10.25	0.98	0.662
8	7.41	13.24	1.00	0.739

**Table 26:** List of fragments size and mass for C-Ti6Al4V/Ti5Al5V5Mo3Cr-970-D-PP-3. The impact velocity is $v_{z} = 354.5~\text {m}/\text {s}$ (impact velocity range 2)

*Fragment*	$L_{\theta }~(\text {mm})$	$L_z~(\text {mm})$	$t~(\text {mm})$	$m~(\text {g})$
1	17.06	24.91	0.89	2.212
2	9.27	10.57	0.97	1.196
3	27.04	40.00	0.85	3.795

**Table 27:** List of fragments size and mass for C-Ti6Al4V/Ti5Al5V5Mo3Cr-970-D-PP-4. The impact velocity is $v_{z} = 255.0~\text {m}/\text {s}$ (impact velocity range 1)

*Fragment*	$L_{\theta }~(\text {mm})$	$L_z~(\text {mm})$	$t~(\text {mm})$	$m~(\text {g})$
1	13.01	17.19	0.96	1.379
2	11.72	40.00	0.92	2.407
3	9.72	21.15	0.88	1.370
4	4.17	22.95	0.99	0.912
5	6.89	40.00	0.95	1.205

**Table 28:** List of fragments size and mass for C-Ti6Al4V/Ti5Al5V5Mo3Cr-970-D-PP-6. The impact velocity is $v_{z} = 374.2~\text {m}/\text {s}$ (impact velocity range 2)

*Fragment*	$L_{\theta }~(\text {mm})$	$L_z~(\text {mm})$	$t~(\text {mm})$	$m~(\text {g})$
1	11.20	40.00	0.91	1.932
2	11.42	16.73	0.95	1.434
3	7.36	19.96	0.96	0.676
4	7.21	8.81	0.94	0.314
5	8.42	13.45	0.94	0.453
6	6.21	7.21	0.94	0.221
7	10.49	11.06	0.99	0.695
8	8.34	31.82	0.91	1.129
9	6.38	11.95	0.99	0.392

**Table 29:** List of fragments size and mass for C-Ti6Al4V/Ti5Al5V5Mo3Cr-970-D-PL-1. The impact velocity is $v_{z} = 362.6~\text {m}/\text {s}$ (impact velocity range 2)

*Fragment*	$L_{\theta }~(\text {mm})$	$L_z~(\text {mm})$	$t~(\text {mm})$	$m~(\text {g})$
1	12.23	40.00	0.92	2.161
2	5.64	23.92	0.98	0.747
3	8.17	22.54	0.93	0.924
4	6.26	17.25	1.00	0.459
5	9.04	40.00	0.87	1.682
6	6.41	10.75	0.97	0.294
7	6.52	19.43	1.02	0.499

**Table 30:** List of fragments size and mass for C-Ti6Al4V/Ti5Al5V5Mo3Cr-970-D-PL-2. The impact velocity is $v_{z} = 254.7~\text {m}/\text {s}$ (impact velocity range 1)

*Fragment*	$L_{\theta }~(\text {mm})$	$L_z~(\text {mm})$	$t~(\text {mm})$	$m~(\text {g})$
1	11.19	17.63	0.87	1.637
2	7.89	22.65	0.96	0.830
3	14.28	11.36	0.94	1.078
4	8.05	21.10	0.95	0.878
5	14.54	26.46	0.97	2.872

**Table 31:** List of fragments size and mass for C-Ti6Al4V/Ti5Al5V5Mo3Cr-970-D-PL-3. The impact velocity is $v_{z} = 248.8~\text {m}/\text {s}$ (impact velocity range 1)

*Fragment*	$L_{\theta }~(\text {mm})$	$L_z~(\text {mm})$	$t~(\text {mm})$	$m~(\text {g})$
1	11.40	40.00	0.93	2.141
2	12.93	19.14	0.94	2.242
3	4.80	23.47	1.00	0.635
4	6.42	27.53	1.00	0.810
5	12.38	10.91	0.98	0.680

**Table 32:** List of fragments size and mass for C-Ti6Al4V/Ti5Al5V5Mo3Cr-970-D-PL-4. The impact velocity is $v_{z} = 389.8~\text {m}/\text {s}$ (impact velocity range 2)

*Fragment*	$L_{\theta }~(\text {mm})$	$L_z~(\text {mm})$	$t~(\text {mm})$	$m~(\text {g})$
1	11.24	40.00	0.91	2.063
2	9.80	27.70	0.98	0.950
3	7.82	7.43	0.97	0.321
4	11.99	12.66	0.95	0.666
5	6.85	16.54	0.97	0.426
6	6.38	18.77	0.98	0.558
7	11.51	23.25	0.89	1.906

**Table 33:** List of fragments size and mass for C-Ti6Al4V/Ti5Al5V5Mo3Cr-1100-D-PL-1. The impact velocity is $v_{z} = 266.0~\text {m}/\text {s}$ (impact velocity range 1)

*Fragment*	$L_{\theta }~(\text {mm})$	$L_z~(\text {mm})$	$t~(\text {mm})$	$m~(\text {g})$
1	9.70	13.08	1.00	0.765
2	6.72	40.00	0.89	1.128
3	7.42	27.95	0.96	1.091
4	10.13	20.99	0.93	0.975

**Table 34:** List of fragments size and mass for C-Ti6Al4V/Ti5Al5V5Mo3Cr-1100-D-PL-2. The impact velocity is $v_{z} = 387.5~\text {m}/\text {s}$ (impact velocity range 2)

*Fragment*	$L_{\theta }~(\text {mm})$	$L_z~(\text {mm})$	$t~(\text {mm})$	$m~(\text {g})$
1	12.68	40.00	0.97	2.145
2	8.52	13.50	0.87	0.858
3	8.07	10.85	1.01	0.445
4	6.03	24.80	1.02	0.729
5	6.59	23.64	0.96	0.772
6	8.34	14.41	0.91	0.563
7	5.50	13.67	0.99	0.285
8	7.00	11.90	1.02	0.457
9	6.38	28.52	0.96	0.737

**Table 35:** List of fragments size and mass for C-Ti6Al4V/Ti5Al5V5Mo3Cr-1100-D-PL-3. The impact velocity is $v_{z} = 263.7~\text {m}/\text {s}$ (impact velocity range 1)

*Fragment*	$L_{\theta }~(\text {mm})$	$L_z~(\text {mm})$	$t~(\text {mm})$	$m~(\text {g})$
1	18.07	40.00	0.98	2.729
2	8.46	34.07	0.96	1.236
3	5.87	21.30	0.95	0.566
4	9.62	16.86	0.94	0.737
5	7.86	19.50	0.97	0.603
6	6.66	7.62	0.98	0.243
7	5.84	21.21	0.95	0.544
8	10.41	6.87	0.92	0.342

**Table 36:** List of fragments size and mass for C-Ti6Al4V/Ti5Al5V5Mo3Cr-1100-D-PL-4. The impact velocity is $v_{z} = 382.1~\text {m}/\text {s}$ (impact velocity range 2)

*Fragment*	$L_{\theta }~(\text {mm})$	$L_z~(\text {mm})$	$t~(\text {mm})$	$m~(\text {g})$
1	5.01	15.02	0.99	0.289
2	5.22	17.94	0.98	0.377
3	8.51	9.82	0.95	0.345
4	5.71	6.22	0.94	0.105
5	7.46	26.06	0.98	0.872
6	7.49	40.00	0.93	1.067
7	8.73	10.50	0.94	0.422
8	9.11	27.41	0.95	1.092
9	8.73	40.00	0.95	1.584

**Table 37:** List of fragments size and mass for R-Ti5Al5V5Mo3Cr-970-S-1. The impact velocity is $v_{z} = 254.1~\text {m}/\text {s}$ (impact velocity range 1)

*Fragment*	$L_{\theta }$ (mm)	$L_z~(\text {mm})$	$t~(\text {mm})$	$m~(\text {g})$	$L_{\theta }^{neck}$ (mm)
1	61.00	1.93	1.82	0.870	5.25/5.75/13.50/4.75/4.50/21.25/6.00

**Table 38:** List of fragments size and mass for R-Ti5Al5V5Mo3Cr-970-S-3. The impact velocity is $v_{z} = 260.5~\text {m}/\text {s}$ (impact velocity range 1)

*Fragment*	$L_{\theta }$ (mm)	$L_z~(\text {mm})$	$t~(\text {mm})$	$m~(\text {g})$	$L_{\theta }^{neck}$ (mm)
1	49.00	1.97	1.93	0.722	7.25/22.75/10.75/8.25
2	17.50	1.92	1.87	0.232	9.50/8.00

**Table 39:** List of fragments size and mass for R-Ti5Al5V5Mo3Cr-970-S-4. The impact velocity is $v_{z} = 369.8~\text {m}/\text {s}$ (impact velocity range 2)

*Fragment*	$L_{\theta }$ (mm)	$L_z~(\text {mm})$	$t~(\text {mm})$	$m~(\text {g})$	$L_{\theta }^{neck}$ (mm)
1	36.00	2.00	1.98	0.549	20.00/16.00
2	10.97	1.97	1.98	0.158	10.97
3	13.63	1.99	1.95	0.227	13.63

## Appendix B. List of fragments from ring expansion tests

This section provides the circumferential length $L_{\theta }~(\text {mm})$, width $w~(\text {mm})$, thickness $t~(\text {mm})$ and mass $m~(\text {g})$ of all fragments recovered, and the necks spacing measured in the recovered fragments $L_{\theta }^{neck}~(\text {mm})$, for the impact experiments performed on FAST-processed titanium ring specimens.

**Table 40:** List of fragments size and mass for R-Ti5Al5V5Mo3Cr-970-S-5. The impact velocity is $v_{z} = 370.4~\text {m}/\text {s}$ (impact velocity range 2)

*Fragment*	$L_{\theta }$ (mm)	$L_z~(\text {mm})$	$t~(\text {mm})$	$m~(\text {g})$	$L_{\theta }^{neck}$ (mm)
1	17.25	1.93	1.81	0.236	9.50/7.75
2	27.50	1.91	1.81	0.393	8.00/19.50
3	18.00	1.91	1.79	0.231	8.25/4.75/5.00

**Table 41:** List of fragments size and mass for R-Ti6Al4V-970-S-1. The impact velocity is $v_{z} = 250.6~\text {m}/\text {s}$ (impact velocity range 1)

*Fragment*	$L_{\theta }$ (mm)	$L_z~(\text {mm})$	$t~(\text {mm})$	$m~(\text {g})$	$L_{\theta }^{neck}$ (mm)
1	30.75	1.83	1.76	0.363	14.75/7.75/8.25
2	38.75	1.90	1.88	0.530	31.75/7.00

**Table 42:** List of fragments size and mass for R-Ti6Al4V-970-S-2. The impact velocity is $v_{z} = 252.6~\text {m}/\text {s}$ (impact velocity range 1)

*Fragment*	$L_{\theta }$ (mm)	$L_z~(\text {mm})$	$t~(\text {mm})$	$m~(\text {g})$	$L_{\theta }^{neck}$ (mm)
1	13.25	1.88	1.89	0.164	6.50/6.75
2	29.75	1.79	1.78	0.365	29.75
3	18.00	1.80	1.81	0.238	18.00

**Table 43:** List of fragments size and mass for R-Ti6Al4V-970-S-3. The impact velocity is $v_{z} = 374.2~\text {m}/\text {s}$ (impact velocity range 2)

*Fragment*	$L_{\theta }$ (mm)	$L_z~(\text {mm})$	$t~(\text {mm})$	$m~(\text {g})$	$L_{\theta }^{neck}$ (mm)
1	29.50	1.81	1.80	0.362	6.50/5.75/17.25
2	33.75	1.84	1.83	0.421	9.25/5.75/6.25/12.50
3	7.18	1.79	1.80	0.084	7.18

**Table 44:** List of fragments size and mass for R-Ti6Al4V-970-S-4. The impact velocity is $v_{z} = 369.5~\text {m}/\text {s}$ (impact velocity range 2)

*Fragment*	$L_{\theta }$ (mm)	$L_z~(\text {mm})$	$t~(\text {mm})$	$m~(\text {g})$	$L_{\theta }^{neck}$ (mm)
1	21.25	1.88	1.86	0.245	18.00/15.00/17.50
2	50.50	1.86	1.86	0.637	50.50

**Table 45:** List of fragments size and mass for R-Ti6Al4V-970-S-5. The impact velocity is $v_{z} = 369.5~\text {m}/\text {s}$ (impact velocity range 2)

*Fragment*	$L_{\theta }$ (mm)	$L_z~(\text {mm})$	$t~(\text {mm})$	$m~(\text {g})$	$L_{\theta }^{neck}$ (mm)
1	34.75	1.76	1.76	0.411 7.25/6.50/5.50/15.50	
2	38.50	1.78	1.76	0.452	6.25/6.50/5.00/4.25/6.00/10.50

**Table 46:** List of fragments size and mass for R-Ti6Al4V-1100-S-1. The impact velocity is $v_{z} = 256.4~\text {m}/\text {s}$ (impact velocity range 1)

*Fragment*	$L_{\theta }$ (mm)	$L_z~(\text {mm})$	$t~(\text {mm})$	$m~(\text {g})$	$L_{\theta }^{neck}$ (mm)
1	18.25	1.97	1.99	0.273	6.50/11.75
2	40.00	1.97	2.00	0.597	28.00/12.00

**Table 47:** List of fragments size and mass for R-Ti6Al4V-1100-S-2. The impact velocity is $v_{z} = 251.0~\text {m}/\text {s}$ (impact velocity range 1)

*Fragment*	$L_{\theta }$ (mm)	$L_z~(\text {mm})$	$t~(\text {mm})$	$m~(\text {g})$	$L_{\theta }^{neck}$ (mm)
1	5.83	2.02	1.91	0.077	5.83
2	20.75	2.01	1.97	0.341	20.75
3	32.50	2.01	1.96	0.482	14.25/5.00/13.25

**Table 48:** List of fragments size and mass for R-Ti6Al4V-1100-S-3. The impact velocity is $v_{z} = 371.2~\text {m}/\text {s}$ (impact velocity range 2)

*Fragment*	$L_{\theta }$ (mm)	$L_z~(\text {mm})$	$t~(\text {mm})$	$m~(\text {g})$	$L_{\theta }^{neck}$ (mm)
1	13.75	1.94	1.93	0.198	4.75/5.50/3.50
2	15.75	1.92	1.92	0.215	8.00/7.75
3	31.75	1.93	1.92	0.436	23.50/8.25

**Table 49:** List of fragments size and mass for R-Ti6Al4V-1100-S-4. The impact velocity is $v_{z} = 369.8~\text {m}/\text {s}$ (impact velocity range 2)

*Fragment*	$L_{\theta }$ (mm)	$L_z~(\text {mm})$	$t~(\text {mm})$	$m~(\text {g})$	$L_{\theta }^{neck}$ (mm)
1	7.00	1.93	1.90	0.118	7.00
2	11.75	1.91	1.92	0.145	6.25/5.50
3	19.00	1.94	1.94	0.257	19.00
4	25.75	1.94	1.95	0.356	4.25/6.25/15.25

**Table 50:** List of fragments size and mass for R-Ti5Al5V5Mo3Cr-1100-S-1. The impact velocity is $v_{z} = 254.0~\text {m}/\text {s}$ (impact velocity range 1)

*Fragment*	$L_{\theta }$ (mm)	$L_z~(\text {mm})$	$t~(\text {mm})$	$m~(\text {g})$	$L_{\theta }^{neck}$ (mm)
1	5.48	2.00	1.97	0.095	5.48
2	11.00	1.96	1.95	0.164	5.25/5.75
3	20.50	1.99	1.97	0.287	7.25/13.25
4	27.75	1.97	1.96	0.397	17.5/10.25

**Table 51:** List of fragments size and mass for R-Ti5Al5V5Mo3Cr-1100-S-2. The impact velocity is $v_{z} = 256.0~\text {m}/\text {s}$ (impact velocity range 1)

*Fragment*	$L_{\theta }$ (mm)	$L_z~(\text {mm})$	$t~(\text {mm})$	$m~(\text {g})$	$L_{\theta }^{neck}$ (mm)
1	17.50	1.95	1.94	0.224	9.75/7.75
2	43.50	1.93	1.95	0.695	4.00/7.00/5.75/5.50/11.25/4.25/5.75

**Table 52:** List of fragments size and mass for R-Ti5Al5V5Mo3Cr-1100-S-3. The impact velocity is $v_{z} = 371.4~\text {m}/\text {s}$ (impact velocity range 2)

*Fragment*	$L_{\theta }$ (mm)	$L_z~(\text {mm})$	$t~(\text {mm})$	$m~(\text {g})$	$L_{\theta }^{neck}$ (mm)
1	7.00	1.94	1.92	0.075	7.00
2	13.25	1.93	1.93	0.223	13.25
3	16.50	1.95	1.94	0.253	16.50
4	25.00	1.96	1.96	0.369	9.50/9.50/6.00

**Table 53:** List of fragments size and mass for R-Ti5Al5V5Mo3Cr-1100-S-4. The impact velocity is $v_{z} = 369.0~\text {m}/\text {s}$ (impact velocity range 2)

*Fragment*	$L_{\theta }$ (mm)	$L_z~(\text {mm})$	$t~(\text {mm})$	$m~(\text {g})$	$L_{\theta }^{neck}$ (mm)
1	9.50	1.93	1.94	0.149	9.50
2	17.50	1.93	1.92	0.236	17.50
3	33.25	1.92	1.95	0.526	5.00/28.25

**Table 54:** List of fragments size and mass for R-Ti5Al5V5Mo3Cr-1100-S-5. The impact velocity is $v_{z} = 365.4~\text {m}/\text {s}$ (impact velocity range 2)

*Fragment*	$L_{\theta }$ (mm)	$L_z~(\text {mm})$	$t~(\text {mm})$	$m~(\text {g})$	$L_{\theta }^{neck}$ (mm)
1	9.00	1.96	1.92	0.118	9.00
2	9.50	1.95	1.96	0.126	9.50
3	18.25	1.95	1.96	0.277	7.50/10.75
4	26.00	1.95	1.95	0.403	6.50/14.50/5.00

**Table 55:** List of fragments size and mass for R-Ti6Al4V/Ti5Al5V5Mo3Cr-970-D-PL-1. The impact velocity is $v_{z} = 250.7~\text {m}/\text {s}$ (impact velocity range 1)

*Fragment*	$L_{\theta }$ (mm)	$L_z~(\text {mm})$	$t~(\text {mm})$	$m~(\text {g})$	$L_{\theta }^{neck}$ (mm)
1	16.50	1.85	1.84	0.217	16.50
2	44.75	1.88	1.88	0.653	44.75

**Table 56:** List of fragments size and mass for R-Ti6Al4V/Ti5Al5V5Mo3Cr-970-D-PL-2. The impact velocity is $v_{z} = 254.6~\text {m}/\text {s}$ (impact velocity range 1)

*Fragment*	$L_{\theta }$ (mm)	$L_z~(\text {mm})$	$t~(\text {mm})$	$m~(\text {g})$	$L_{\theta }^{neck}$ (mm)
1	63.00	1.89	1.88	0.891	23.00/34.00/6.00

**Table 57:** List of fragments size and mass for R-Ti6Al4V/Ti5Al5V5Mo3Cr-970-D-PL-3. The impact velocity is $v_{z} = 254.9~\text {m}/\text {s}$ (impact velocity range 1)

*Fragment*	$L_{\theta }$ (mm)	$L_z~(\text {mm})$	$t~(\text {mm})$	$m~(\text {g})$	$L_{\theta }^{neck}$ (mm)
1	27.00	1.88	1.87	0.360	10.50/4.00/12.50
2	36.50	1.87	1.85	0.516	36.50

**Table 58:** List of fragments size and mass for R-Ti6Al4V/Ti5Al5V5Mo3Cr-970-D-PL-4. The impact velocity is $v_{z} = 365.2~\text {m}/\text {s}$ (impact velocity range 2)

*Fragment*	$L_{\theta }$ (mm)	$L_z~(\text {mm})$	$t~(\text {mm})$	$m~(\text {g})$	$L_{\theta }^{neck}$ (mm)
1	41.75	1.92	1.86	0.600	41.75

**Table 59:** List of fragments size and mass for R-Ti6Al4V/Ti5Al5V5Mo3Cr-970-D-PL-5. The impact velocity is $v_{z} = 368.5~\text {m}/\text {s}$ (impact velocity range 2)

*Fragment*	$L_{\theta }$ (mm)	$L_z~(\text {mm})$	$t~(\text {mm})$	$m~(\text {g})$	$L_{\theta }^{neck}$ (mm)
1	14.25	1.90	1.85	0.169	8.75/5.50
2	39.50	1.90	1.86	0.573	22.50/17.00

**Table 60:** List of fragments size and mass for R-Ti6Al4V/Ti5Al5V5Mo3Cr-1100-D-PL-5. The impact velocity is $v_{z} = 258.1~\text {m}/\text {s}$ (impact velocity range 1)

*Fragment*	$L_{\theta }$ (mm)	$L_z~(\text {mm})$	$t~(\text {mm})$	$m~(\text {g})$	$L_{\theta }^{neck}$ (mm)
1	7.50	1.92	1.94	0.103	7.50
2	14.00	1.94	1.97	0.187	6.00/8.00
3	14.50	1.92	1.94	0.199	8.50/6.00
4	29.75	1.94	1.92	0.415	18.00/5.50/6.25

**Table 61:** List of fragments size and mass for R-Ti6Al4V/Ti5Al5V5Mo3Cr-1100-D-PL-2. The impact velocity is $v_{z} = 254.8~\text {m}/\text {s}$ (impact velocity range 1)

*Fragment*	$L_{\theta }$ (mm)	$L_z~(\text {mm})$	$t~(\text {mm})$	$m~(\text {g})$	$L_{\theta }^{neck}$ (mm)
1	12.25	1.89	1.94	0.191	12.25
2	21.75	1.89	1.94	0.279	12.25/9.50
3	29.50	1.92	1.97	0.406	6.50/23.00

**Table 62:** List of fragments size and mass for R-Ti6Al4V/Ti5Al5V5Mo3Cr-1100-D-PL-3. The impact velocity is $v_{z} = 368.5~\text {m}/\text {s}$ (impact velocity range 2)

*Fragment*	$L_{\theta }$ (mm)	$L_z~(\text {mm})$	$t~(\text {mm})$	$m~(\text {g})$	$L_{\theta }^{neck}$ (mm)
1	4.25	1.92	1.96	0.066	4.25
2	10.75	1.93	1.95	0.125	4.75/6.00
3	14.50	1.91	1.92	0.160	14.5
4	17.50	1.90	1.94	0.233	17.50
5	19.00	1.91	1.94	0.261	4.50/5.00/9.50

**Table 63:** List of fragments size and mass for R-Ti6Al4V/Ti5Al5V5Mo3Cr-1100-D-PL-4. The impact velocity is $v_{z} = 374.3~\text {m}/\text {s}$ (impact velocity range 2)

*Fragment*	$L_{\theta }$ (mm)	$L_z~(\text {mm})$	$t~(\text {mm})$	$m~(\text {g})$	$L_{\theta }^{neck}$ (mm)
1	10.00	1.90	1.93	0.134	10.00
2	17.25	1.93	1.95	0.150	5.00/12.25
3	12.00	1.92	1.91	0.248	5.50/6.50/5.75
